# Distinct differentiation trajectories leave lasting impacts on gene regulation and function of V2a neurons

**DOI:** 10.1038/s41467-026-76054-w

**Published:** 2026-07-29

**Authors:** Nicholas H. Elder, Alireza Majd, Andrius Cesiulis, Spencer Nyarady, Kalyan Sankar, Emily A. Bulger, Ryan M. Samuel, Lyandysha V. Zholudeva, Todd C. McDevitt, Faranak Fattahi

**Affiliations:** 1 Department of Cellular and Molecular Pharmacology, University of California, San Francisco, San Francisco, CA USA; 2 Eli and Edythe Broad Center of Regeneration Medicine and Stem Cell Research, University of California, San Francisco, San Francisco, CA USA; 3 Developmental and Stem Cell Biology Graduate Program, University of California, San Francisco, CA USA; 4 Gladstone Institute of Cardiovascular Disease, Gladstone Institutes, San Francisco, CA USA; 5 Department of Bioengineering and Therapeutic Sciences, University of California, San Francisco, San Francisco, CA USA; 6 Program in Craniofacial Biology, University of California, San Francisco, San Francisco, CA USA; 7 Present Address: Cellanome, Foster City, CA USA; 8 Present Address: Genentech, South San Francisco, CA USA

**Keywords:** Developmental neurogenesis, Induced pluripotent stem cells, Neural patterning

## Abstract

V2a interneurons are excitatory neurons found throughout the hindbrain and spinal cord, two regions that arise from distinct progenitors during embryonic development. Whether this lineage difference shapes mature gene regulation and function is unknown. V2a neurons show plasticity after spinal cord injury and are candidates for cell therapy. We differentiated human stem cells into V2a neurons through hindbrain- and spinal cord-like progenitor routes and profiled them by single nucleus multiomic sequencing. The two lineages showed distinct transcription factor motif enrichment and differentially expressed genes governing axon growth and calcium handling. Inducing V2a transcription factors directly, bypassing developmental patterning, produced a population unlike either lineage, confirming progenitor history is not interchangeable. Using CellOracle and lentiviral knockdown, we identified CREB5 and TCF7L2 as regulators specific to the spinal-like lineage. These results show that progenitor origin shapes V2a identity and reveal new regulators of neural diversity along the anterior-posterior axis.

## Abstract (web summary)

V2a interneurons in the hindbrain and spinal cord arise from distinct embryonic progenitors. Here they find that hPSCs differentiated to V2a neurons via different progenitors are molecularly distinguishable, demonstrating the importance of progenitor origin for final fate.

## Introduction

Human pluripotent stem cell (hPSC)-derived tissues offer immense potential for disease modeling and novel therapeutic interventions. A major question in the field has been how to best transition pluripotent stem cells into desired cell lineages. This endeavor has led to methods that either mimic developmental processes through “directed differentiations” or rely on transcription factor (TF) overexpression in “induced differentiations”. During development, temporally distinct and spatially separated progenitor populations may give rise to seemingly analogous cell types with common morphological features and expression of hallmark genes. However, these lineally separated populations often differ in tissue localization or functional capacity. Some examples of this include cardiomyocytes arising from the first or second heart fields, melanocytes differentiated from neural crest or Schwann cell-derived precursors^1^ and neurons of the central and peripheral nervous systems^2,3^.

The molecular and functional differences that exist between such analogous cell types can be even larger when comparing cells generated using developmentally relevant strategies to those generated through induced differentiation via TF over expression in stem cells^4–6^. Induced cells can express key marker genes and morphologically resemble the target population, but by nature of having skipped developmental stages, can also misregulate transcription and chromatin marks. These differences necessitate the use of -omics level approaches to deeply characterize the transcriptional and chromatin landscape of cell populations and to facilitate in-depth comparisons between primary cell populations and those generated in vitro.

In this study, we investigate how differentiation trajectories influence the properties of V2a neurons—ventral, excitatory, ipsilaterally-projecting interneurons that emerge along the anteroposterior axis and play key roles in phrenic and locomotor circuits^7–11^. V2a neurons exhibit plasticity in restoring diaphragm function and are a key target population for the recovery of locomotion via electrical stimulation after spinal cord injury^12–14^. Transplantation of V2a neurons also has potential as a cell therapy to enhance functional recovery after spinal cord injury, making this cell type of high therapeutic interest^15–17^.

V2a neurons develop over the entire axial range of the neural tube and understanding how axial positioning intersects with their identity as a V2a neuron is critical for deriving therapeutically relevant cell types in vitro^11,18,19^. In vivo and in vitro, axial patterning is one of the first steps of development, preceding much of differentiation^20^. Anterior V2a neurons primarily arise from neurectoderm progenitors (NEPs), which are induced by the inhibition of BMP and TGF signaling in pluripotent stem cells. By contrast, posterior V2a neurons are formed from a distinct population termed neuromesodermal progenitors (NMPs), which contribute to the posterior elongation of the embryo as they differentiate into the neural tube and adjacent somites^21–24^. Despite differences in early development, subsequent dorsoventral patterning of the neural tube is remarkably conserved along the anteroposterior axis, first specifying several discrete progenitor domains, including the p2 domain that gives rise to the V2 class of interneurons. As the p2 cells become post-mitotic, differential Notch activity specifies between the two major V2 classes of neurons: excitatory V2a neurons that express the canonical marker VSX2 (also known as CHX10) and inhibitory V2b neurons that express GATA2, GATA3, and TAL1^25–29^. However, the anteroposterior origin of V2a neurons could lead to molecular differences with potential functional implications. Indeed, V2a neurons exhibit different patterns of axon extension as well as long-term expression of the V2a marker VSX2 as a function of their location within the spinal cord in model organisms^11,18^.

Here, we interrogate how early differences in developmental patterning and progenitor identity impact the resulting V2a phenotype using three distinct models of hPSC differentiation. We first established a system to generate V2a neurons from NMPs to compare their molecular and functional features with their NEP-derived counterparts. We then used multiomic single-nucleus RNA and ATAC-seq approaches to examine how differences in the early developmental lineage are imprinted into the chromatin landscape and transcriptional signature of these neurons. We found that while both NEP-derived and NMP-derived V2a populations were similar to human tissue-derived V2a neurons, the two in vitro-derived populations exhibited notable differences in gene expression and regions of open chromatin stemming from their distinct progenitor origins. Functional profiling of the neurons supported the differences observed in multiomics data, showing NMP-derived populations established network-wide activity more rapidly and consistently than NEP-derived neurons.

To further define the impact of differentiation trajectories on V2a neurons, we also established a protocol for induced differentiation which skips normal developmental patterning and rapidly transitions stem cells to post-mitotic neurons. We find that induced VSX2-expressing neurons greatly differ from both of our NEP and NMP-derived V2a populations, express genes not typically associated with V2a neurons, and shift their identity even after progressing to a post-mitotic state. Lastly, leveraging our multiomics dataset, we employed in silico gene regulatory network (GRN) modeling and perturbation to show how different TFs regulate lineage-specific differences between NEP and NMP-derived V2a populations. Our results highlight the significance of developmentally accurate directed differentiation strategies to generate relevant and authentic cell types for developmental and disease modeling and therapeutic application.

## Results

### Distinct neural progenitors can generate V2a neurons

**Fig. 1:**
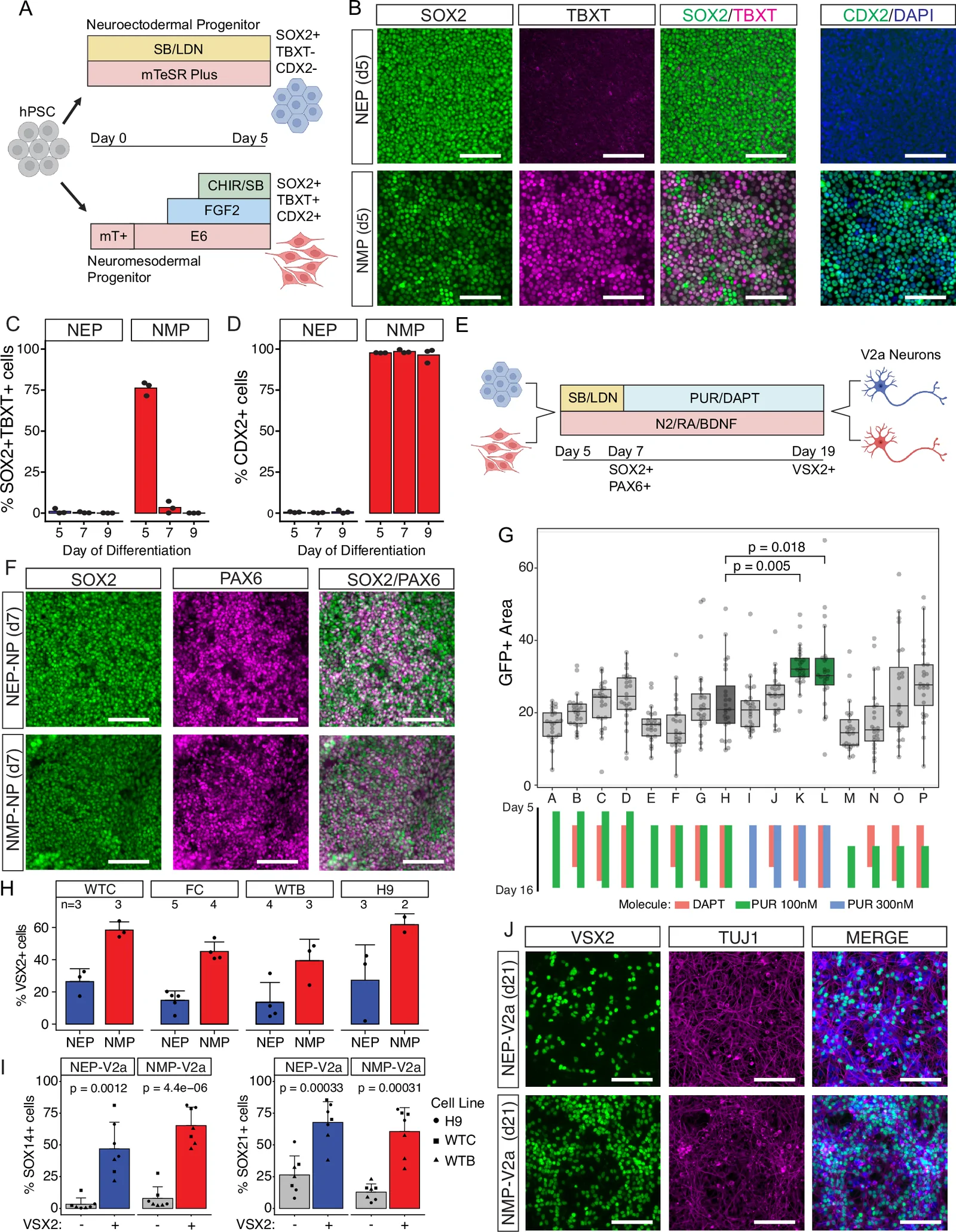
Generation of V2a neurons from distinct progenitor lineages. **A** Schematic of the first five days of differentiation from human pluripotent stem cells toward neural progenitors under defined media conditions with expected marker gene expression. SB SB431542, LDNLDN193189, CHIR CHIR99021, E6 Essential 6, mT+ mTeSR Plus. Figure created with BioRender. https://BioRender.com/l10a099. **B** Representative expression of neuromesodermal progenitor markers SOX2, TBXT, and CDX2 at day 5 in NEP and NMP conditions (top and bottom rows). Scale bars, 100 μm. **C** Flow cytometry quantification of the mean percent of cells co-expressing SOX2 and TBXT in NEP and NMP populations at days 5, 7, and 9. *n* = 3 biological replicates. **D** Flow cytometry quantification of the mean percent of CDX2+ cells in both progenitor populations at days 5, 7, and 9. *n* = 3 biological replicates. **E** Schematic of differentiation of distinct progenitor lineages toward V2a neurons under identical defined media conditions from day 5 to day 19 with expected marker gene expression. PUR purmorphamine, RA retinoic acid. Figure created with BioRender. https://BioRender.com/l10a099. **F** Representative expression of SOX2 and PAX6 in NEP-derived (NEP-NP) and NMP-derived (NMP-NP) neural progenitors at day 7 (top and bottom rows). Scale bars, 100 μm. *n* = 3 independent experiments. **G** Differentiation optimization using FOXN4:Cre reporter line. Boxplots show GFP+ area for PUR and DAPT conditions between days 5 and 16. Condition L (300 nM PUR) was selected for further experiments. One-way ANOVA with Tukey HSD. Boxplot center line = median; bounds = first and third quartiles; whiskers = 1.5× the interquartile range. *n* = 24. **H** Flow cytometry of VSX2+ cells at day 19 from NEP and NMP lineages across four human stem cell lines. WTC WTC11, FC FOXN4:Cre. Error bars, mean ± standard deviation. **I** Flow cytometry quantification of the percent of SOX14+ and SOX21+ cells in VSX2-positive and -negative populations at day 19. Two-sided *t*-test. *n* = 7 biological replicates across three cell lines. Error bars, mean ± standard deviation. **J** Representative expression of VSX2 and TUJ1 in NEP-V2a and NMP-V2a neurons at day 21. Scale bars, 100 μm. *n* = 3 independent experiments. Source data are provided as a Source Data file.

To better understand how the axial identity influences mature V2a cell identity, we began by deriving NEPs and NMPs using previously established protocols before maturing these progenitor populations to V2a neurons under identical conditions^30–32^. Mirroring the conditions of in vivo development, hPSCs are converted into NEPs via dual SMAD inhibition in FGF-containing stem cell media for the first 5 days (Fig. 1A; top method). NMPs are induced in vivo and in vitro by balanced FGF and WNT signaling and are identified by co-expression of SOX2 and TBXT (also known as Brachyury), as well as the caudal homeodomain protein CDX2. We performed an NMP induction protocol (Fig. 1A; bottom method)^32^ that resulted in high levels of SOX2 and TBXT coexpression and >90% CDX2 expression at day 5 (Fig. 1B–D). Notably, TBXT and CDX2 were completely absent in the NEP condition at a matched time point (Fig. 1B–D).

Following NEP or NMP induction over the first 5 days of differentiation, the progenitor populations were subsequently differentiated toward V2a interneurons under identical media formulations (Fig. 1E). Since the neural tube patterning is highly conserved between anterior and posterior regions in vivo, identical treatment from day 5 onward allowed us to specifically investigate the impact of early progenitor origins on the resulting neurons. We compared the differentiated populations at day 7 following 48 h of dual SMAD inhibition in the presence of 100 nM RA, a potent neuralizing morphogen. At this time-point, both populations began to co-express canonical neural progenitor markers SOX2 and PAX6, while TBXT levels in the NMP-derived population dropped significantly (Fig. 1C, F). CDX2 remained highly expressed in the NMP-derived progenitors (Fig. 1D). These data show the efficient generation of neural progenitors via two different differentiation routes that converge on a SOX2 + PAX6+ neural phenotype.

Prior studies have generated V2a neurons from neural progenitors via balanced control over three developmental signaling pathways: retinoic acid, Sonic hedgehog (SHH), and Notch^30,31^. Retinoic acid generated by the paraxial somites and SHH secreted by the floor plate (FP) serve to pattern the neural tube, while the Notch pathway controls the balanced acquisition of excitatory V2a and inhibitory V2b fates from p2 progenitors. In vitro, the small molecule agonist purmorphamine (PUR) is substituted for SHH, while the gamma-secretase inhibitor DAPT potently inhibits Notch signaling and biases p2 progenitors exclusively toward the V2a fate resulting in almost no V2b cells as would be identified by GATA2, GATA3, and TAL1 expression^29^. To further optimize the timing and concentration of PUR and DAPT to yield increased V2a neurons in our differentiations, we generated a Cre lineage reporter under the control of the TF FOXN4, which is expressed solely in p2 progenitors (See Supplemental Note and Supplementary Data 1 and 2). This lineage reporter (called FOXN4:Cre or abbreviated FC) allowed us to observe the efficiency of p2/V2a differentiation in live cultures through the readout of a GFP reporter. We used the FOXN4:Cre line to test 16 iterations of the differentiation protocol, where we varied the timing or concentration of PUR and DAPT (Fig. 1G). We found that 300 nM PUR added from day 7 onward significantly increased the GFP+ area over our control condition with 100 nM PUR (Fig. 1G). Therefore, this modification was used throughout the rest of this study.

On day 19 of V2a differentiation, cells were dissociated and assessed for the canonical V2a marker VSX2 via flow cytometry (Fig. 1H). While VSX2 + V2a neurons were detected in all differentiations across four independent cell lines, the NMP condition consistently produced higher percentages of V2a neurons under our experimental conditions (Fig. 1H). We further validated V2a identity by flow cytometry for secondary markers SOX14 and SOX21 and observed significant enrichment of each marker in the VSX2+ population (Fig. 1I). Finally, cells that were replated and allowed to grow for an additional two days generated extensive networks of neurites, supporting neural identity (Fig. 1J). These results confirm the V2a neuron identity in both the NEP- and NMP-derived cultures.

### Differentiated neurons recapitulate ventral tube identities

**Fig. 2:**
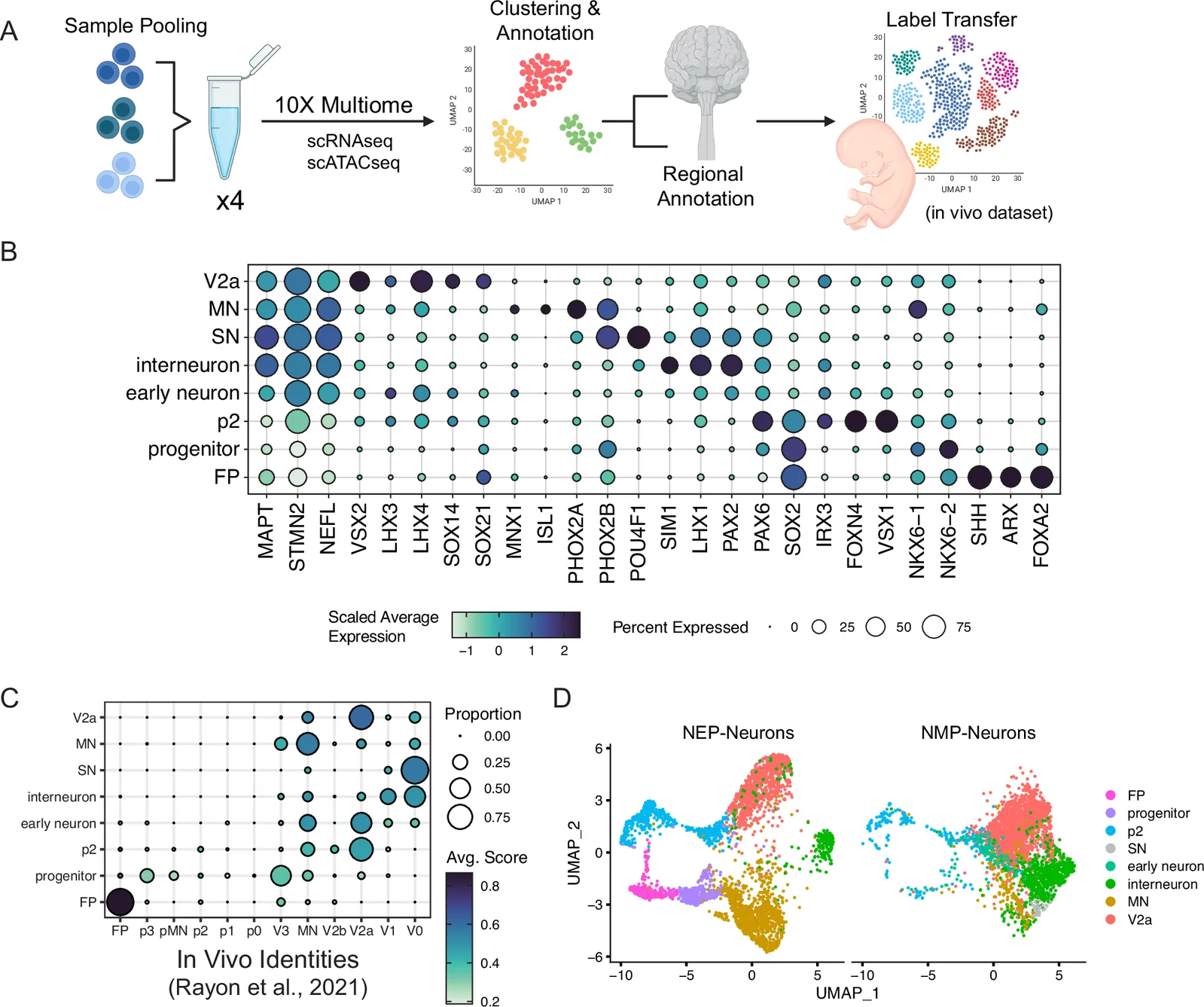
Distinct progenitor lineages recapitulate cellular diversity of primary human neural tube. **A** Schematic of sample pooling for multiome and cell annotation steps. Figure created with BioRender. https://BioRender.com/l10a099. **B** Expression of selected marker genes for the eight annotated cell types in a merged object of all of multiome samples. **C** Results of an unbiased label transfer of multiome samples onto ventral human fetal spinal cord cells. **D** UMAP of annotated multiome samples after Harmony integration.

Having established neural populations with a canonical V2a identity (VSX2+/SOX14+/SOX21+) via both differentiation methods and across multiple cell lines, we examined whether epigenetic and gene regulatory patterns differed based on early progenitor lineage. To address these questions, we performed single-nucleus multiome sequencing after 19 days of differentiation (Fig. 2A).

We first used the snRNA-seq data to cluster and annotate cell types according to known markers (Figs. 2B and S1 and Supplementary Data 3). V2a neurons expressed *VSX2*, *LHX3*, *LHX4*, *SOX21*, and *SOX14*. Motor neurons (MN) were identified by *PHOX2A*, *PHOX2B*, and *NKX6.1* expressing or *MNX1* (also known as *HB9*) and *ISL1* expression, representing cranial or spinal identities, respectively. We noted that MN in the NEP-derived conditions were almost exclusively cranial-like, while NMP-derived populations had a mix of the two (Fig. S2A). Overlap of *PHOX2B* and *POU4F1*, detected in one NMP-derived sample, was suggestive of a sensory neuron population, possibly arising from a small population of neural crest cells arising during NMP differentiation (Figs. S1G, H and S2B). Non-V2a ventral interneurons, such as V3, V1, and V0 neurons clustered together as seen by expression of a mix of ventral markers, including *SIM1*, *LHX1*, and *PAX2* were labeled “interneurons”. We also identified a large population of p2 progenitors that expressed *PAX6*, *IRX3*, *FOXN4*, and *VSX1*. Other cells expressed primarily *SOX2* and *NKX6.2*, which are widely expressed across the ventral progenitor domains and were therefore broadly termed “progenitors”. Similarly, a population that expressed neural genes *STMN2*, *NEFL*, and *MAPT* but lacked specific population markers were called “early neurons”. Lastly, highly specific expression of *SHH*, *FOXA2*, and *ARX* indicated the presence of FP cells.

While flow cytometry at day 19 showed ~20% VSX2+ cells in NEP and ~50% in NMP cultures (Fig. 1H), single-nucleus RNA-seq recovered lower absolute proportions of V2a neurons (Fig. S2B), likely reflecting neuronal fragility during cryopreservation and nuclear isolation, as well as differences between protein and RNA detection. Critically, the proportional enrichment of V2a neurons in NMP- versus NEP-derived cultures was consistent across both methods. Of other cell types, only the NEP condition contained a FP population. There was also a noticeable shift in the proportion of MNs and interneurons between the NEP and NMP conditions. Although both conditions were treated with the same concentration and duration of PUR to produce ventral identities, there is a stronger ventral bias in the identities in the NEP-derived samples compared to those of the NMP-derived condition. This ventral shift is seen in the presence of the FP and proportionally larger MN populations, which are ventral to the p2/V2 domain in vivo (Fig. S2B).

We also examined which anteroposterior markers were expressed in our NEP and NMP conditions (Fig. S2C). We observed low expression of off-target forebrain, midbrain, and eye markers such as *FOXG1*, *OTX2*, and *CRX* (Fig. S2C). In the NEP-condition, *HOX* expression primarily ranged from *HOX* 1–5 paralogs, analogous to a hindbrain identity. NMP-derived samples also expressed these relatively anterior paralogs in addition to more caudal *HOX* genes such as *HOXC5*, *HOXC6*, and *HOXB9*, consistent with a cervical spinal cord identity (Fig. S2C). This assessment of axial markers supports a model where the NEP-derived V2a neurons more closely resemble hindbrain populations, while NMP-derived neurons resemble spinal populations

To further confirm our assigned cell identities, we performed an unbiased single-cell label transfer between our cells and the ventral spinal cell types annotated in an in vivo human fetal dataset^33^. Label transfer results largely agreed with our annotations, with the exception of the in vitro p2 progenitors, which mapped more strongly onto in vivo V2a neurons than in vivo p2 progenitors (Fig. 2C). Further, the majority of our cells were identified as more mature neurons instead of progenitors, consistent with a largely post-mitotic neuronal state. Harmony integration of our RNAseq datasets clustered analogous cell types closely to one another, but did not entirely intermingle the NEP and NMP conditions (Fig. 2D). Next, because we were specifically interested in the V2a neurons, we sought to determine whether the NEP-derived or NMP-derived V2a neurons were more similar to the in vivo neurons. After sub-setting each sample to only the V2a neurons, we found that principal component 3 (PC3) accounted for NEP and NMP sample variation with in vivo sample overlap (Fig. S3A–D). Module scoring using the gene lists positively and negatively correlated with PC3 showed in vivo V2a neurons scoring more similarly to NMP-V2a neurons (Fig. S3E). Altogether, this analysis affirms our ability to identify regionally relevant cell types across all samples, including V2a neurons, correlate them with in vivo samples, and highlights the axial differences between populations derived from NEP or NMP conditions.

### Lineage shapes epigenetic and transcriptional V2a identity

**Fig. 3:**
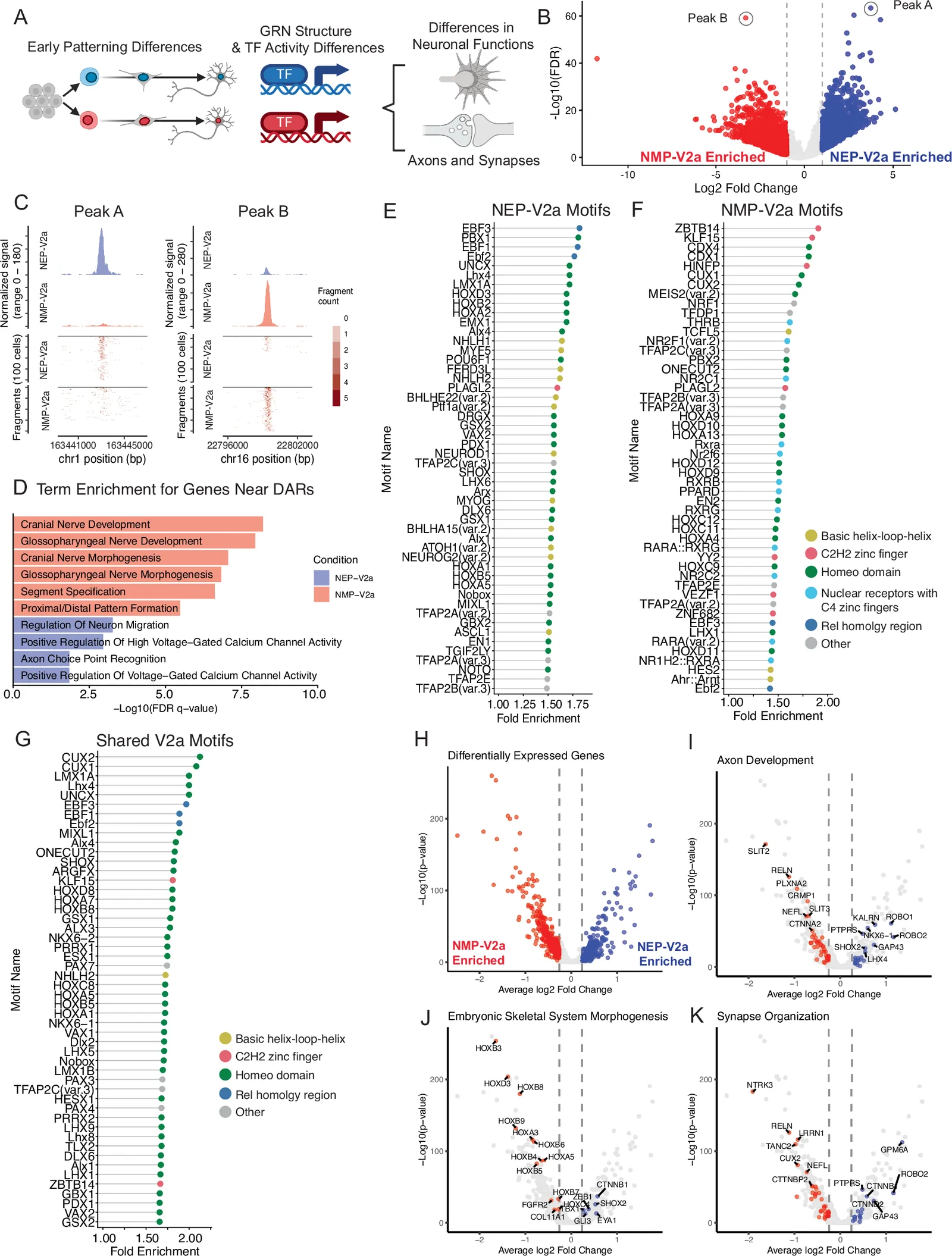
Progenitor lineage confers distinct transcription factor binding motifs and gene expression. **A** Schematic of the V2a differentiation and differences identifiable using multiomic data. Figure created with BioRender. https://BioRender.com/l10a099. **B** Volcano plot of differentially accessible chromatin regions (DARs) between NEP- and NMP-derived V2a neurons in snATAC-seq data. Blue, NEP-enriched peaks (positive log2 fold change); red, NMP-enriched peaks (negative log2 fold change). Two-sided logistic regression with FDR-adjusted *p*-values. **C** Coverage plot of example peaks specific to NEP or NMP conditions (left and right). Shown as normalized histograms or fragment count heatmaps per 100 cells (top and bottom). **D** Selected significantly upregulated gene ontology terms for NEP- or NMP-specific DARs (GREAT analysis). **E**, **F** Top 50 enriched transcription factor motifs in NEP-V2a (**E**) or NMP-V2a (**F**) differentially accessible regions, colored by transcription factor class. **G** Top 50 enriched transcription factor motifs in conserved V2a-specific DARs identified in both NEP- and NMP-V2a populations, colored by transcription factor class. **H** Volcano plot of differentially expressed genes between NEP- and NMP-derived V2a neurons in snRNA-seq data. Blue, NEP-enriched genes (positive log2 fold change); red, NMP-enriched genes (negative log2 fold change). Two-sided Wilcoxon rank-sum test with Bonferroni-adjusted *p*-values. **I**–**K** Differentially expressed genes belonging to the indicated gene ontology (GO) term. Two-sided Wilcoxon rank-sum test with Bonferroni-adjusted *p*-values. Source data are provided as a Source Data file.

Early developmental patterning events play a crucial role in molding the chromatin to position cells for subsequent stages of development. Therefore, we leveraged our snATAC-seq data to examine whether the NEP and NMP differentiation stages left long-term marks on the chromatin landscape in ways that could influence transcriptional output and neuronal function (Fig. 3A). To focus on the V2a population, we subset all V2a cells (2672 cells) and performed differential peak analysis. Out of 60732 accessible regions in V2a neurons, this identified 7128 peaks (log2FC > 1, FDR < 0.05) that were differentially accessible between NEP and NMP V2a populations (Fig. 3B). Examination of top differential peaks showed that peak sets included mutually exclusive regions with highly differential accessibility, not only peaks with differences in relative accessibility (Fig. 3B, C).

Analysis of differentially accessible regions (DARs) did not identify differences in the relative location of peaks within genes, the distance from the nearest transcriptional start site, or the gene type annotation (protein coding, miRNA, etc.) of the nearest transcription start site (TSS) between NEP and NMP conditions (Fig. S4). However, motif enrichment analysis of the DARs between NEP- and NMP-derived V2a neurons did identify dozens of significantly enriched TF-binding motifs (Fig. 3E, F). In order to identify broad patterns, we annotated TF motifs by class, since many related TFs bind similar motifs. In the NEP condition, motifs of the Rel homology region class were the most highly enriched (EBF1/2/3), followed by anterior HOX and LIM homeodomains and basic helix-loop-helix (bHLH) class motifs (Fig. 3E). In the NMP condition, C2H2 zinc-finger and nuclear receptor motifs were most strongly enriched, followed closely by homeodomain motifs (Fig. 3F). The nuclear receptor class contains retinoid-related TFs (e.g., RXRA, RXRB, RARA), which suggests a differential action of retinoic acid in the NMP condition. While both conditions have enriched homeodomain class TFs, CDX, and CUX family motifs are more prevalent in the NMP condition, while NK-related and Paired-related homeodomain family motifs are more highly enriched in the NEP condition. The bHLH family of TFs are known to be key regulators of neurodevelopment, and several of these factors are specifically expressed during V2a development (NEUROD1, NEUROG2, ASCL1, BHLHE22)^34,35^. However, this TF class was specifically enriched in the NEP condition (Fig. 3E). A notable shift in the anterior-posterior identity of enriched HOX family motifs led us to specifically interrogate all four *HOX* clusters for differentially accessible peaks. We identified 35 NMP-enriched peaks encompassing all four HOX clusters, lending further support to these V2a populations’ distinct axial identities (Fig. S6A–D). We also tested for gene ontology enrichment using the Genomic Regions Enrichment of Annotations Tool (GREAT) to see whether nearby genes were associated with specific functions. NEP V2a neurons possessed a modest enrichment for terms such as “regulation of glutamatergic synaptic transmission” and “regulation of voltage-gated calcium channel activity”, while NMP V2a neurons had strong enrichment for broad terms related to regionalization, anterior/posterior patterning, and nerve development (Figs. 3D and S5A, B). We also identified 3046 peaks of the global set of 381512, which were enriched in the V2a population of both conditions relative to all other cells of that condition. These shared V2a-specific peaks were enriched for homeodomain motifs, especially Paired-related homeodomain factors, of which VSX2 is a member, and several genes involved in V2a differentiation, such as LHX4, NKX6.1, and NKX6.2 (Fig. 3G)^33,36^. These results show that the progenitor lineage of V2a neurons leads to lasting differences in the chromatin landscape even after two weeks of identical treatment. Further, the motifs enriched in differentially accessible regions correspond to TF families with known roles during V2a development and suggests that they may contribute to distinct gene regulation in each condition.

We next analyzed differences in the transcriptional networks between NEP- and NMP-derived V2a neurons. Using the same set of V2a neurons, we identified 742 differentially expressed genes (Fig. 3H; log2FC > 0.25 and Bonferroni-adjusted *p*-value < 0.05). Consistent with our earlier findings, *HOX* genes were differentially expressed, reflecting distinct axial identities. Notably, differences include TFs, ion channels, and axon guidance molecules related to GO terms such as axon development, synapse organization, and embryonic skeletal system morphogenesis (Fig. 3I–K). GO analysis of the differentially expressed genes identified 22 significantly enriched GO terms that are shared between the NEP and NMP conditions, yet these enrichments are driven by mutually exclusive gene sets (Fig. S5C). These findings suggest that NEP- and NMP-V2a neurons use distinct gene modules to achieve similar processes, such as axonogenesis, potentially leading to different circuit architecture of neurons residing at different axial levels of the hindbrain and spinal cord. When taken with the snATAC-seq analysis, these data point to systematic chromatin changes underlying distinct transcriptional networks of V2a populations derived from NEP or NMP progenitor pools.

We next asked whether the observed differences in motif enrichment were also present in the progenitors to the V2a neurons, the p2 cells, or whether it only manifested with terminal differentiation. We therefore subset the p2 cells (781 cells) from our dataset and performed similar analyses as above. This identified 5239 DARs out of 74474 shared peaks. Motif enrichment in NEP-p2 compared to NMP-p2 DARs mirrored the trends observed in the V2a neurons (Fig. S7A, B). DARs in NEP-p2 cells were enriched in bHLH motifs, while NMP-p2 peaks were enriched in C2H2 zinc finger motifs and some homeodomain motifs, including CDX family genes. When we compared the DARs specific to the NEP or NMP lineage in both p2 and V2a populations, only 7.8% of NEP-specific DARs were shared between p2 and V2a neurons, while 13.2% of the NMP-specific DARs were conserved from p2 progenitors to V2a neurons (Fig. S7C, D). Comparing the global ATAC profile of p2 and V2a populations with a Pearson correlation showed the highest degree of similarly within cell identity, not progenitor lineage (Fig. S7E). These data show that most differentially accessible peaks are not carried forward in development from p2 progenitors to V2a neurons, but that the enriched TF motifs are more conserved. This suggests that the genomic targets unique to a distinct lineage can change over the course of development. However, whether a single TF is continuously expressed or whether successive family members hand off regulatory responsibility at each developmental transition remains an open question.

### Population-wide synchronous activity differs with maturation

We next sought to examine potential functional differences between NEP- and NMP-derived neuronal cultures. Dissociated cultures were replated at day 19 at a uniform density and allowed to form new networks. Though we attempted to isolate pure populations, V2a neurons did not recover from cell sorting, so the full heterogenous population, including MNs and other interneurons, was replated and used in activity studies.

**Fig. 4:**
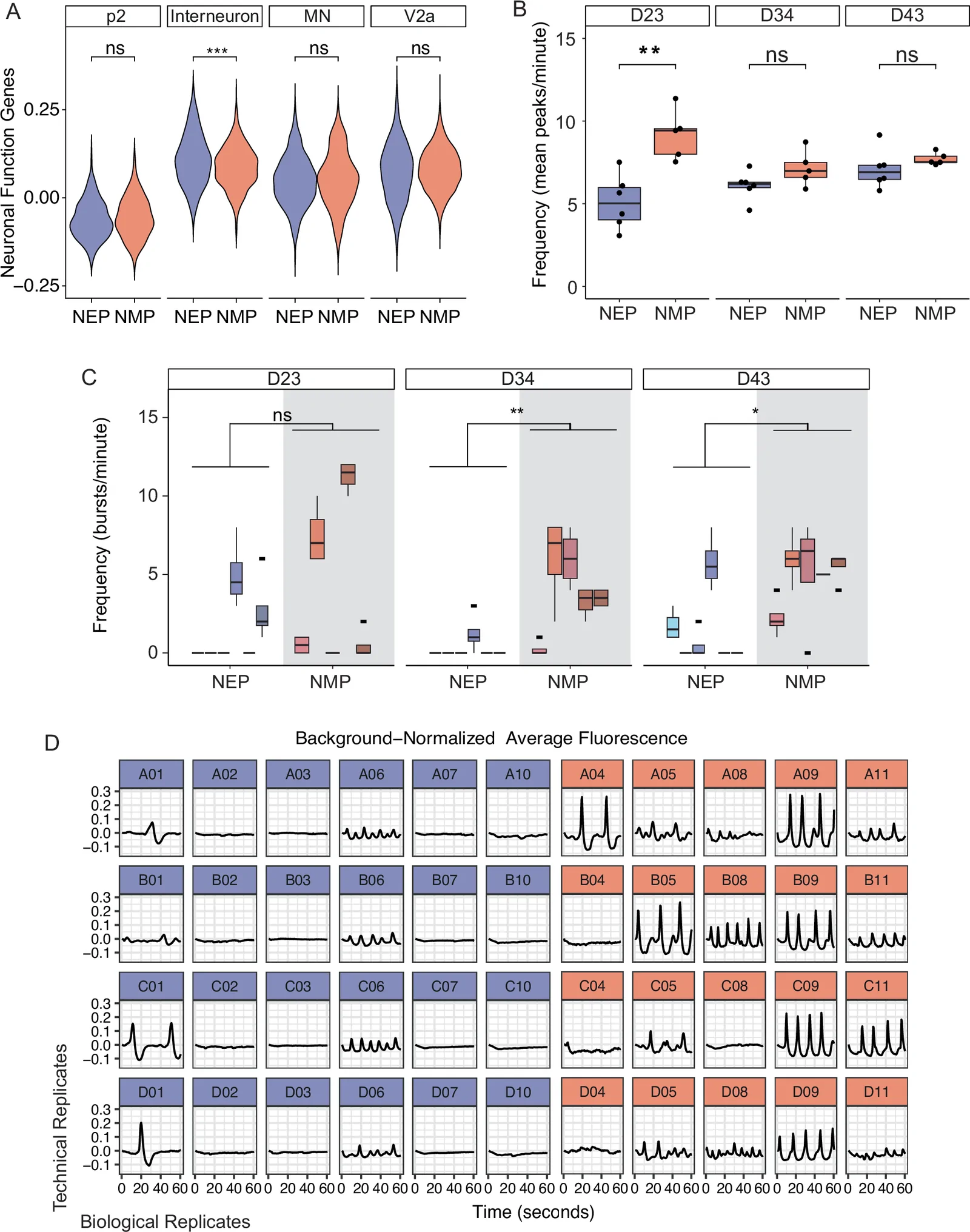
NMP-derived neurons generate robust synchronous activity in maturing cultures. **A** Module scores for mature neuron functional genes in day 19 NEP- and NMP-derived cell populations conserved across all samples. Two-sided *t*-test with Bonferroni correction. ***p = 0.0003. **B** Mean number of calcium peaks per region of interest (ROI) in replated V2a cultures at indicated time points, measured by Fluo4-AM imaging. *n* = 6 NEP samples; *n* = 5 NMP samples. **p = 0.002, two-sided *t*-test. Boxplot center line = median; bounds = first and third quartiles; whiskers = 1.5× the interquartile range; points = mean of four technical replicates per sample. **C** Number of synchronous bursts per well in replated V2a cultures at indicated time points, measured by Fluo4-AM imaging. *n* = 4 technical replicates per biological sample. *p = 0.043, **p = 0.0072, two-sided Wilcoxon rank-sum test. Boxplot center line = median; bounds = first and third quartiles; whiskers = 1.5× the interquartile range; points = individual replicates. **D** Background-normalized average fluorescence per well at day 43. NEP samples in blue, NMP samples in red. Columns = biological replicates; rows = technical replicates. Source data are provided as a Source Data file.

Since differences in neuron maturity can influence activity, we sought to characterize the maturation status of the NEP and NMP-derived populations. We first examined a time course of qPCR data from day 5 to 19 that showed similar kinetics of marker gene gain and loss throughout differentiation (Fig. S8). We also used our scRNA-seq data at day 19 to examine maturity module scores across four cell populations and found that only non-V2a interneurons in the NEP lineage had a small but significant increase in score, and the rest were non-significant (Fig. 4A). Thus, it appears that day-matched NEP and NMP cultures do not have baseline differences in maturity at day 19.

With the input cell populations closely maturity-matched, we next assessed spontaneous activity using the cell-permeable dye Fluo4-AM. At the day 23 timepoint, spontaneous neuronal activity was observed in both conditions, with the NMP-derived cultures eliciting a higher average number of fluorescence peaks per ROI (Fig. 4B and Movies S1 and S2). However, at day 34 and day 43, the average number of peaks per ROI had equalized and become non-significant (Fig. 4B). These results show that when ROI are assessed individually, populations in both conditions have approximately the same amount of activity as measured by peaks in Fluo4 fluorescence.

Examination of the average activity across all ROI also revealed synchronous bursts of activity across the whole field of view, which occurred simultaneously or in rapidly propagating waves. While there was no significant difference in these bursts between NEP and NMP-derived neurons at day 23, we observed significantly more synchronous bursts in NMP-derived cultures at later time points (day 34 and day 43) compared to NEP cultures (Figs. 4C, D and S9A, B and Movies S3 and S4). Although we cannot purely attribute this activity to V2a neurons alone, excitatory neurons have a demonstrated role in patterned or bursting activity^37–39^. We noted that several of the differentially expressed genes between the NEP and NMP V2a neurons at day 19 include genes related to synapse organization and synaptic transmission (Fig. 3H, K). In addition, among the most highly differentially expressed genes were subunits of cation channels, which could also impact synaptic transmission (*CACNA2D1* and *RYR2* in the NEP-V2a condition; *CACNA2D3* and *KCND2* in the NMP-V2a condition). While requiring more in-depth study in the future, this experiment shows that lineally distinct spinal neuron populations may not only possess unique epigenetic and transcriptomic hallmarks, but also functional differences that could influence their role in vivo and in therapeutic uses.

### Induced VSX2+ neurons differ from NEP or NMP-V2a neurons

**Fig. 5:**
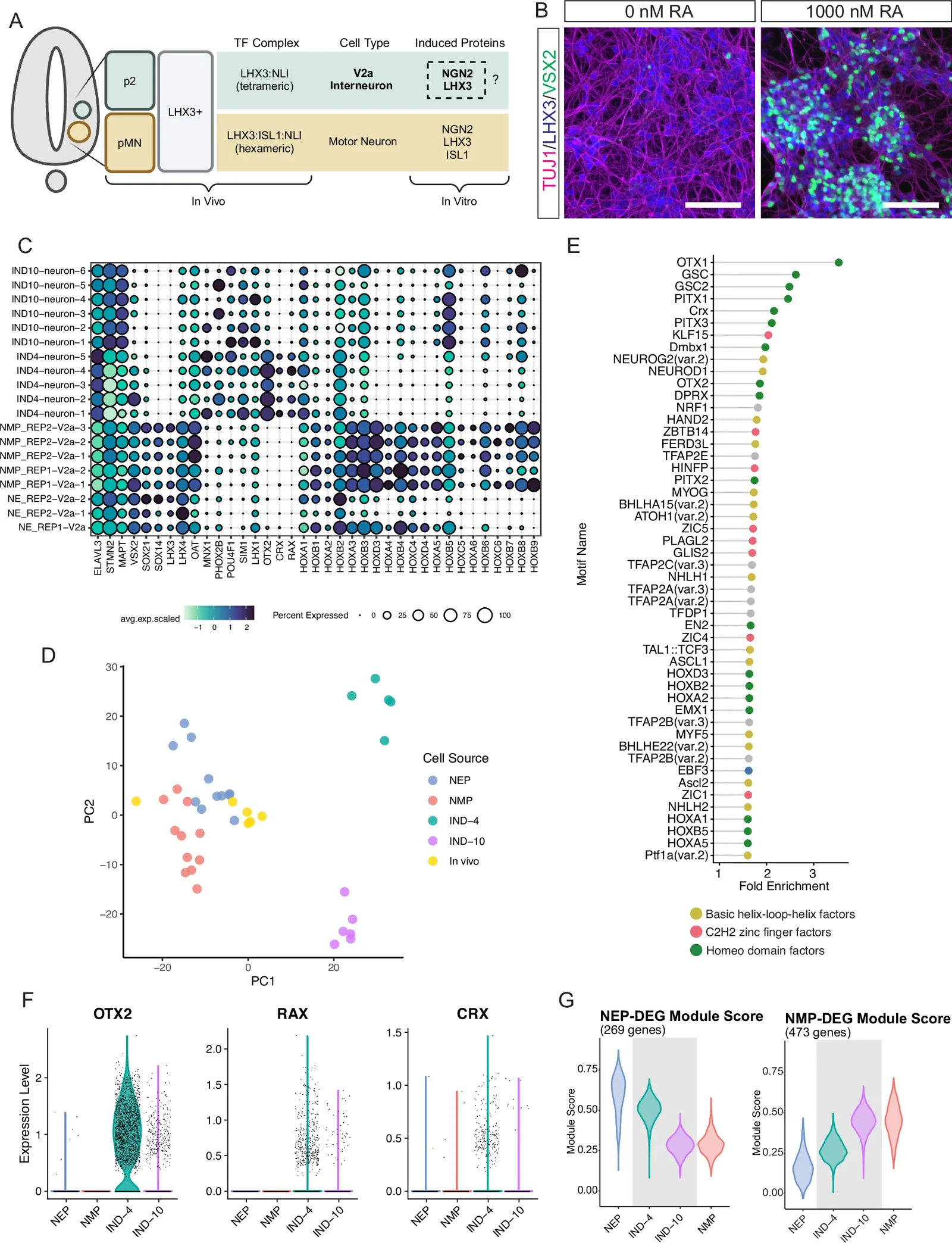
Induced neurons express anterior CNS markers and fail to recapitulate in vivo neural identities. **A** Schematic of transcription factor complexes governing V2a and motor neuron fate specification in the developing neural tube, and transcription factor combinations used in vitro. Figure created with BioRender. https://BioRender.com/l10a099. **B** Representative immunostaining of NGN2::LHX3 induced neurons at day 4 with 0 or 1000 nM RA. LHX3 (blue), TUJ1 (magenta), VSX2 (green). Scale bar, 100 μm. *n* = 3 independent experiments. **C** Marker gene expression across NEP-V2a, NMP-V2a, and day 4 and day 10 induced neuron clusters, including pan-neural, V2a, off-target, and axial identity genes. **D** PCA of pseudobulked clusters from NEP, NMP, in vivo, and day 4 and day 10 induced neuron populations. **E** Top enriched transcription factor motifs in differentially accessible chromatin regions specific to day 4 induced neurons relative to NEP- and NMP-V2a neurons, ranked by fold enrichment and colored by transcription factor class. **F** Expression of anterior markers OTX2, RAX, and CRX across differentiation conditions. **G** Module scores for indicated neuron populations using gene modules composed of differentially expressed genes specific to NEP- or NMP-V2a populations. Source data are provided as a Source Data file.

Recently, the use of TF overexpression to rapidly produce induced neurons has gained widespread popularity in the field^4–6,40^. This approach is highly efficient, scalable, and allows for the generation of neuron-like cells in just a few days, bypassing much of the complex developmental patterning typically required. Our data suggest that the divergent developmental trajectories of neural progenitors influence the epigenetic and transcriptional landscapes of mature V2a neuron. We therefore asked whether induced neurons that passed through minimal developmental patterning could share characteristics with either the NEP- or NMP-V2a populations. Similarity between NEP-V2a or NMP-V2a neurons and an induced neuron would imply a “default” state of differentiation or that developmental processing was secondary to a final gene expression network that defined a V2a neuron, while major differences would point to the necessity of developmental progression or other external cues in creating a V2a-like transcriptional network. Based on the developmental similarities between V2a and lower MN, we hypothesized that removing ISL1 from the tripartite induced MN cassette, including NGN2, LHX3, and ISL1, could induce V2a neurons, since ectopic expression of LHX3 alone induces V2a neurons in vivo (Fig. 5A)^25,41,42^.

Thus, we generated a plasmid containing a doxycycline-inducible NGN2::P2A::LHX3 cassette and inserted it into WTC11 stem cells via piggybac transposon-mediated integration. Following 4 days of induction, we performed flow cytometry for VSX2. However, <1% of cells were positive for VSX2, despite achieving the expected neural morphology (Figs. 5B and S10A). Since pluripotent stem cells default to an anterior fate, we hypothesized that our induced neurons may need an additional caudalizing signal, such as retinoic acid (RA)^20,43,44^. When we added graded concentrations of RA to our induced neuron differentiation protocol, we found a proportional and significant increase in the percentage of VSX2+ cells after 4 days of induction, with a maximum efficiency of ~40% VSX2+ neurons at 1 uM RA (Figs. 5B and S10A). Notably, when we tested identical conditions on the NGN2 induced neurons and the induced MNs, the number of VSX2+ neurons remained <1%.

We characterized day 4 and day 10 induced V2a neurons with multiome sequencing to assess their identity. While we detected *VSX2+* clusters at day 4, these cells lacked expression of other V2a markers, such as *SOX21*, *SOX14*, and endogenous *LHX3* (Figs. 5C and S10B, C). The VSX2− clusters were largely positive for the MN marker MNX1 (Figs. 5C and S10B, C). While this was not the target population, the shared LHX3+ lineage of both cell types in vivo supports the idea that there could be a balance between these populations produced by overexpression of LHX3 in stem cells. More unexpected was the observation of *PHOX2B* and *POU4F1* overlapping *VSX2* and *MNX1* populations, respectively, as well as the presence of other interneuron markers *SIM1* and *LHX1* (Figs. 5C and S10C, E). Expression of *PHOX2B* and *POU4F1* have been described in studies of NGN2-induced neurons and may be a product of the induced method^45^. It is unclear if *SIM1* and *LHX1* are expressed under the same conditions. Nevertheless, these markers persist through day 10 and overlap remaining *VSX2+* and *MNX1+* clusters. To define the global similarities between induced and directed NEP and NMP neurons generated in vitro, as well as to in vivo spinal neurons, we pseudobulked the RNA data from neuron clusters and embedded them in PCA space (Fig. 5D). The PCA showed that both of the induced time points clustered distantly from the NEP, NMP, and in vivo clusters along PC1, as well as distantly from each other along PC2. By contrast, both NEP and NMP samples were close to in vivo clusters.

The motifs enriched in the differentially accessible peaks between day 4 induced neurons and combined NEP and NMP-V2a neurons showed an enrichment of anterior homeodomain motifs such as GSC, CRX, and OTX1/2 in addition to the expected bHLH motifs from NGN2 overexpression (Fig. 5E). At day 4, we noticed significant expression of the anterior gene *OTX2* along with lower levels of eye-related factors *CRX* and *RAX* while *HOX* gene expression mostly stopped at *HOXB2* (Fig. 5C, F). However, day 10 induced neurons downregulated *OTX2*, *CRX*, and *RAX* and increased expression of *HOXB5* and *HOXB8*, suggesting that they adopted a more caudal identity following an additional 6 days in culture in the presence of retinoic acid (Fig. 5C, F). This caudal shift in identity from day 4 to day 10 was further supported by module scoring using the significantly differentially expressed genes between the NEP and NMP V2a neurons (Fig. 5G).

These data show that induced neurons possess a superficial similarity to V2a neurons. Upon close inspection, these cells lack co-expression of secondary marker genes (*SOX14* and *SOX21*) and express TFs that mark other cell populations (*PHOX2B* and *POU4F1*; Fig. 5C). Further, the axial identity of these neurons appears to be labile after achieving a morphologically “neuron-like” post-mitotic state (Fig. 5G). This contrasts with normal developmental progression, where neural progenitors fail to acquire more caudal identities after an early signaling window^20^. While we did not exhaustively test TF and small molecule combinations that could produce more robust V2a-like neurons, these experiments highlight that the most common V2a marker, VSX2, induced by LHX3 in a developmentally relevant progression is not sufficient to establish an underlying GRN that resembles that of a V2a neuron and that induced neurons are still susceptible to external cues. These findings prompted us to identify additional factors that govern the acquisition of lineally distinct V2a identities.

### GRN modeling predicts regulators of NMP-V2a neuron signature

**Fig. 6:**
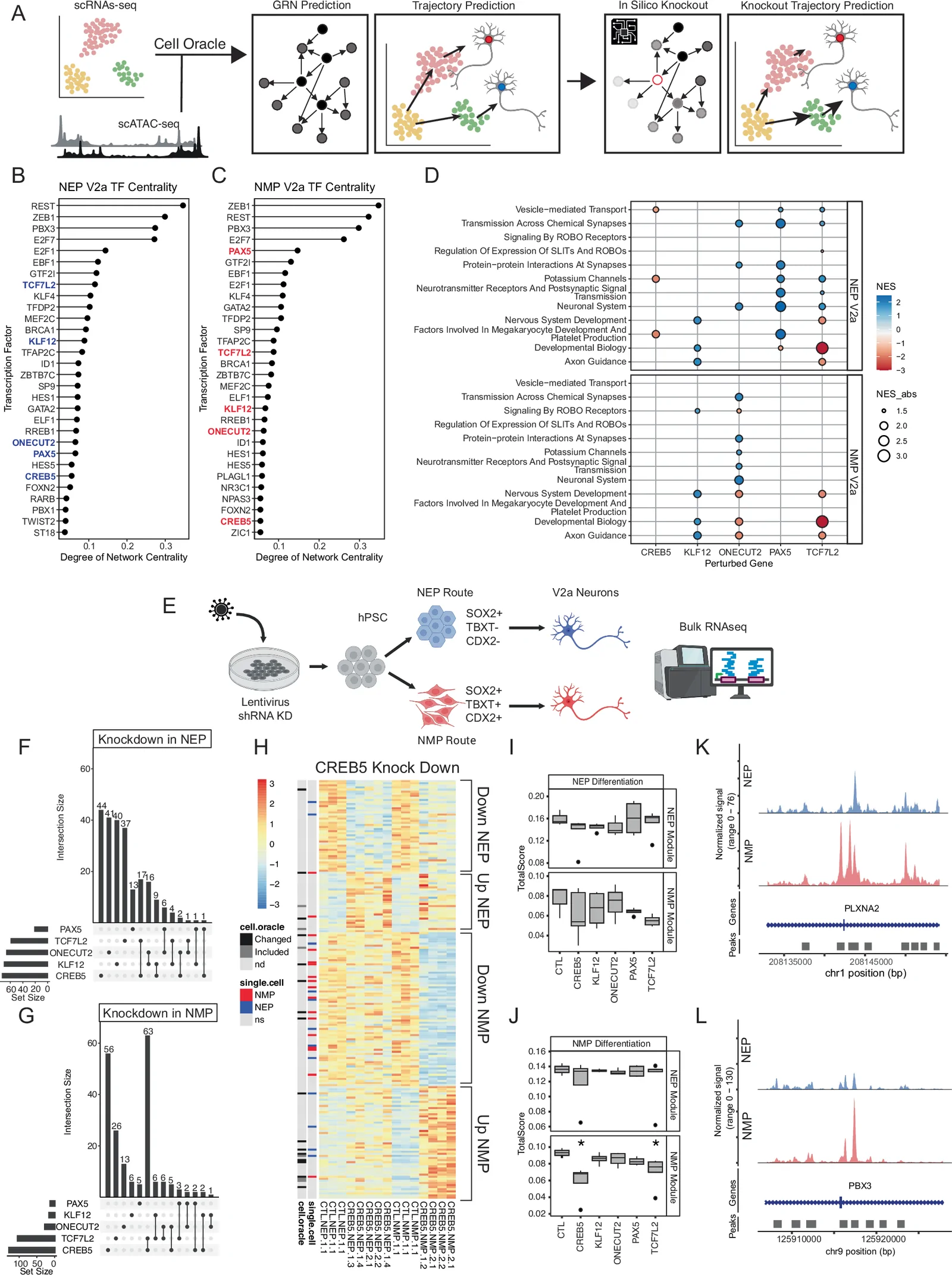
CREB5 and TCF7L2 regulate an NMP-enriched V2a gene signature. **A** Schematic of the CellOracle pipeline. Single-nucleus RNA- and ATAC-seq data are used to generate a predicted gene regulatory network (GRN). Specific nodes can be computationally knocked out and effects propagated through the network. Figure created with BioRender. https://BioRender.com/l10a099. **B**, **C** Top 30 transcription factors ranked by network centrality score in the predicted GRNs for NEP (**B**) and NMP (**C**) V2a neurons. **D** Gene set enrichment analysis (GSEA) normalized enrichment scores (NES) for selected gene sets in NEP and NMP V2a neurons after in silico gene perturbation. **E** Schematic of lentiviral knockdown (KD) validation experiments. Figure created with BioRender. https://BioRender.com/l10a099. **F** Upset plot of differentially expressed genes between control and KD of indicated genes under NEP differentiation conditions. **G** Upset plot of differentially expressed genes between control and KD of indicated genes under NMP differentiation conditions. **H** Heatmap of significantly differentially expressed genes in the CREB5 KD condition across NEP and NMP differentiations. **I** Single-sample GSEA scores in NEP condition knockdown samples using NEP and NMP V2a differentially expressed gene modules. Two-sided *t*-test. *n* = 3 for control and ONECUT2; *n* = 4 for KLF12 and PAX5; *n* = 5 for CREB5 and TCF7L2. Boxplot center line = median; bounds = first and third quartiles; whiskers = 1.5× the interquartile range; points = individual samples. **J** Single-sample GSEA scores in NMP condition knockdown samples using NEP and NMP V2a differentially expressed gene modules. *p = 0.047 (CREB5) and *p* = 0.048 (TCF7L2), two-sided *t*-test. *n* = 3 for control; *n* = 5 for TCF7L2; *n* = 4 for all other genes. Boxplot elements as in (**I**). **K**, **L** Coverage plots of NMP-enriched differentially accessible regions within PLXNA2 (**K**) and PBX3 (**L**), genes significantly downregulated in NMP CREB5 KD samples. Source data are provided as a Source Data file.

Having observed that different progenitor lineages conferred hundreds of differentially expressed genes and access to context-specific TF motifs in post-mitotic neurons, and that we could not closely recapitulate a V2a-like identity using an induced approach, we next sought to identify the GRN responsible for these differences. Rather than characterizing which TFs were differentially expressed in snRNA-seq or enriched in snATAC-seq data at the point of terminal differentiation, we wanted to understand the upstream regulatory logic that gave rise to these endpoint differences. For this, we turned to CellOracle, a computational tool which takes snRNA-seq and snATAC-seq data to construct population-specific GRNs based on correlating RNA expression and chromatin peak co-accessibility^46^. After building GRNs, individual TFs can be “knocked out” in silico in order to predict changes in cell state or differentiation trajectory (Fig. 6A).

Focusing on the V2a populations, we used CellOracle to produce separate GRNs for the NEP and NMP V2a populations using the top 3000 most variably expressed genes. We then examined which TFs contained the highest centrality scores in the predicted network, with higher scores correlating with larger numbers of total regulatory connections (Fig. 6B, C). Both V2a populations contained largely overlapping lists of highly central TFs, likely a result of their shared identity. To investigate which of these TFs may explain the observed condition-specific differentially expressed genes, we knocked down each of the top 25 most central genes in NEP-V2a or NMP-V2a populations in silico. We then performed Gene Set Enrichment Analysis (GSEA) on the perturbed conditions to identify what biological processes were up or downregulated upon knockout (Figs. 6D and S8). Several GSEA terms related to neural function were enriched in either the NEP-V2a or NMP-V2a condition by single gene knockout (Figs. 6D and S11). In order to experimentally validate these computational predictions, we narrowed down the list of potential targets using several criteria including expression level, whether or not the gene was differentially expressed in the RNA-seq data, whether the TF or similar TF motifs were enriched in the ATAC-seq data, and centrality in both conditions. We selected PAX5, CREB5, KLF12, ONECUT2, and TCF7L2 for further validation (highlighted in Fig. 6B–D). Since we hypothesize these proteins to be important in the lineage specific GRN, and because the specification of progenitors to either NEP or NMP lineage occurs within the first five days of differentiation, we knocked down these factors in pluripotent stem cells using short hairpin RNAs (shRNA) using lentiviral vectors. hPSCs were then differentiated along the NEP- or NMP-derived routes toward V2a neurons (Fig. 6E) and bulk RNA was collected for sequencing on day 19.

Next, we examined the genes that were differentially expressed in NEP- or NMP-derived knockdown conditions (Figs. 6F, G and S12A–D). Contrasting with the NEP-derived condition where the majority of differentially expressed genes (DEG) were specific to knockdown of one gene (Fig. 6F), we noticed that knockdown of either *CREB5* or *TCF7L2* in the NMP-derived condition resulted in the largest total numbers of DEG, many of which were overlapping (Fig. 6G). A heatmap of the differentially expressed genes in the *CREB5* knockdowns showed that the NEP-V2a specific DEG exhibited similar trends in the NMP conditions, although not statistically significant (i.e., genes that went down in the NEP-derived KD condition also decreased in the NMP-derived KD condition, though to a lesser degree; Fig. 6H). The NMP-specific DEG, however, had highly specific effects, and were also enriched for the DEG identified in our multiome data (Fig. 6H). Similar observations were made for the *TCF7L2* knockdown (Fig. S12A). While neither the NEP or NMP module was significantly altered in the NEP differentiations, both *CREB5* and *TCF7L2* knockdowns significantly reduced the NMP module in NMP differentiations (Fig. 6I, J). Thus, it appears that CREB5 and TCF7L2 play key regulatory roles specific to the NMP lineage and that they contain largely overlapping regulons.

Lastly, we examined the list of differentially accessible regions between NEP and NMP V2a neurons and filtered it for peaks that were annotated in or nearest to the DEG in the NMP CREB5 knockdown samples. This identified 25 NMP-enriched peaks within or near 15 unique genes downregulated by *CREB5* KD, including *PLXNA2*, a semaphorin receptor which plays a role in axon guidance, and *PBX3*, a TF which can form complexes with HOX genes and is thought to play a wide role in early development (Fig. 6K, L). These findings suggest that CREB5 and TCF7L2 could regulate some of their DEG by either opening unique regions of chromatin or by binding to these peaks to regulate transcription.

## Discussion

Our study demonstrates that distinct developmental trajectories in the differentiation of V2a neurons result in lasting, lineage-specific transcriptional and functional differences. Using two distinct progenitor differentiation methods—one generating neuroectodermal progenitors (NEP) and the other NMP—we observed that these lineages exhibit differential gene expression, chromatin accessibility, and network activity, even under identical culture conditions during the later stages of differentiation.

An interesting observation from the V2a differentiations was the consistently higher proportion of V2a neurons derived from the caudal NMP progenitors. We speculate that this is due to an interaction of the effect of WNT and SHH signaling. After neural tube closure, WNT and SHH signals are known to antagonize one another to establish the dorsal and ventral neuron domains. This is at least partially accomplished via WNT-induced expression of GLI3, a SHH repressor^47,48^. Further, there is evidence that WNT effector beta-catenin can bind and sequester the SHH activator GLI1^49^. These potential interactions point to several routes by which induction of WNT signaling during NMP specification may dampen the ability of the NMP-derived neural progenitors to respond to the SHH agonism during V2a differentiation. Together, these would result in a slightly more dorsal-biased population of neurons in the NMP-derived condition and a relatively ventral-biased population of neurons in the NEP-derived condition, as we observed. Importantly, we do not claim that NEP-derived progenitors are innately less able to produce V2a neurons than NMP-derived progenitors, simply that there was a bias under the uniform differentiation condition tested. This interaction of WNT and SHH signaling may also explain why the FP population was exclusively present in the NEP-derived population, since SHH signaling from the notochord is necessary to specify FP cells in vivo. Hence, the presence of FP cells could both exemplify and exacerbate a ventral bias in NEP-derived cultures via their production of SHH ligands.

Our ATAC-seq analysis revealed that early lineage differences result in unique sets of accessible chromatin at the mature neuron stage, suggesting that early developmental events influence long-term gene regulation in V2a neurons. The NMP-V2a specific peaks, for example, displayed enrichment in TF motifs associated with homeodomain-containing TFs, which are critical for regionalization and spinal cord identity. Conversely, NEP-V2a specific peaks showed a broader distribution of bHLH and Rel homology motifs, consistent with a hindbrain-like identity. Comparison of either NEP- or NMP-enriched peaks across p2 and V2a stages revealed overlap of only 7.8 and 13.2%, respectively, at the level of individual peaks, yet motif analysis of the peaks enriched in the same condition at each stage identified overlapping TF families (Fig. S7A–D). Differences in accessible chromatin over the course of differentiation or between closely related populations has been described in various other CNS models^50–52^. Our observations further underscore the impact of progenitor origin on the epigenetic landscape and how it can affect differentiation outcomes within a single neuron subtype.

Identification of the precise mechanism by which changes in progenitors are passed forward to mature neurons requires future time-resolved datasets of ATAC-seq and ChIP-seq data. However, several mechanisms could explain how shared TF families can act through distinct regions of open chromatin between the two V2a populations. First, pioneer TFs, such as CDX2 and ASCL1, have been shown to remove repressive histone modifications and directly bind new regions of chromatin^41,53^. Expression of a pioneer factor in a single lineage could open unique regions of chromatin. Further, TF activity has been well described to be modulated by the presence of cofactors, which allow for tissue-specific activation, as in the case of PBX and MEIS for HOX genes and ISL1 in the context of LHX3^42,54^. Lastly, looking at the genome only through the lens of ATAC-seq limited our analysis to open and actively accessible chromatin. Specific chromatin modifications, like H3K4me1, can label poised enhancers which would be invisible to ATAC-seq and can be written at different enhancer subsets during differentiation^55^.

Building on our characterization of chromatin differences, we identified TFs that influenced the NEP or NMP-like transcriptional signatures. Our computational analysis and knockdown studies identified CREB5 and TCF7L2 as potential regulators of NMP-specific gene networks in V2a neurons, warranting further investigation into their role in modulating neurodevelopmental pathways. Since knockdown was constitutive and began at the stem cell state, further studies are necessary to determine the developmental window in which knockdown is necessary to produce transcriptional differences and to determine the functional impact on V2a neurons. Involvement of TCF7L2 and possibly CREB5 in WNT signaling and developmental regulation may explain why these genes produced NMP-lineage specific effects and supports a role for them in the early lineage specification process^56–58^. These insights shed light onto the processes and regulatory networks at play during differentiation, how they are carried forward through development, and may aid in the development of improved differentiation protocols for generating functionally specialized V2a neurons.

In functional assays, we matured neuron cultures for up to 24 additional days and observed spontaneous activity using calcium imaging. These experiments showed that NMP-derived neuron cultures displayed more sustained synchronous network activity compared to their NEP counterparts. Both in vivo and in vitro studies point to glutamatergic neurons, like V2a interneurons, as being a major driver of patterned or bursting activity^37–39^. It is possible that the transcriptional differences between NEP- and NMP-derived V2a neurons surfaced above result in intrinsic differences in synaptic sensitivity and strength that account for the observed activity differences and which could influence their suitability in regenerative therapies targeting motor circuits. However, this conclusion is confounded by the presence of MNs and other interneurons, which could also alter excitatory/inhibitory ratio and network activity. Future studies with more balanced populations or purified populations are required to determine which properties of V2a neurons are intrinsically altered by their developmental lineage. Future functional studies should also explore whether bursting NMP-V2a interneurons also prompt strong bursting MN activity specifically, as well as circuit integration and functional synaptic integration post-transplantation.

Our experiments with induced NGN2-LHX3 neurons, which bypassed key developmental patterning stages, yielded cells that displayed some superficial characteristics of V2a neurons but differed significantly in transcriptional identity. The dissimilarity between these induced neurons and the NEP- and NMP-derived V2a neurons highlights the importance of developmental patterning to the terminal cell identity and suggests that even the core V2a TF VSX2 is not sufficient to confer a V2a identity when development is bypassed. The axial identity of these induced neurons remained labile, indicating that essential developmental cues are necessary to establish and maintain regional specificity. These findings are consistent with previous studies showing that induced neurons often lack the stable chromatin modifications characteristic of lineage-specific differentiation. Our results show that induced neurons do not fully replicate the GRNs of authentic V2a neurons, limiting their applicability in disease modeling and therapeutic discovery where functional specificity is critical. Future studies could screen additional TFs or extraneous cues to enhance the fidelity of induced V2a neurons and provide a more nuanced understanding of their development and core GRN. By pinpointing key regulators that drive lineage-specific traits, this approach could provide insights into constructing more stable and functionally accurate V2a neuron populations, ultimately improving their utility in disease models and therapeutic applications.

In summary, our study establishes a foundational framework that extends beyond the specific context of V2a neurons, underscoring the importance of refining hPSC differentiation strategies. Our findings emphasize the necessity of generating cells that not only express key lineage markers but also faithfully replicate the developmental stages and processes encountered during fetal development. Enhancing the specificity and accuracy of differentiations will enable more precise and diverse basic and translational applications.

### Limitations of the study

While our study design allows us to isolate the effects of NEP or NMP progenitor lineage on the V2a neurons, our multiomic analysis of the differences between these lineages only captured a single time point. While we do capture some population heterogeneity and are able to separate out p2 populations for some pseudotemporal analysis, we cannot make concrete claims about whether the lineage identity is retained from early to late time points. Further, our use of snATAC-seq allows us to see which motifs are enriched in specific populations, but ChIP-seq or similar experiments would be needed to identify TF binding at specific loci. Our observations of different patterns of activity between maturing NEP and NMP cultures, while striking, are confounded by mixed populations. Different proportions of motor and other interneuron populations could also influence the degree and frequency of burst activity and targeted drug perturbations or sorted cultures would be needed to isolate these effects. While we identified some DEG between NEP and NMP V2a neurons that could influence activity and synaptic transmission, they were also not directly linked to the observed differences in function. Sequencing at later time points could point to whether these differences were sustained or if other DEG appear with the functional differences.

## Methods

### Stem cell maintenance

The parental stem cell lines used in this study include H9 (WiCell WA09; female), WTC11 (Coriell Institute GM25256; male), and WTB lines (Conklin Lab; Miyaoka paper^59^; female). Stem cells were seeded on Geltrex coated plates and maintained in mTeSR1 with daily feeds or in mTeSR Plus and fed every other day and kept at 37 °C and 5% CO_2_. Stem cells were passaged at ~70% confluency using Accutase at a 1:10 ratio. A defined pro-survival small molecule cocktail, CET, was included in the media for the first 24 h after passaging^60^. All stem cell lines were tested quarterly for mycoplasma.

### Neuromesodermal progenitor differentiation

Neuromesodermal progenitor (NMP) differentiation loosely followed the protocol set out in ref. ^32^. Briefly, stem cells were passaged as normal in 6-well plates and allowed to grow to ~70% confluency in mTeSR Plus. To begin differentiation, cells were then fed with 4 mL/well E6 media. One day later, cells were fed with 4 mL/well E6 with 20 ng/mL bFGF. Following 24-h of FGF treatment, at which point cells were 100% confluent, cells were washed 1× with PBS and dissociated using 1.5 mL/well of Accutase in the incubator for ~10 min. Dissociated cells were collected, washed, and spun at 350 × *g* for 2 min. For each well that was dissociated, cells were resuspended in 12 mL of E6 media containing 1× CET, 10  μM SB 431542 (SB), 20 ng/mL bFGF, and 3–5 μM CHIR99021 (CHIR). Note, the CHIR dose should be adjusted for each cell line; WTC-11 and derivative cell lines require 3 uM CHIR, while WTB and H9 require 5 μM CHIR. Cells were plated into Geltrex-coated 24-well plates using 0.8 mL of cell suspension per well. Forty-eight hours later, cells had a cobble-stone appearance covering 100% of the well, and were either collected to assess NMP identity or immediately transitioned into day 5 differentiation media (see V2a differentiation below).

### V2a differentiation

The NEP V2a differentiation followed the detailed protocol outlined in ref. ^31^ with minor modifications. To start differentiation (day 0), stem cell cultures at ~70% confluency were dissociated using Accutase, counted, and resuspended in mTeSR Plus with 1× CET, 10 μM SB, and 200 nM LDN 193189 (LDN) to a concentration of 100 k cells/mL. 1 mL of cell suspension was plated into each well of Geltrex-coated 24-well plates, resulting in a final seeding density of ~50 k cells/cm^2^. At days 1 and 3, cells were fed with 2 mL/well mTeSR Plus with 10 μM SB and 200 nM LDN. By day 5, cells were 100% confluent. From day 5 onward, the base media was changed to Neural Induction Media (NIM; DMEM:F12 containing 1× N2 supplement, 1× Gluta-gro, 1× NEAA, 1× PenStrep, 0.4 μg/mL ascorbic acid, and 2 μg/mL heparin). On day 5, cells were fed with 2 mL/well NIM with 100 nM retinoic acid, 10 ng/mL BDNF, 10 μM SB, and 200 nM LDN. Day 5 also marks the time point at which NMPs are transitioned into the neural differentiation. From day 7 to 19, cells were fed every other day with 2 mL/well of NIM with 100 nM retinoic acid, 10 ng/mL BDNF, 0.4 μg/mL ascorbic acid, 300 nM PUR, and 1 μM DAPT.

### V2a dissociation and cryopreservation

On day 19 of neuron differentiation, cultures were washed 1× with PBS, then incubated for 20 min with 1 mL/well of Accutase. An additional 1 mL of PBS was then added to each well and neural cultures were broken up by smoothly pipetting up and down 10×. Dissociated cells were then pooled in a vessel containing an additional 24 mL of PBS (or an additional mL of PBS for every well being dissociated). Dissociated cells were distributed to 50 mL conicals and centrifuged at 300 × *g* for 3 min and supernatant was aspirated, being careful not to disturb the cell pellet. Cells were resuspended in 5–8 mL of NIM with 100 nM RA, 10 ng/mL BDNF, 300 nM PUR, 1 μM DAPT, and 1× CET. The cell suspension was passed through a 37 μm filter and counted using an automated cell counter and Trypan dye. Each well of a 24-well plate should yield 3–4e6 cells. The cell concentration was then adjusted to ~8–10e6 cells/mL by adding additional supplemented NIM. Cells were then mixed in a 1:1 ratio with cell preservation media (80% FBS, 20% DMSO) and distributed into labeled cryovials. Prior to transfer to LN2 for long-term storage, cryovials were chilled in a −80 °C freezer in CoolCell (Corning) or Mr. Frosty (Thermo Fisher) containers to keep the cooling rate to −1 °C per minute.

### Flow cytometry and FACS

Cells were dissociated as described above. Cells were spun for 2 min at 350 × *g*, then supernatant was aspirated and cells resuspended in 100 μL of PBS. Single cell suspensions were fixed using the FOXP3/TF kit (eBiosciences) following the manufacturer’s protocol. Cells in the Perm Buffer were stored at 4 °C until use. For staining, the desired volume of cells was transferred to a clean 1.5 mL tube and washed 1× with 500 μL of PBS containing 0.1% BSA. Cells were spun in a fixed-angle centrifuge at 400 × *g* for 1.5 min, then tubes were rotated 180° and spun again. Supernatant was aspirated leaving ~25 μL of liquid in the bottom of the tube to not disturb the cell pellet. Primary and secondary antibodies were diluted in FOXP3 kit Perm Buffer and cells were stained in 100 μL volume in the dark at room temperature for 1 h or overnight at 4 °C. Cells were washed 2× with PBS/BSA between the primary and secondary antibody steps. For antibody product numbers and dilutions, see Supplementary Data 4. All flow cytometry was carried out on an Attune NxT. A representative gating strategy for cytometry experiments can be found in the Supplemental Note Fig. S15. For live cell sorting, dissociated cells were resuspended in DMEM/F12 with 0.5 uM EDTA and 1× CET to a concentration of ~10e6 cells/mL. Cells were immediately sorted on a FACS Aria Fusion into the same media.

### Immunostaining

For staining cells in plates, cells were fixed with 4% PFA at room temperature for 10 min, then washed 3× with PBS. Cells were blocked for 1 h at room temperature in PBS containing 5% normal donkey serum and 0.3% Triton X-100. Primary and secondary antibodies were diluted in PBS containing 1% BSA and 0.3% Triton X-100 and incubated at room temperature for 1 h or overnight at 4 °C, with 3× washes of PBS following each antibody incubation. For antibody product numbers and dilutions, see Supplementary Data 4.

### Nuclei Isolation for 10× Multiome

For multiome, all samples had been previously cryopreserved. Samples were thawed, washed with 10 mL of NIM, resuspended in 0.5 mL of NIM with CET and counted using Trypan Blue dye to assess viability. For sample pooling, 333 k cells from three technical replicates were pooled, and 500 k cells were used for subsequent nuclei isolation. Nuclei isolation followed 10× Genomics Demonstrated Protocol CG000365 Rev C: Nuclei Isolation for Single Cell Multiome ATAC + Gene Expression Sequencing including the DNase treatment step and using 0.8× lysis buffer strength. Nuclei were submitted to the UCSF Genomics CoLabs for library preparation and sequenced by the UCSF Institute for Human Genetics.

### Multiome data analysis

For each sample, multiome sequencing data was aligned to the Human GRCh38 reference genome (10× Genomics) containing custom reference sequences for integrated transgenes using CellRanger ARC (v2.0.2). Background RNA transcripts were then removed using CellBender (v0.1) before creation of a dual RNA/ATAC object and data analysis using Seurat (v4.4.0) and (Signac v1.9.0). Cells were filtered to have between 500 and 10,000 detected genes, between 1000 and 50,000 RNA UMI, TSS enrichment score greater than 1, a nucleosome signal less than 2, and at least 1000 ATAC UMI. DoubletFinder (v2.0.3) was implemented only on RNA data to detect and remove presumptive doublets. RNA assay data was log normalized and scaled, while ATAC assay data was processed by latent semantic indexing using the RunTFIDF() and RunSVD() functions. Both assays were incorporated for UMAP visualization using the FindMultimodalNeighbors() function, and clustering was performed on RNA data with FindClusters() using the Louvain algorithm with multilevel refinement (algorithm = 2) and a resolution of 0.8. Clusters were annotated as described in the text by examining relative expression levels of marker genes. To facilitate balanced analysis, samples from matched cell lines (i.e., NEP-Rep1 and NMP-Rep1) were subset to approximately the same number of cells. Subsetting included taking a random sample of a fixed number of cells as well as all cells in p2 and V2a clusters, since they represented the major cell types of interest. Only after each sample was individually annotated, and subset were samples merged using Harmony integration (theta = 1, sigma = 0.6). 12,020 cells were included in the subset and integrated object (NEP, NMP, and IND samples). An alternative approach of combining samples using Harmony integration prior to clustering and cell annotation produced highly similar results (data not shown). Samples NEP-Rep1 and NMP-Rep1, generated from the WTC11 cell line, were found to have lower than expected overall cell numbers. Seeing as all the samples were normalized to the same starting cell number and had similar viability upon starting sample prep, this discrepancy likely arose during the 10× loading process, but the root cause is unknown.

In the merged data, differentially expressed genes were identified using the FindMarkers() function using the Wilcoxon test (test.use = “wilcox”). Differentially expressed genes were tested for enriched gene ontology terms using the enrichGO() function in the clusterProfiler (v4.4.4) package. Differentially accessible ATAC peaks were identified using FindMarkers() function (test.use = “LR” and min.pct = 0.1). The genes near the differentially accessible peaks were tested for enriched gene ontology terms using the GREAT tool (http://great.stanford.edu/public/html/). Motifs were assigned to ATAC peaks with the AddMotifs() function and using the JASPAR2020 motif database. Motif enrichment in ATAC regions was run using the FindMotifs() function and compared to a set of 50,000 GC-content matched background peaks identified using MatchRegionStats(). For label transfer, human fetal samples were downloaded from GEO (GSE171890 [https://www.ncbi.nlm.nih.gov/geo/query/acc.cgi?acc= GSE171890]) with the provided cell annotations. Each sample was subset to ventral spinal cord cell types then integrated into a single object in Seurat. NEP and NMP in vitro datasets were mapped onto the in vivo data using FindTransferAnchors() and TransferData() functions.

### Calcium imaging

Freshly thawed d19 neurons were counted and replated at a density of 150,000 cells per well into geltrex coated 96-well plates in NIM supplemented with 100 nM retinoic acid, 10 ng/mL BDNF, 0.4 μg/mL ascorbic acid, 300 nM PUR, 1 μM DAPT, and 1× CET cocktail. After two days, an additional 1× volume supplemented BrainPhys media was added to each well. BrainPhys media (StemCell Technologies) was supplemented with 1:50 SM1 supplement, 1:100 CultureOne supplement, 1× PenStrep, 10 ng/mL BDNF, and 10 ng/mL GDNF. At indicated time points, media was fully removed and replaced with BrainPhys Imaging Optimized Medium with 1 μg/mL Fluo4-AM (Ion Biosciences) and 1:100 Pluronic F-127 (Ion Biosciences) and incubated for 1 h. Media was then again completely replaced with warmed BrainPhys Imaging Optimized Medium alone and cells were imaged on an ImageXpress Confocal HT (Molecular Devices) equipped with stage heating and CO_2_ control (37 °C, 5% CO_2_) at 10× magnification for 1 min at a frame rate of 2 Hz. After calcium imaging, cells were fed with supplemented BrainPhys media and returned to the incubator for continued maturation.

Calcium imaging data was analyzed using custom written Python code. Dense cultures and overlapping cells prevented easy masking on cell bodies for ROI, so a more agnostic approach was taken. All images in a single imaging session were collapsed into a maximum projection and then a global threshold was applied to create a mask of the recorded cell area. The cell mask was then divided into a 64 × 64 grid, with each square of the grid being treated as a single ROI (4096 potential ROI). All pixels within the ROI which were included in the threshold mask were included in fluorescent intensity calculations. ROI with no pixels included in the threshold mask were not quantified.

### Induced neuron cell line creation

The NGN2:LHX3 induced cell line was produced by modifying an existing plasmid for induced MNs: PB-TO-hNIL was a gift from iPSC Neurodegenerative Disease Initiative & Michael Ward (Addgene plasmid # 172113). PB-TO-hNIL was amplified in a PCR reaction to remove the ISL1 coding sequence using the forward primer ATGGAAGCCAGAGGCGAAC and reverse primer AGGTCCAGGGTTCTCCTCCACGTCTC using KOD Xtreme^TM^ Hotstart Polymerase (Millipore), then digested with DpnI (NEB) and recircularized using a T4 ligase (NEB) blunt end ligation with PNK (NEB). The resulting plasmid was transformed into DH5ɑ competent *E. coli* (NEB) for colony selection and miniprep purification using a QIAprep Spin Miniprep Kit (QIAGEN). Deletion of ISL1 was confirmed by separate restriction digests with DraIII, SmaI, and BbsI. For transfection, WTC11 stem cells were passaged at a density of 25 k cells/well into a 24-well plate. The following day, the cells were fed with fresh media for 1 h prior to transfection using 250 ng each of the NGN2:LHX3 plasmid and pEIF1a::Transposase (gifted by Dr. M. Ward), and 1 μL of Lipofectamine Stem (Invitrogen) prepared according to manufacturer’s instructions. The following day, the entire population of cells was passaged into T25 flasks and allowed to grow for another 24 h prior to selection with 0.5 μg/mL puromycin. Purified cells were expanded and cryopreserved until future use.

### Induced neuron differentiation

Induced neuron differentiation protocols were based on previously described protocols for NGN2 and NGN2:ISL1:LHX3 neurons^5^. For induced neuron differentiation, stem cells were grown to near confluency on Geltrex-coated plates before changing to induction media (NIM supplemented with 1 μM DAPT, 2 μg/mL doxycycline (dox), and listed concentrations of RA (most efficient for NGN2:LHX3 line is 1 μM RA). Cells were then fed every other day. On day 2, cells received NIM with 2 μg/mL dox, RA, and 10 ng/mL BDNF. From day 4 onward, the media formulation was the same as day 2, but without dox.

### RNA isolation and qPCR

RNA was isolated using the QIAGEN RNeasy Mini Plus Kit according to manufacturer’s instructions. Whenever possible, cells were lysed directly in the culture vessel. Note, for highly dense V2a differentiations from day 9–19, up to 1 mL of RLT with BME was used per well of a 24-well plate. Cell lysate was transferred to 1.5 mL tubes and directly placed in a −80 °C freezer until RNA extraction. QIAShredder tubes were used to homogenize samples prior to gDNA clean-up and RNA isolation. RNA was eluted in 50 μL of nuclease-free water (Ambion) and quantified via nanodrop. If samples had insufficient concentration (< 60 ng/μL) or salt contaminants (260/230 ratios <1.5), they were further processed using the Zymo RNA Clean and Concentrator Kit. If not used immediately, RNA was stored at −80 °C. RNA was converted to cDNA for qPCR using the qScript cDNA SuperMix (Quanta Bio) according to manufacturer’s instructions. qPCR reactions were set up in 10 μL volumes in 384-well plates using 20 ng of cDNA per reaction, 2× PowerUp SYBR Green Master Mix, and 200 nM of each primer. Custom primer sequences were ordered from IDT as single-stranded DNA oligonucleotides. qPCR reactions were run on a QuantStudio 6 Flex following the manufacturer recommended standard cycling conditions for the PowerUp SYBR Green Master Mix.

### Cell oracle

Cells were filtered to include only those with scATAC-seq peaks between 2000 and 20,000. Cicero was used to extract cis-regulatory connections and identify co-accessible peaks^61^. We scanned these peaks for TF motifs using the motif scan function from CellOracle^46^.

For the scRNA-seq data, we followed similar preprocessing steps to analyses from previous section, and then filtered down cells to p2 and V2a annotated cells. The remaining clusters were then integrated based on their NMP/NEP identity using the Combat function in Scanpy^62^. Genes were filtered to include the top 3000 most variable genes. The base GRN was generated through the CellOracle pipeline by combining ATAC-seq peaks with motif information, after which centrality scores for each node in the GRN were calculated. Pseudotime analysis was performed based on NEP and NMP lineages.

Desired perturbations were simulated using the generated GRNs and the CellOracle pipeline. Subsequently, the gradient of shifts and effects on developmental pseudotime were calculated. Pre- and post-perturbation gene expression matrices were exported, and gene set enrichment analysis was performed using the GSEApy package with the c2.cp.reactome category (database version 2023.2.Hs) from the Msigdb class^63^. Enrichment was analyzed with the gp.gsea function, utilizing the signal-to-noise ratio metric for gene ranking and permutation testing on pre- and post-perturbation data.

### Lentiviral shRNA production and knockdown

Cloning and lentivirus production followed recommended protocols by the Broad Institute Genetic Perturbation Platform (https://portals.broadinstitute.org/gpp/public/). Briefly, the pLKO.1 cloning vector (a gift from David Root; Addgene plasmid # 10878) was digested with AgeI and EcoRI and gel-purified. Oligos containing shRNA sequences against target genes (see Supplementary Data 5; IDT) were annealed to form double stranded inserts and ligated into the pLKO.1 backbone. Two shRNA different sequences per target gene were made, for a total of 10 unique plasmids. Plasmid DNA was isolated using ZymoPURE Plasmid Miniprep Kit (Zymo) and confirmed using long-read whole plasmid sequencing (Plasmidsaurus).

For lentivirus production, pLKO.1-shRNA plasmid, pCMV-dR8.91, and pMD2.G (a gift from Didier Trono; Addgene plasmid # 12259) plasmids were combined in a 9:8:1 ratio and transfected into HEK293T cells using Nanofect Transfection reagent (ALSTEM) following the manufacturer’s protocol. Media was changed the following day and collected at 48 and 72 h post-transfection. Viral supernatant was stored at −20 °C until use. For WTC11 stem cell transduction, viral supernatant was added to stem cell media in a 1:20 ratio with 10 μg/mL polybrene (Millipore). After two days, stem cells were selected using 0.5 μg/mL puromycin for an additional two days before subsequent expansion. Upon differentiation to V2a neurons by NEP and NMP routes, cultures were additionally selected with puromycin between days 7 and 9 to ensure that the shRNA construct had not been silenced.

### Bulk RNA sequencing and analysis

Bulk RNA sequencing libraries were produced using the Mercurious™ High-sensitivity BRB-seq kit (Alithea Genomics). For the high-sensitivity BRB-seq kit, purified RNA was first isolated using the RNeasy Mini Plus kit (QIAGEN). Libraries were sequenced on a NovaSeq X (Illumina) by the UCSF CAT Core. Code for sequence quality control, alignment, and generation of count matrices followed MERCURIUS^TM^ High sensitivity BRB-seq kit recommendations and used fastQC (v0.11.5) and STAR (v2.7.11b) packages. Samples with greater than 2e6 total counts were kept for further analysis. Counts and metadata tables were combined into a single dataset using DESeq2 (v1.36.0). For all-sample comparison using PCA, VST-normalized counts were extracted and batch corrected using the limma (v3.54.2) removeBatchEffect() function. For heatmap plotting, the size-factor normalized counts were extracted and used. For differential expression testing, the counts matrices were subset to only include either NEP or NMP data before generating the DESeq object using a “~ batch + KD_Gene” design. The lists of differentially expressed genes were filtered to only include genes that crossed the significance threshold in 1 or 2 samples. For single-sample GSEA scoring, raw counts matrices were TPM normalized prior to running removeBatchEffect(). This corrected TPM matrix was then ranked and analyzed using SingScore (v1.19.1) with the differentially expressed genes between NEP and NMP V2a neurons from multiome being used as input gene sets.

### Reporting summary

Further information on research design is available in the Nature Portfolio Reporting Summary linked to this article.

## Supplementary information


Supplementary Information
Peer Review file
Description of Additional Supplementary Files
Supplementary Data 1
Supplementary Data 2
Supplementary Data 3
Supplementary Data 4
Supplementary Data 5
Supplementary Movie 1
Supplementary Movie 2
Supplementary Movie 3
Supplementary Movie 4
Reporting Summary


## Source data


Source Data


## Data Availability

Data supporting findings of this study are available within the article and its Supplementary Information and Source Data files. The sequencing data generated in this study (snRNA-seq, snATAC-seq, and bulk RNA-seq for knockdown studies) have been deposited in the Gene Expression Omnibus under accession number GSE283005. Source data are provided with this paper.

## Code availability

Analysis of sequencing data utilized publicly available tools and packages as referenced in the “Methods” section. CellOracle analysis scripts are available at [https://github.com/Alireza-Majd/celloracle_pipeline]. Custom R, Python, and command-line scripts used to analyze calcium imaging data are available from the authors upon request.
