# Supplementary material for: Distinct differentiation trajectories leave lasting impacts on gene regulation and function of V2a neurons
Source: Nat Commun. 2026 Jul 29;17:9187. doi: 10.1038/s41467-026-76054-w (PMC13522347; doi:10.1038/s41467-026-76054-w)
Supplement: Supplementary file 1 [file 41467_2026_76054_MOESM13_ESM.pdf]

nature portfolio

Corresponding author(s): Faranak Fattahi

Last updated by author(s): Jun 27, 2026

Reporting Summary

Nature Portfolio wishes to improve the reproducibility of the work that we publish. This form provides structure for consistency and transparency in reporting. For further information on Nature Portfolio policies, see our Editorial Policies and the Editorial Policy Checklist.

Statistics

For all statistical analyses, confirm that the following items are present in the figure legend, table legend, main text, or Methods section.

n/a	Confirmed	
☐	☒	The exact sample size (*n*) for each experimental group/condition, given as a discrete number and unit of measurement
☐	☒	A statement on whether measurements were taken from distinct samples or whether the same sample was measured repeatedly
☐	☒	The statistical test(s) used AND whether they are one- or two-sided<br>*Only common tests should be described solely by name; describe more complex techniques in the Methods section.*
☒	☐	A description of all covariates tested
☐	☒	A description of any assumptions or corrections, such as tests of normality and adjustment for multiple comparisons
☐	☒	A full description of the statistical parameters including central tendency (e.g. means) or other basic estimates (e.g. regression coefficient) AND variation (e.g. standard deviation) or associated estimates of uncertainty (e.g. confidence intervals)
☐	☒	For null hypothesis testing, the test statistic (e.g. *F*, *t*, *r*) with confidence intervals, effect sizes, degrees of freedom and *P* value noted<br>*Give P values as exact values whenever suitable.*
☒	☐	For Bayesian analysis, information on the choice of priors and Markov chain Monte Carlo settings
☐	☒	For hierarchical and complex designs, identification of the appropriate level for tests and full reporting of outcomes
☒	☐	Estimates of effect sizes (e.g. Cohen's *d*, Pearson's *r*), indicating how they were calculated

Our web collection on statistics for biologists contains articles on many of the points above.

Software and code

Policy information about availability of computer code

Data collection	Multiome samples were prepared using the Chromium Next GEM Single Cell Multiome ATAC + Gene Expression kit (10X Genomics) following manufacturer approved protocols. Bulk RNA samples were produced using Mercurious High sensitivity BRB-seq kit (Alithea Genomics). Libraries were sequenced on an Illumina NovaSeq sequencer by the UCSF Genomics CoLabs and Center for Advanced Technology, respectively. Genome alignment utilized CellRangerARC v2.0.2, fastQC v0.11.5, and STAR v2.7.11b tools.
Data analysis	Data analysis used open access code and packages, including R v4.3.0, CellBender v0.1, Seurat v4.4, Signac v1.9, DoubletFinder v2.0.3, clusterProfiler v4.4.4, DESeq2 v1.26.0, limma v3.54.2, singscore v1.19.1, TFBSTools v3.20, JASPAR2020 v0.99.10, Python v3.11, CellOracle v0.10.12. Analysis and data visualization also used Adobe Illustrator, Microsoft Excel, MolecularDevices MetaXpress, and FlowJo software. Custom scripts were written for calcium imaging data analysis and are avaialable upon request.

For manuscripts utilizing custom algorithms or software that are central to the research but not yet described in published literature, software must be made available to editors and reviewers. We strongly encourage code deposition in a community repository (e.g. GitHub). See the Nature Portfolio guidelines for submitting code & software for further information.

Data

Policy information about availability of data

All manuscripts must include a data availability statement. This statement should provide the following information, where applicable:

- Accession codes, unique identifiers, or web links for publicly available datasets
- A description of any restrictions on data availability
- For clinical datasets or third party data, please ensure that the statement adheres to our policy

Data supporting findings of this study are available within the article and its Supplementary Information and Source Data files. The sequencing data generated in this study (snRNA-seq, snATAC-seq, and bulk RNA seq for knockdown studies) have been deposited in the Gene Expression Omnibus under accession number GSE283005 [https://www.ncbi.nlm.nih.gov/geo/query/acc.cgi?acc=GSE283005]. Source data are provided with this paper.

Analysis of sequencing data utilized publicly available tools and packages as referenced in the methods section. CellOracle analysis scripts are available at [https://github.com/Alireza-Majd/CellOracle]. Custom R, Python, and command line scripts used to analyze calcium imaging data are available from the authors upon request.

Research involving human participants, their data, or biological material

Policy information about studies with human participants or human data. See also policy information about sex, gender (identity/presentation), and sexual orientation and race, ethnicity and racism.

Reporting on sex and gender	N/A
Reporting on race, ethnicity, or other socially relevant groupings	N/A
Population characteristics	N/A
Recruitment	N/A
Ethics oversight	N/A

Note that full information on the approval of the study protocol must also be provided in the manuscript.

Field-specific reporting

Please select the one below that is the best fit for your research. If you are not sure, read the appropriate sections before making your selection.

[x] Life sciences [ ] Behavioural & social sciences [ ] Ecological, evolutionary & environmental sciences

For a reference copy of the document with all sections, see nature.com/documents/nr-reporting-summary-flat.pdf

Life sciences study design

All studies must disclose on these points even when the disclosure is negative.

Sample size	A minimum sample size of n=3 was selected based on convention in the field. When resources allowed, multiple technical replicates were included for each biological replicate to increase confidence in the reproducibility.
Data exclusions	No data that otherwise passed quality control exclusion criteria was excluded from analysis.
Replication	Wherever possible, experiments were replicated in three biologically independent cell lines or a minimum of n=3 technical replicates coming from independent and separate differentiations separated by at least 1 passage.
Randomization	Randomization was not used in this study. However, cells from the same source were used to start both NEP and NMP differentiations when possible.
Blinding	Blinding was not used in this study. For sequencing experiments, samples were not blinded because their identity was inherent to the experimental condition being tested. For cytometry, samples being compared were stained with the same primary and secondary antibody mixes, control gates were set before collection, and the same analysis gates applied to all samples.

Reporting for specific materials, systems and methods

We require information from authors about some types of materials, experimental systems and methods used in many studies. Here, indicate whether each material, system or method listed is relevant to your study. If you are not sure if a list item applies to your research, read the appropriate section before selecting a response.

Materials & experimental systems

n/a	Involved in the study
[ ]	[x] Antibodies
[ ]	[x] Eukaryotic cell lines
[x]	[ ] Palaeontology and archaeology
[x]	[ ] Animals and other organisms
[x]	[ ] Clinical data
[x]	[ ] Dual use research of concern
[x]	[ ] Plants

Methods

n/a	Involved in the study
[x]	[ ] ChIP-seq
[ ]	[x] Flow cytometry
[x]	[ ] MRI-based neuroimaging

Antibodies

Antibodies used	CHX10/VSX2 (Santa Cruz Biotechnology sc-365519; 1:300 dilution), CHX10/VSX2 (Millipore AB9016; 1:300 dilution), SOX2 (Cell Signaling Technology 3579; 1:300 dilution), TBXT (Invitrogen PA5-46984; 1:300 dilution), CDX2 (Cell Signaling Technology 12306; 1:300 dilution), PAX6 (Developmental Studies Hybridoma Bank PAX6; 1:50 dilution), TUBB3/TUJ1 (BioLegend 802001; 1:1000 dilution), LHX3 (DSHB 67.4E12-s; 1:50 dilution), TUBB3/TUJ1 (BioLegend 801201; 1:1000 dilution), SOX21 (R&D Systems AF3538; 1:200 dilution), SOX14 (Novus Biologicals NBP2-94603; 1:200 dilution)
Validation	All antibodies were purchased from reputable commercial vendors with previous verification data. Links to antibody manufacturer verification data are provided in the Supplementary Data files

Eukaryotic cell lines

Policy information about cell lines and Sex and Gender in Research

Cell line source(s)	Human embryonic stem cell line H9 (female line, WiCell, RRID: CVCL_1240), Human induced pluripotent stem cells WTC11 (male; Coriell Institute, RRID: CVCL_Y803), Human induced pluripotent stem cells WTB (female; gift from Bruce Conklin; Miyaoka et al., 2014).
Authentication	All cell lines were obtained from reputable vendors or sources and previously verified in scientific publications.
Mycoplasma contamination	All cell lines routinely tested negative for mycoplasma contamination throughout the study's duration.
Commonly misidentified lines (See ICLAC register)	No cell lines in the ICLAC Register were used in this study.

Plants

Seed stocks	N/A
Novel plant genotypes	N/A
Authentication	N/A

Flow Cytometry

Plots

Confirm that:

- [x] The axis labels state the marker and fluorochrome used (e.g. CD4-FITC).
- [x] The axis scales are clearly visible. Include numbers along axes only for bottom left plot of group (a 'group' is an analysis of identical markers).
- [x] All plots are contour plots with outliers or pseudocolor plots.
- [x] A numerical value for number of cells or percentage (with statistics) is provided.

Methodology

Sample preparation	For flow cytometry, cells were dissociated with Accutase (StemCell Technologies, 07920) at 37C for 10-20 minutes and fixed

	and permeabilized using the FOXP3/Transcription factor staining buffer kit (eBiosciences). Samples were stored at 4C until staining and analysis. Cells were sequentially stained with primary and secondary antibodies as described.
Instrument	Attune NxT Flow Cytometer (ThermoFisher)
Software	Flowjo™ (FlowJo™ Software Version 8.7)
Cell population abundance	A minimum of 10,000 events were collected for each sample.
Gating strategy	Gates were set using unstained and secondary-only stained samples for negative populations. Positive control cell populations were used when available. Primary and secondary gates were made to exclude cell debris and to isolate single cells before examining fluorescent populations of interest.

☒ Tick this box to confirm that a figure exemplifying the gating strategy is provided in the Supplementary Information.
