# Supplementary material for: Distinct differentiation trajectories leave lasting impacts on gene regulation and function of V2a neurons
Source: Nat Commun. 2026 Jul 29;17:9187. doi: 10.1038/s41467-026-76054-w (PMC13522347; doi:10.1038/s41467-026-76054-w)
Supplement: Supplementary file 2 [file 41467_2026_76054_MOESM1_ESM.pdf]

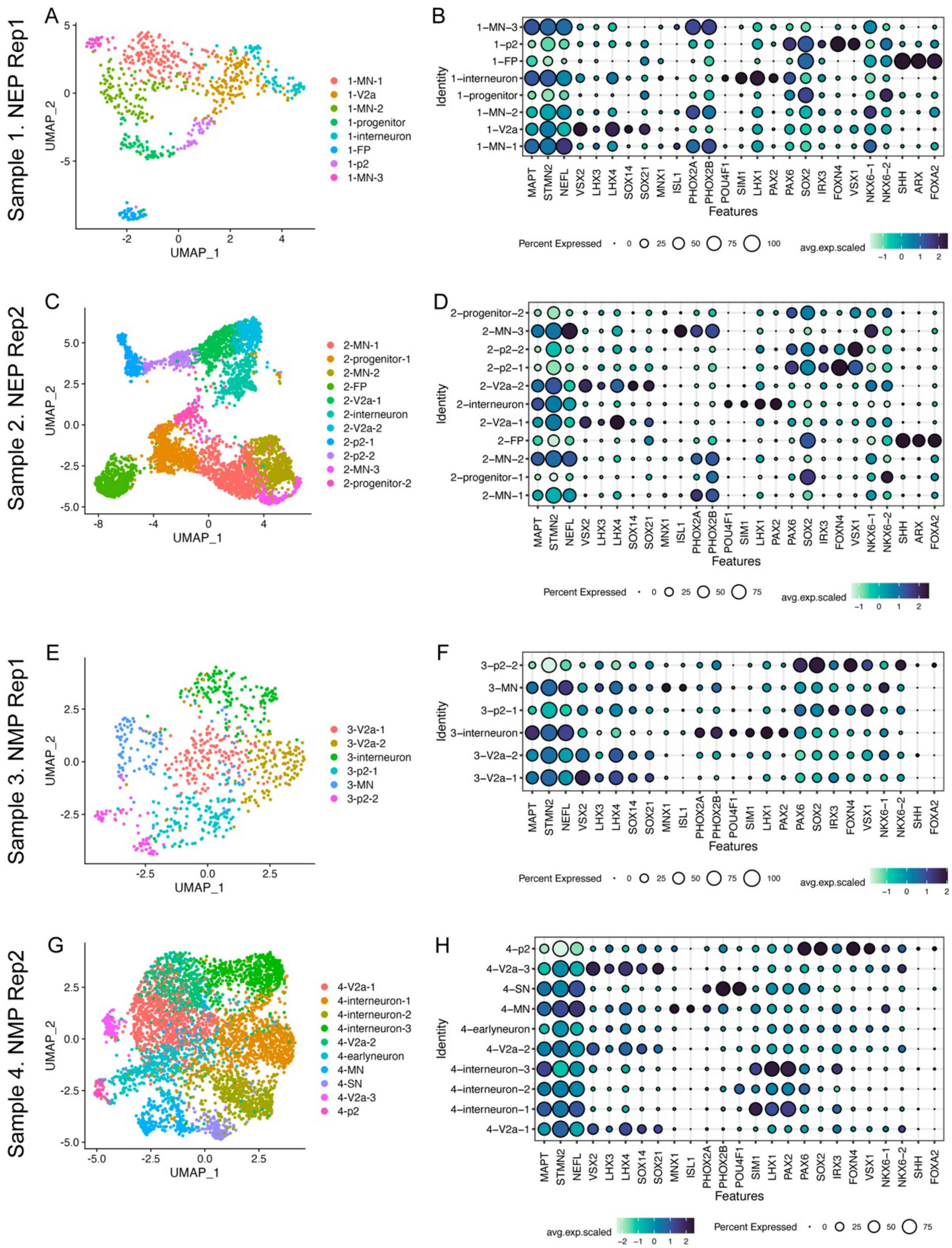

Supplemental Figure 1. Individual sample clustering and cell annotation by marker gene sets.
A, C, E, G. UMAP plots of the indicated sample with clusters identified using resolution = 0.8.
B, D, F, H. Dot plots showing the expression of marker gene sets used to classify cell types within the indicated sample.

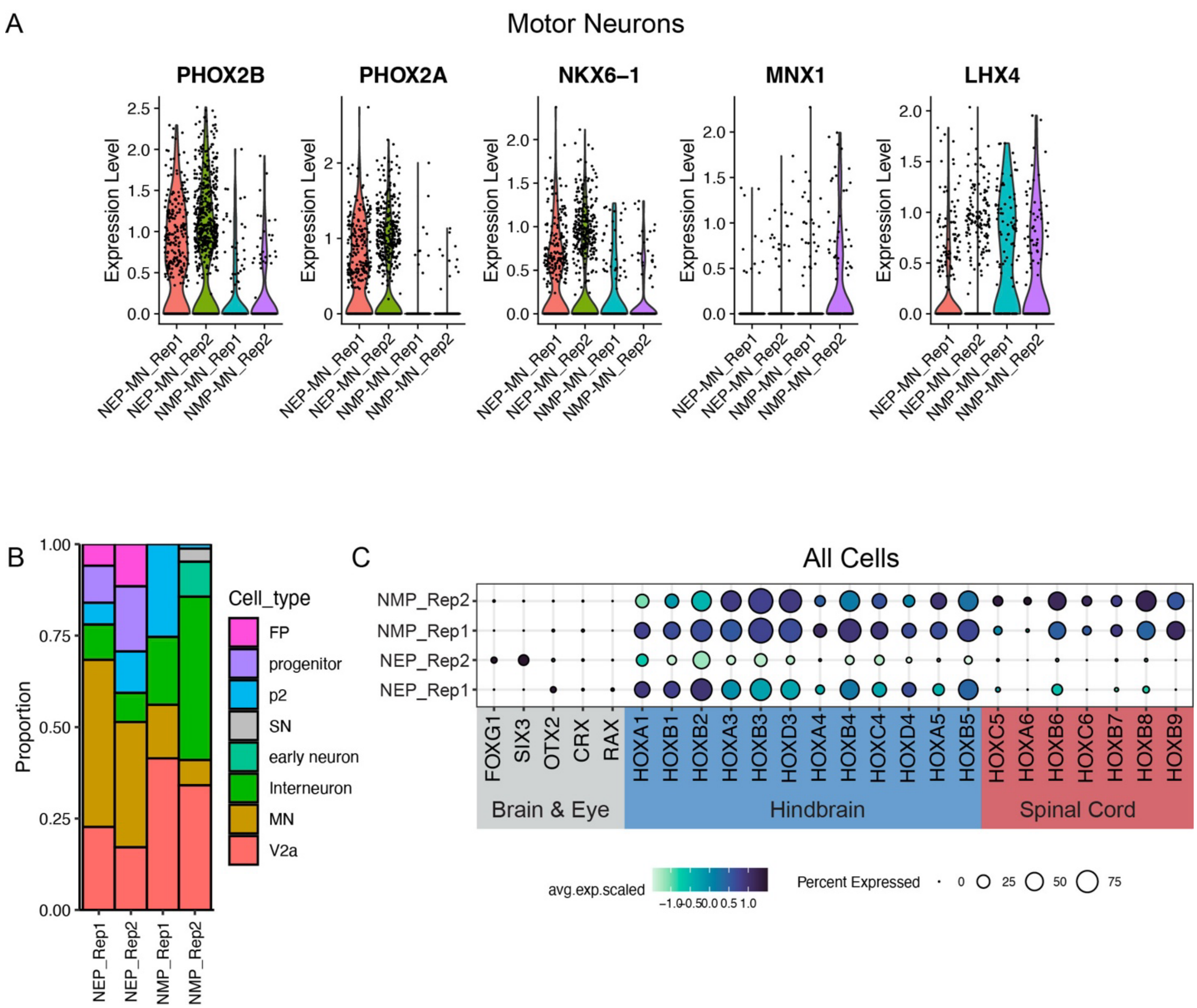

Supplemental Figure 2. Characterization of population proportion and axial identity in multiome samples.

A. Motor neuron marker gene expression between NEP- and NMP-derived samples.

B. Bar plot showing the proportion of each annotated cell type for each multiome sample.

C. Expression of anterior-posterior axial markers in each multiome sample. Colored groups of genes indicate approximate anatomical boundaries.

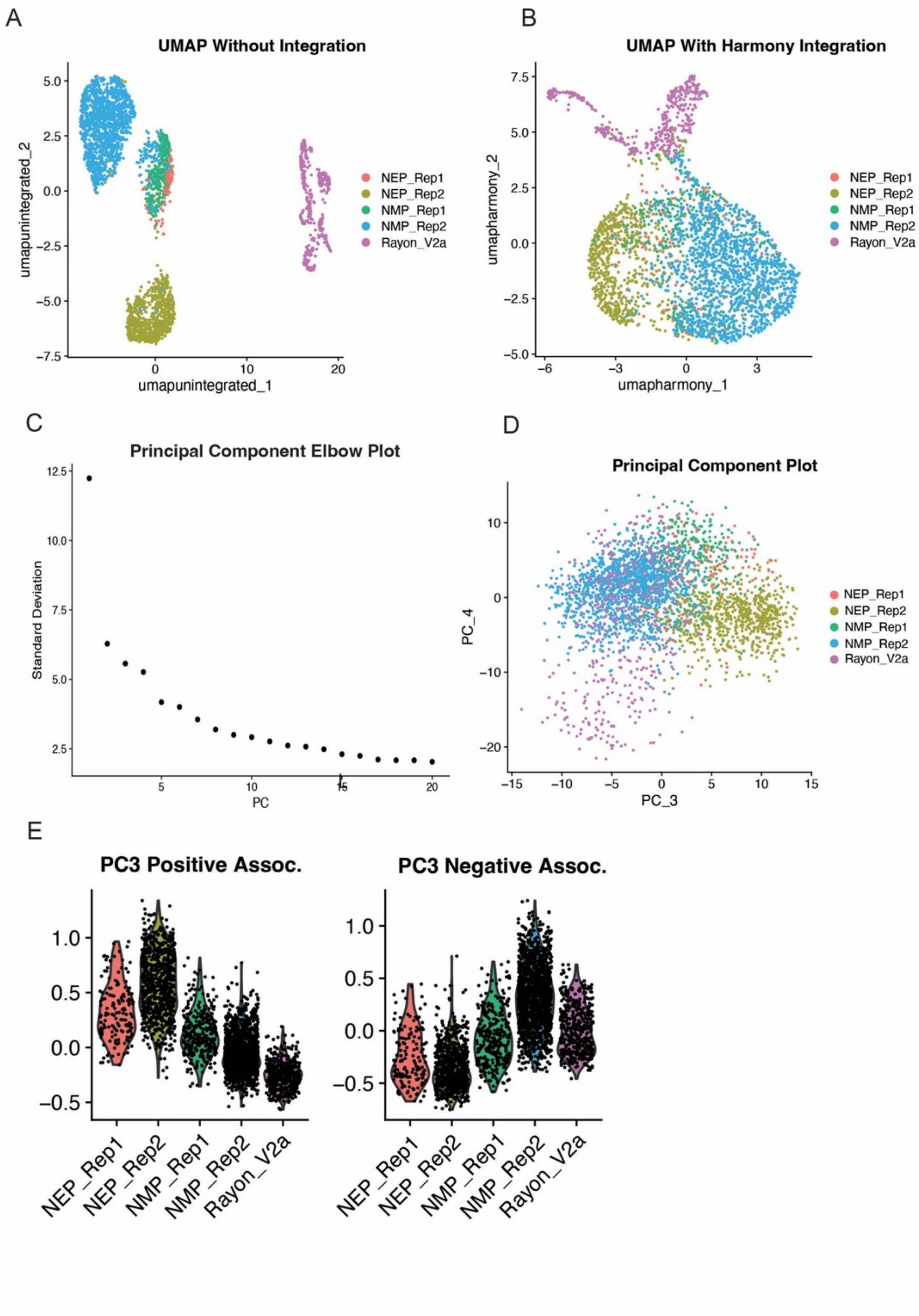

Supplemental Figure 3. Comparison of in vitro and in vivo V2a samples.

A. UMAP showing subset of only V2a neurons without integration.
B. UMAP showing subset of only V2a neurons with Harmony integration.
C. Elbow plot of each principal component showing how much variation each component describes.
D. Scatter plot of principal components 3 and 4 showing separation of NEP- and NMP-derived V2a neurons and overlap with in vivo V2a neurons.
E. Module score of each sample for the top 25 genes which have positive and negative correlations with principal component 3.

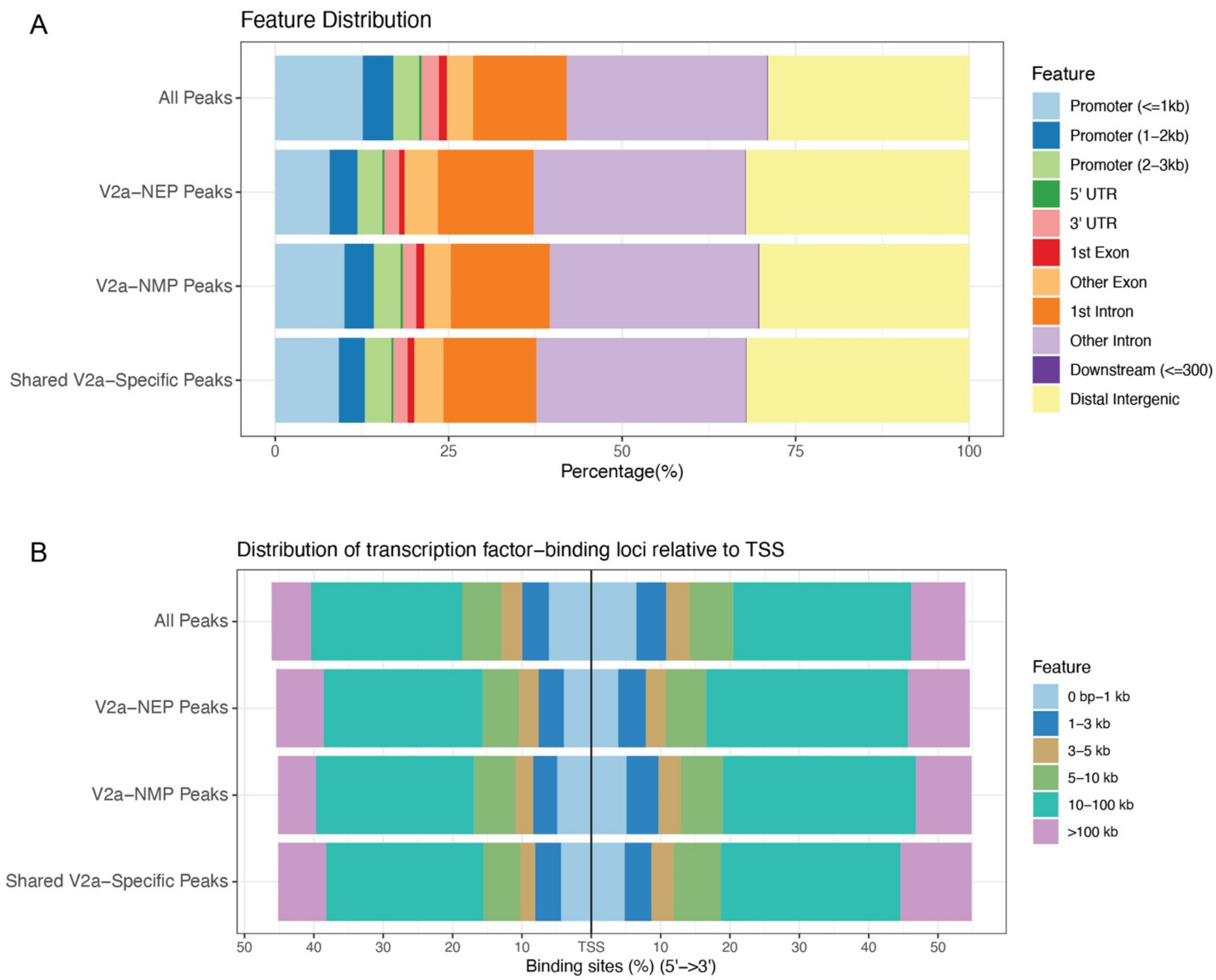

Supplemental Figure 4. Similarities in the annotation and relative location of differentially accessible ATAC peaks between NEP and NMP V2a neurons.

A. Gene feature annotation for the gene with the nearest TSS of differentially accessible chromatin peaks for the indicated peak sets.
B. Peak distance annotation relative to the nearest TSS of differentially accessible chromatin peaks for the indicated peak sets.

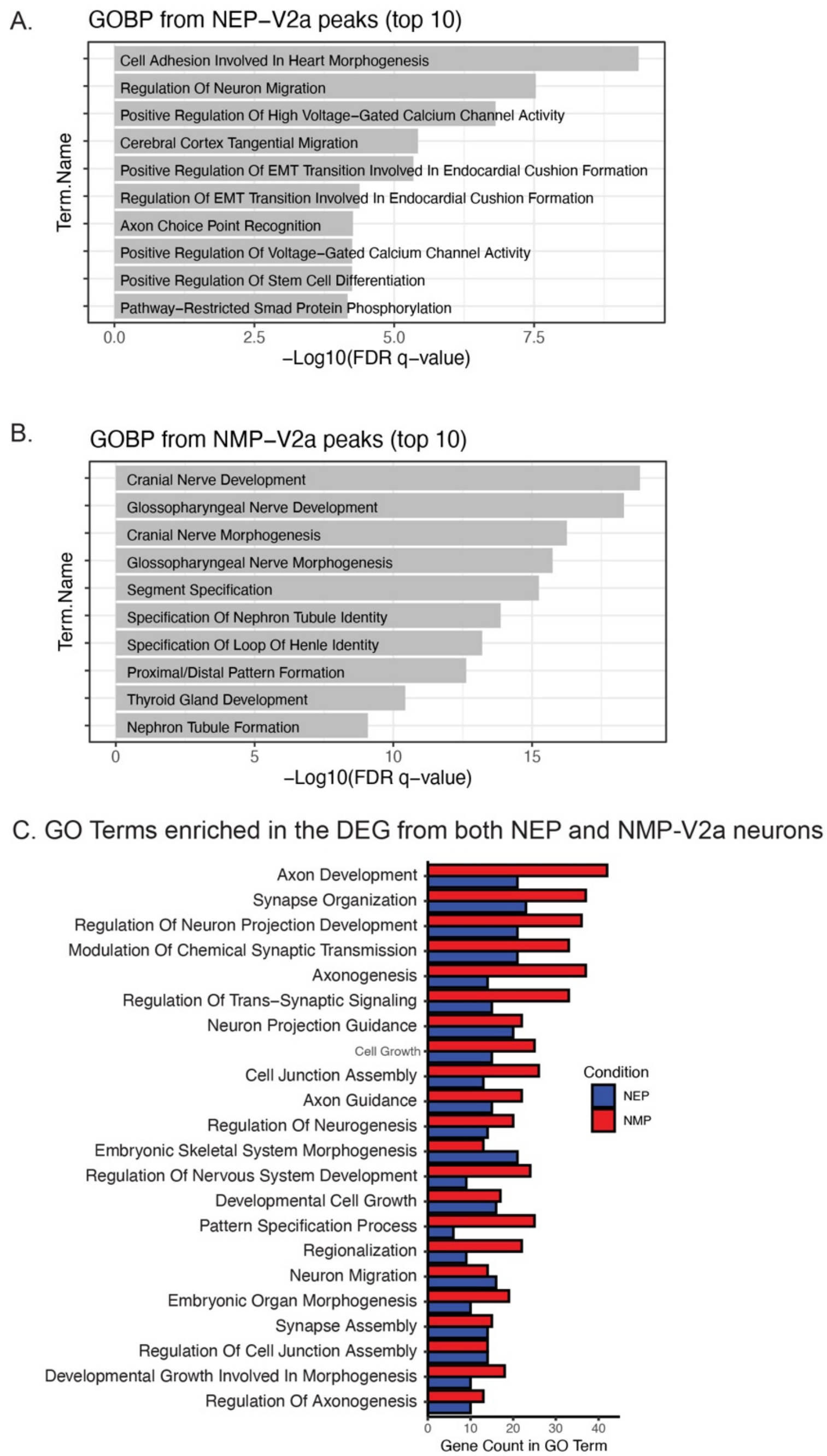

Supplemental Figure 5. Enrichment of Gene Ontology terms for the genes near ATAC peaks.

A. Top 10 significantly enriched biological process GO terms for the genes near NEP-enriched peaks as identified by the GREAT Tool.

B. Top 10 significantly enriched biological process GO terms for the genes near NMP-enriched peaks as identified by the GREAT Tool.

C. Shared significantly enriched GO biological process terms identified in the differentially expressed genes between NEP and NMP-derived V2a neurons. Bars represent the number of genes annotated in each respective ontology term in each condition

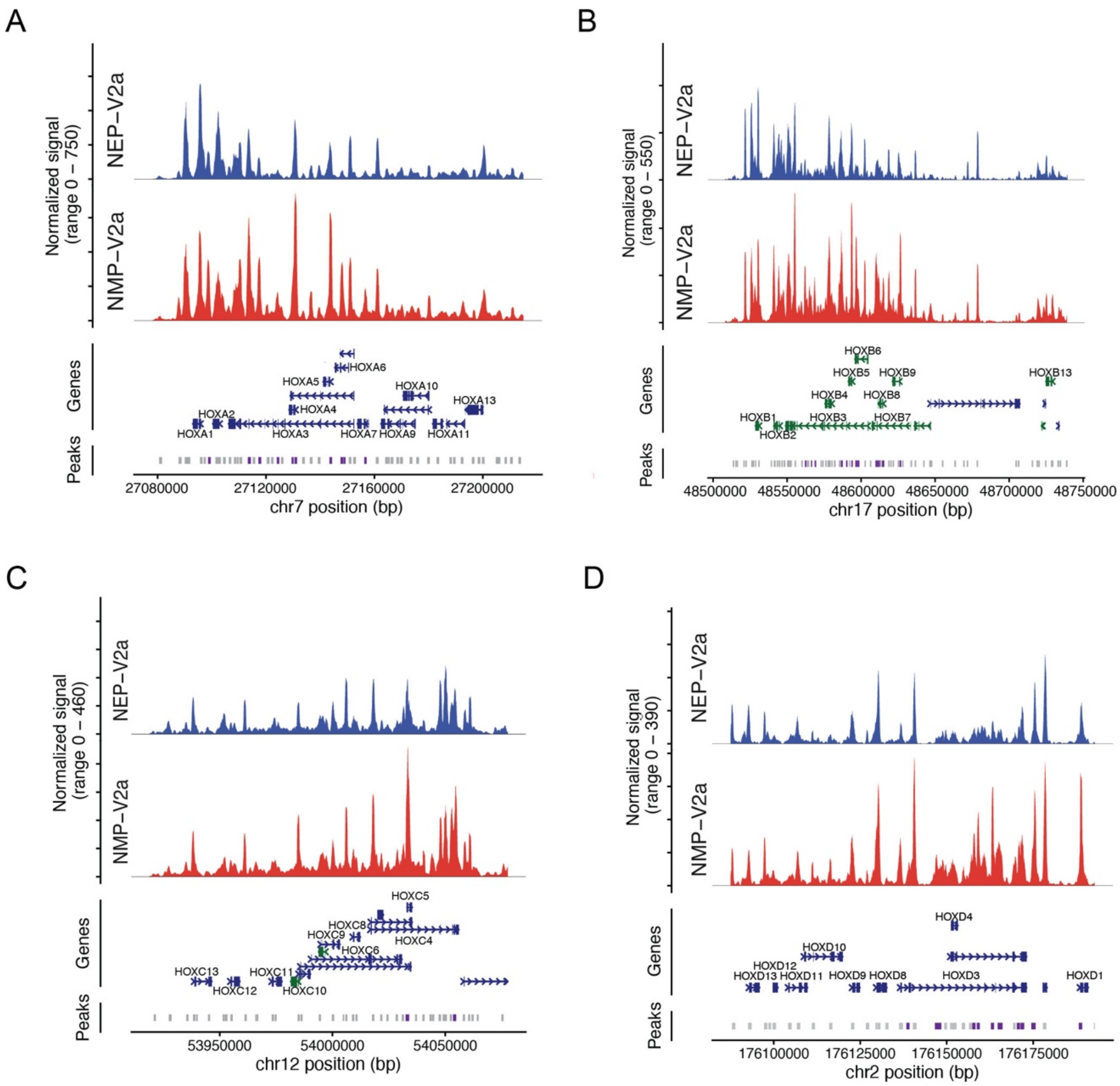

Supplemental Figure 6. Analysis of differentially accessible regions in HOX gene clusters in V2a neurons.

A. Comparison of NEP- and NMP-derived V2a neuron ATAC-seq peaks at the HOXA gene cluster. 10 peaks were found to be significantly enriched in the NMP-derived condition (purple, bottom row).
B. Comparison of NEP- and NMP-derived V2a neuron ATAC-seq peaks at the HOXB gene cluster. 13 peaks were found to be significantly enriched in the NMP-derived condition (purple, bottom row).
C. Comparison of NEP- and NMP-derived V2a neuron ATAC-seq peaks at the HOXC gene cluster. 2 peaks were found to be significantly enriched in the NMP-derived condition (purple, bottom row).
D. Comparison of NEP- and NMP-derived V2a neuron ATAC-seq peaks at the HOXD gene cluster. 10 peaks were found to be significantly enriched in the NMP-derived condition (purple, bottom row).

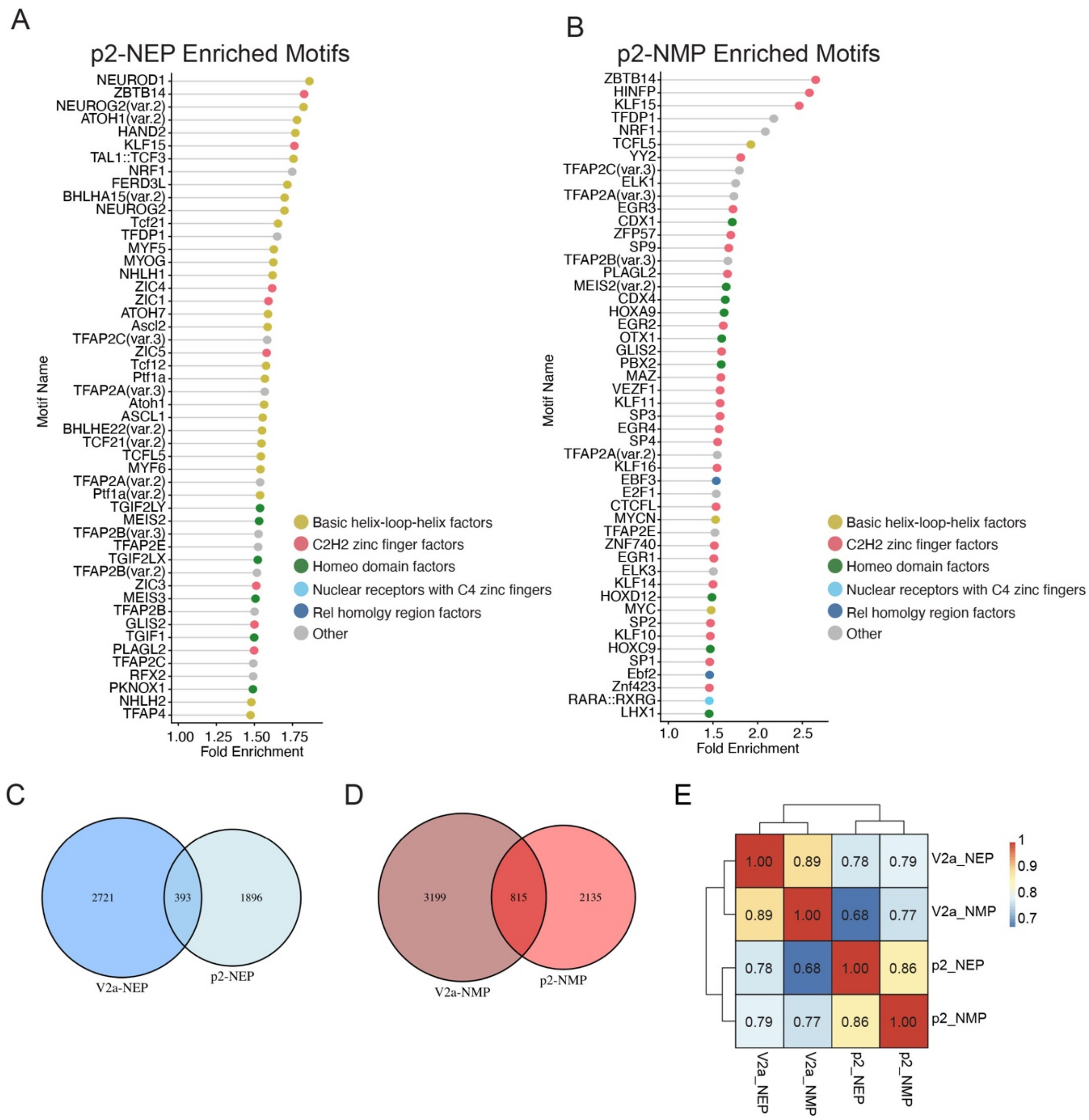

Supplemental Figure 7. Lineally distinct p2 progenitors possess enriched transcription factor motifs similar to V2a but limited overlap in lineage specific ATAC peaks.

A. Top 50 most enriched transcription factor motifs in NEP p2 DARs ranked by fold enrichment. Each motif is colored by transcription factor class.

B. Top 50 most enriched transcription factor motifs in NMP p2 DARs ranked by fold enrichment. Each motif is colored by transcription factor class.

C. Venn diagram of the DARs found in both the p2 and V2a cells from the NEP condition

D. Venn diagram of the DARs found in both the p2 and V2a cells from the NMP condition

E. Pearson diagram showing the global similarity of ATAC peaks between p2 and V2a populations.

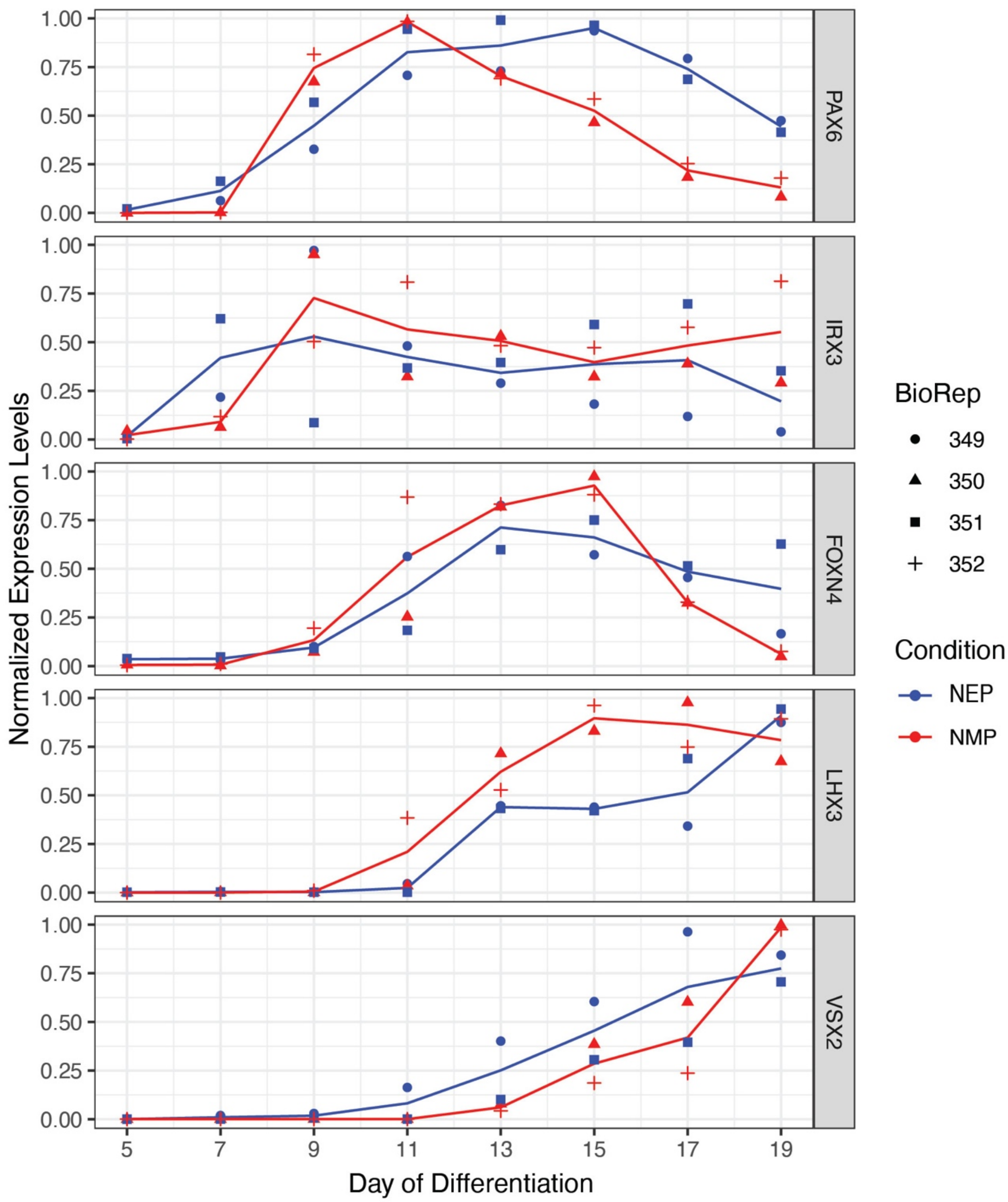

Supplemental Figure 8. Expression of V2a development genes during differentiation. Time course qPCR data showing normalized expression values of each gene stratified by differentiation condition between days 5 to 19 of each protocol. n = 2 biological replicates per time point. Each point represents the average of two technical replicates.

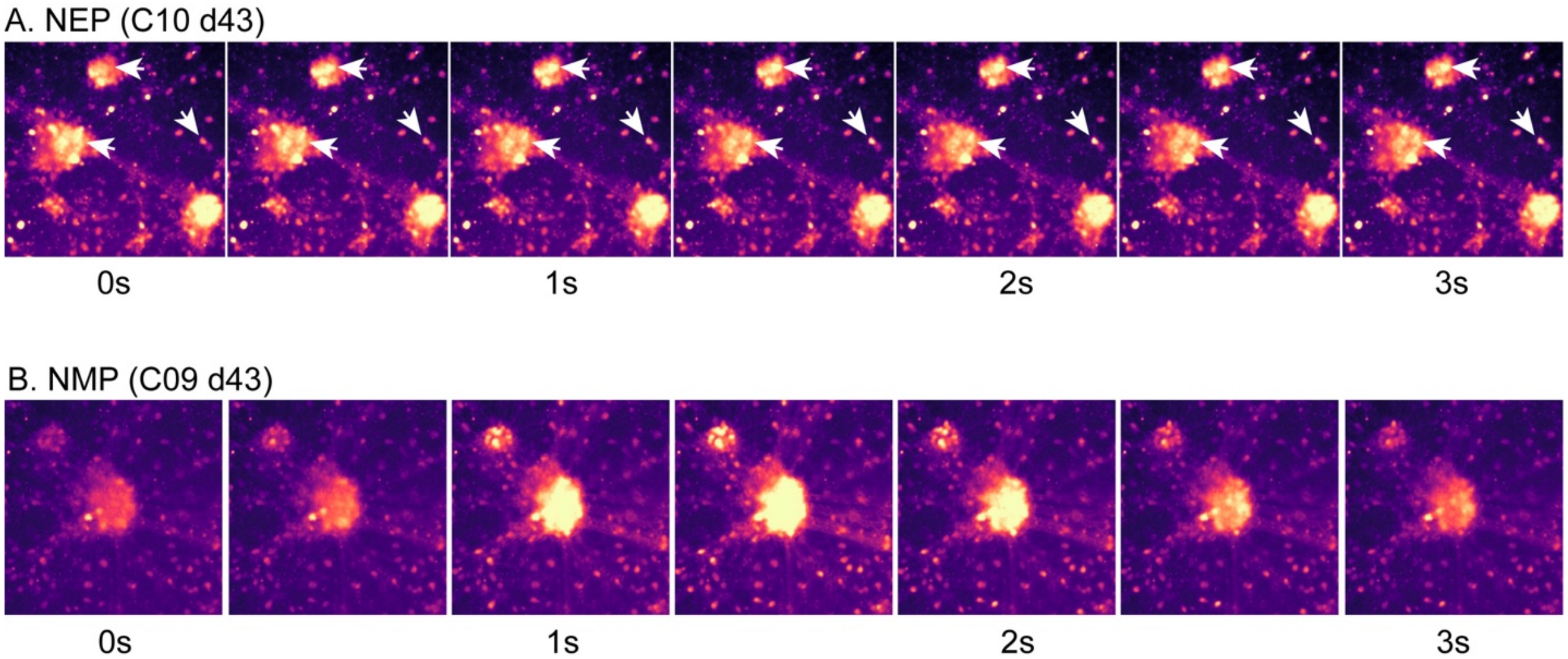

Supplemental Figure 9. Representative stills of neural activity from calcium imaging videos.

A. Representative images from a d43 NEP-derived neuron culture calcium imaging video. Arrow heads point to regions with fluorescent activity.
B. Representative images from a d43 NMP-derived neuron culture calcium imaging video. Panels show a synchronous burst of activity across the population.

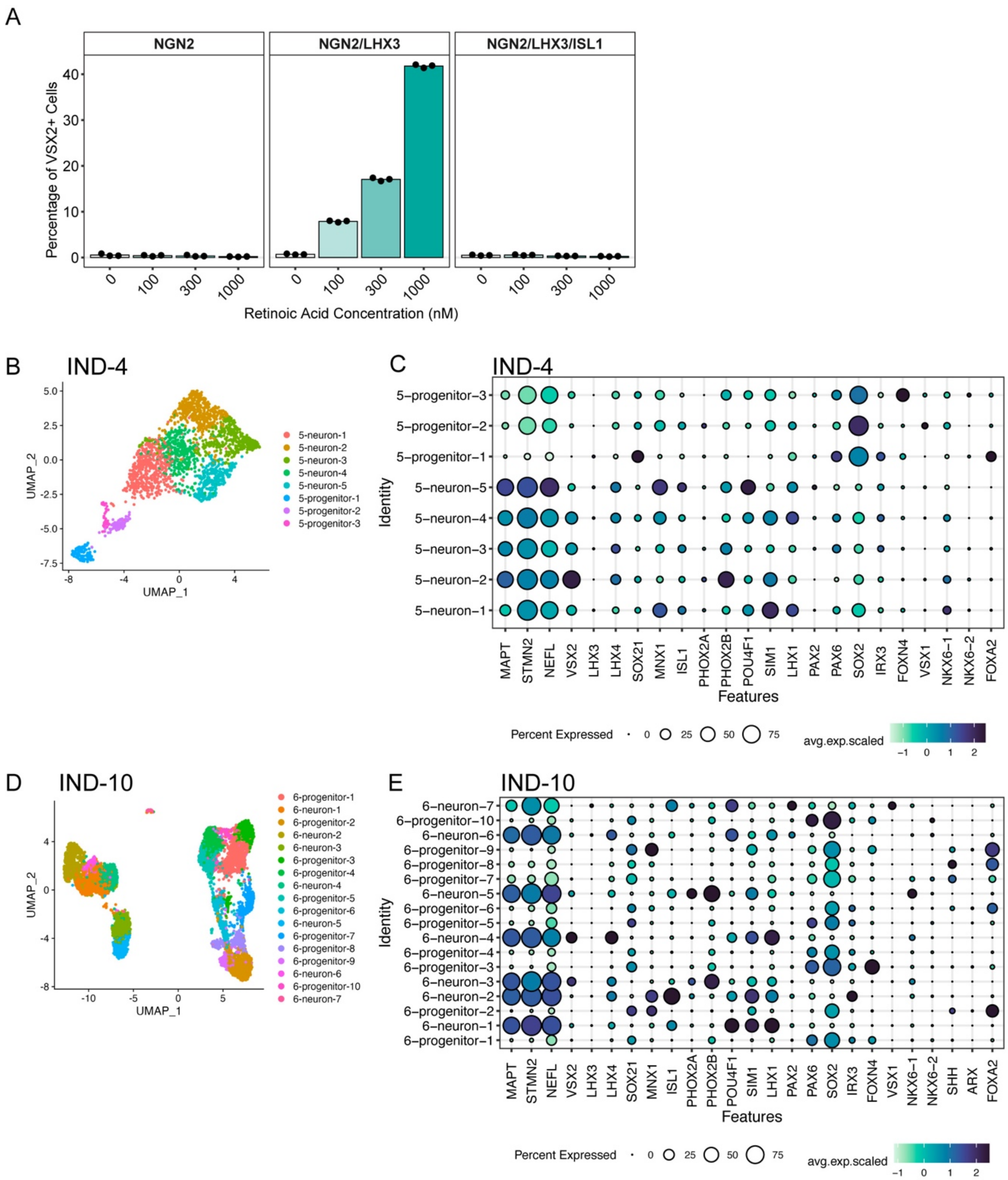

Supplemental Figure 10. Marker gene expression in induced neurons.

A. Flow cytometry quantification of VSX2 expression in induced neuron populations under graded concentration of retinoic acid (RA). The specific combination of transcription factors is shown at the top of each panel. n = 3 technical replicates.
B. UMAP of day 4 induced neurons.
C. Marker gene expression in clusters identified in day 4 induced neurons
D. UMAP of day 10 induced neruons.
E. Marker gene expression in clusters identified in day 10 induced neurons.

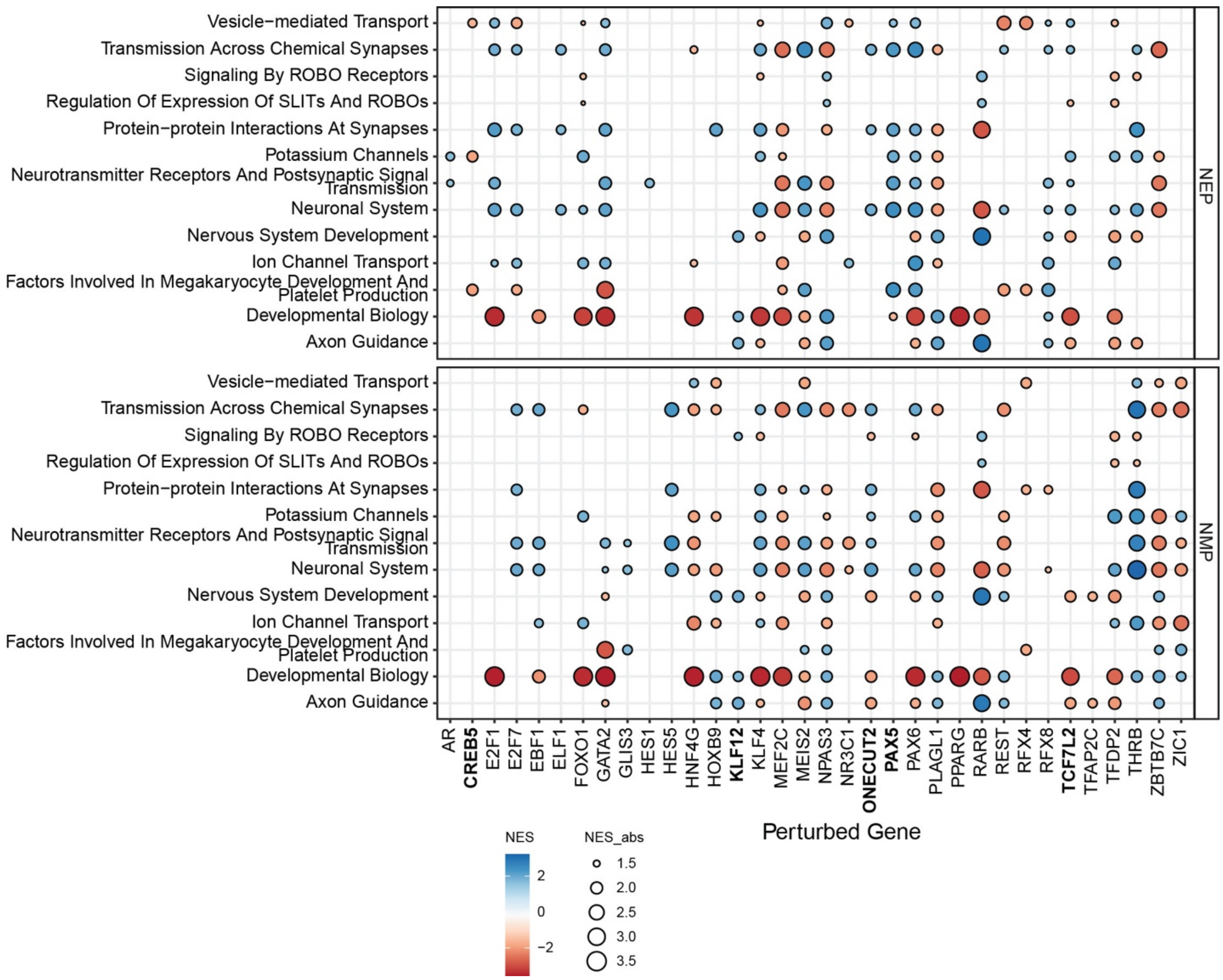

Supplemental Figure 11. GSEA in distinct V2a populations after in silico knockout of highly central transcription factors. GSEA normalized enrichment scores for selected gene sets after predicting CellOracle gene knockout effects in both NEP and NMP V2a neurons. Target genes for CellOracle knockout are from the most central genes in the predicted V2a gene regulatory networks.

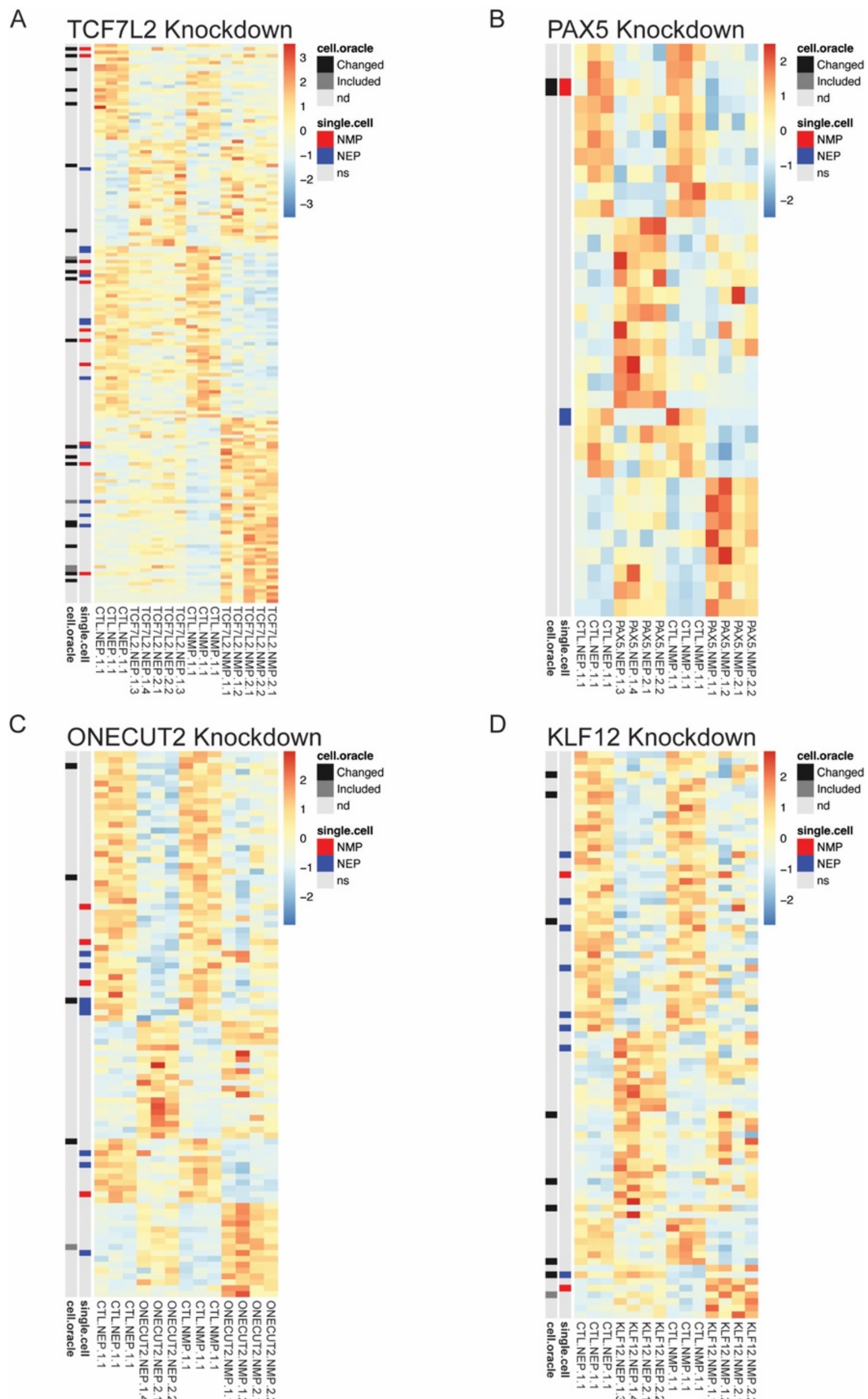

Supplemental Figure 12. Differential gene expression resulting from transcription factor knockdown in NEP and NMP differentiations.

A. Heatmap depicting the significantly differentially expressed genes in the TCF7L2 KD condition across both NEP and NMP differentiations.
B. Heatmap depicting the significantly differentially expressed genes in the PAX5 KD condition across both NEP and NMP differentiations.
C. Heatmap depicting the significantly differentially expressed genes in the ONECUT2 KD condition across both NEP and NMP differentiations.
D. Heatmap depicting the significantly differentially expressed genes in the KLF12 KD condition across both NEP and NMP differentiations.

Supplemental Note: Generation of a FOXN4:Cre lineage reporter

The set of specific markers that exist for V2a neurons are made up of intracellular proteins, largely transcription factors, and no studies to date have identified specific surface molecules that would allow for live staining and recovery. Therefore, to enable live assessment and single cell sorting of presumptive V2a neurons, we implemented a genetically encoded lineage tracing system. The transcription factor FOXN4 is a marker that is specifically expressed in the p2 progenitor domain which gives rise to the V2 family of interneurons (i.e., V2a, V2b; Figure S14A). Although specific, FOXN4 expression is transient, so a Cre-based recombination strategy was preferred. Since FOXN4 expression precedes VSX2, we reasoned that this recombination strategy would reliably mark VSX2+ cells by the time its expression was upregulated. Previous studies with V2a differentiations showed that the inclusion of DAPT potently inhibits generation of V2b/c cells, minimizing off targets. Lastly, since FOXN4 is a pan-V2 marker, this lineage trace strategy could be used in other contexts to study V2b cells or to isolate a balanced population of excitatory and inhibitory neurons.

We built the reporter into a WTC11-derived cell line which had been edited to contain a lox:STOP:lox reporter cassette in the CLYBL safe harbor locus which was generously gifted by Dr. Luke Judge. Briefly, WTC-hNIL transgenic iPSC (previously described in Shi et al., 2018; Fernandopulle et al., 2018) were further modified by integrating the 8.4kb XR1 (eXcision Reporter 1) transgene into the CLYBL safe harbor locus. The XR1 donor plasmid was derived from pC13N-dCas9-BFP-KRAB (Tian et al., 2019 and a gift from Martin Kampmann) by replacing the dCas9-BFP-KRAB coding sequence with a lox-STOP-lox-eGFP-P2A-mTagRFP coding sequence. The construct is designed such that CAG driven expression of the fluorescent reporter is prevented until the STOP terminator sequence is removed, either by Cre-Lox recombination or nuclease excision, similar to the Ai9 murine reporter (Figure S13A, Madisen et al., 2010). To generate the cell line, 1 x 106 WTC-hNIL iPSC were added to 100 µL P3 buffer with 6 µg of the XR1 donor plasmid, and 3 µg of each paired TALEN (Addgene, 62196/62197) targeting the CLYBL locus (Cerbini et al., 2015). Cells were transfected using the P3 Primary Cell 4D-Nucleofector X Kit L (Lonza, V4XP-3024) with pulse code DS138. After nucleofection, cells incubated for 5 minutes at room temperature and were then plated in Stemfit media with 10 µm Y-27632 in a serial dilution into 5 wells of a 6-well plate. Non-transfected cells were plated in the sixth well. Two days post-nucleofection media was exchanged for Stemfit containing G418 and 10 µM Y-27632. Cells were kept on G418 selection until all cells in the control wells were dead and only red fluorescent colonies remained in the experimental wells. Fluorescent colonies were manually picked and transferred to a new culture plate for expansion. Individual clones were screened for on-target integration via junction PCR using a vector-specific primer paired with a flanking primer outside of the CLYBL homology arms in the donor vector (Supplementary Data 1). The final clone (clone 4) was selected based on single copy integration confirmed using a ddPCR assay with a FAM-labeled probe targeting the Neomycin resistance gene and a HEX-labelled RPP30 reference probe.

Five unique guide RNA were designed targeting the left side (L1-5) and five targeting the right side (R1-5) of the lox-STOP-lox sequence to test CRISPR-mediated activation of the

reporter by excision of the terminator (Figure S13A, Supplementary Data 1). We compared single guide RNA (Synthego) with complexed crRNA/tracrRNA (IDT) and tested all pairwise combinations of left and right guide RNA. We nucleofected 2.5 x 105 XR1 iPSC in 20 uL of P3 buffer with 10 pmol Cas9 protein (Macrolab) complex with 30 pmol of each synthetic gRNA using pulse code DS138. Immediately after nucleofection cells were plated in 96-well plates. Green fluorescence was observed by microscopy within 24 hours and four days after nucleofection the cells were dissociated with accutase and analyzed by high-throughput flow cytometry to measure the percentage of GFP-positive cells (Figure S13B-D).

We next made a donor plasmid (available upon request) for integration of a P2a cleaved Cre in-frame with the endogenous FOXN4 locus on chromosome 12 (Figure S14A). The donor plasmid contained a pCAGG::mCherry integration reporter for non-antibiotic selection which could be removed by CRISPR/Cas9 using flanking tia1L guide sites (Roberts et al., 2019, fragments taken from AICSDP-45: TTN-mEGFP, a gift from Allen Institute for Cell Science; Addgene # 114412). A homology region of ~2 kb in length surrounding the FOXN4 stop codon in exon 10 was amplified from genomic DNA isolated from WTC11 cells using primer set 1 in Supplementary Data 2 and inserted into a pCR-BluntII-TOPO backbone. After a first round of colony selection and purification, PCR amplification with primer set 2 (Supplementary Data 2) was used to 'open' the homology region and insert the P2a::Cre::selection cassette using the NEBuilding HiFi DNA Assembly Master Mix (NEB). Plasmid clones were sanger sequenced to confirm that there were no indels.

For integration into the WTC11-XR1 cells, CRISPR/Cas9 homology directed repair was used. A modified single-guide RNA (sgRNA; ACAGCUCAAAGCAGGGCUAU) was purchased from Synthego and reconstituted according to manufacturer's guidelines. 3 uL of 100 uM sgRNA was complexed with 1 uL of AltR-Cas9-NLS in TE buffer for 10 minutes at room temp to form RNP. RNP and 500 ng of donor plasmid were mixed in 20 uL of prepared P3 buffer and nucleofected into 200k Accutased and resuspended WTC11-XR1 stem cells using a P3 Primary Cell 4D Nucleofector kit and a Lonza Nucleofector 4D device with EN138 cell line code. Nucleofected stem cells were recovered in fresh media with 10 uM Y27632 for 48 hours. Clonally integrated FOXN4:Cre stem cell lines were generated by sparsely plating single stem cells into a 10 cm dish and allowing individual colonies to form. mCherry+ colonies were hand-picked, transferred to individual wells of a 96-well plate, and expanded. Clones were genotyped to ensure proper location and orientation of the insertion and also passed a test for chromosomal abnormalities using the hPSC Genetic Analysis Kit (StemCell Technologies). To remove the mCherry selection cassette, first 1 uL AltR-Cas9-NLS and 3 uL of 100 uM Tia1L sgRNA (GGUAUGUCGGGAACCUCUCC, Synthego) were complexed in TE for 10 minutes. 0.3 uL of RNP was mixed with 3 uL of Lipofectamine Stem in 150 uL of OptiMEM. 25 uL of this mixture was added per well of a 24-well plate. After one passage, mCherry- cells (~30% of the population) were sorted on a FACS Aria Fusion, recovered, expanded, and cryopreserved.

In the final stem cell line, heterozygous FOXN4:Cre integration could by shown by PCR around the integration site (Figure S14B). To validate the Cre activity, we performed V2a differentiation. Live fluorescent imaging at days 10, 13, and 17 of differentiation show progressive acquisition of GFP signal, in agreement with temporal FOXN4 gene expression observed in time-matched qPCR samples (Figure S14C,F). While we found that the FOXN4:Cre GFP signal enriches our population of interest, it did not exclusively label all V2a cells. In both flow cytometry and immunofluorescence, we found roughly equivalent proportions of the following populations: VSX2+GFP+, VSX2+GFP-, and VSX2-GFP+ (Figure S14D, Figure S15). One hypothesis for why some V2a neurons escaped labeling is that the prolonged addition of DAPT results in rapid maturation of neural progenitors and the window of FOXN4 expression is restricted. However, the proportion of GFP+ cells correlated with the total percent of VSX2+ cells in each differentiation, strengthening the specificity of the lineage reporter (Figure S14E). Cells positively and negatively sorted by the GFP and RFP reporters showed enrichment of CRE, GFP, FOXN4 and VSX2 expression in the GFP+ RFP+ fraction by qPCR (Figure S14G). A small amount of recombination was also observed in the maintenance stem cell population, but this population was $< 0.5\%$ by flow cytometry and unlikely to represent a significant contaminating population (data not shown).

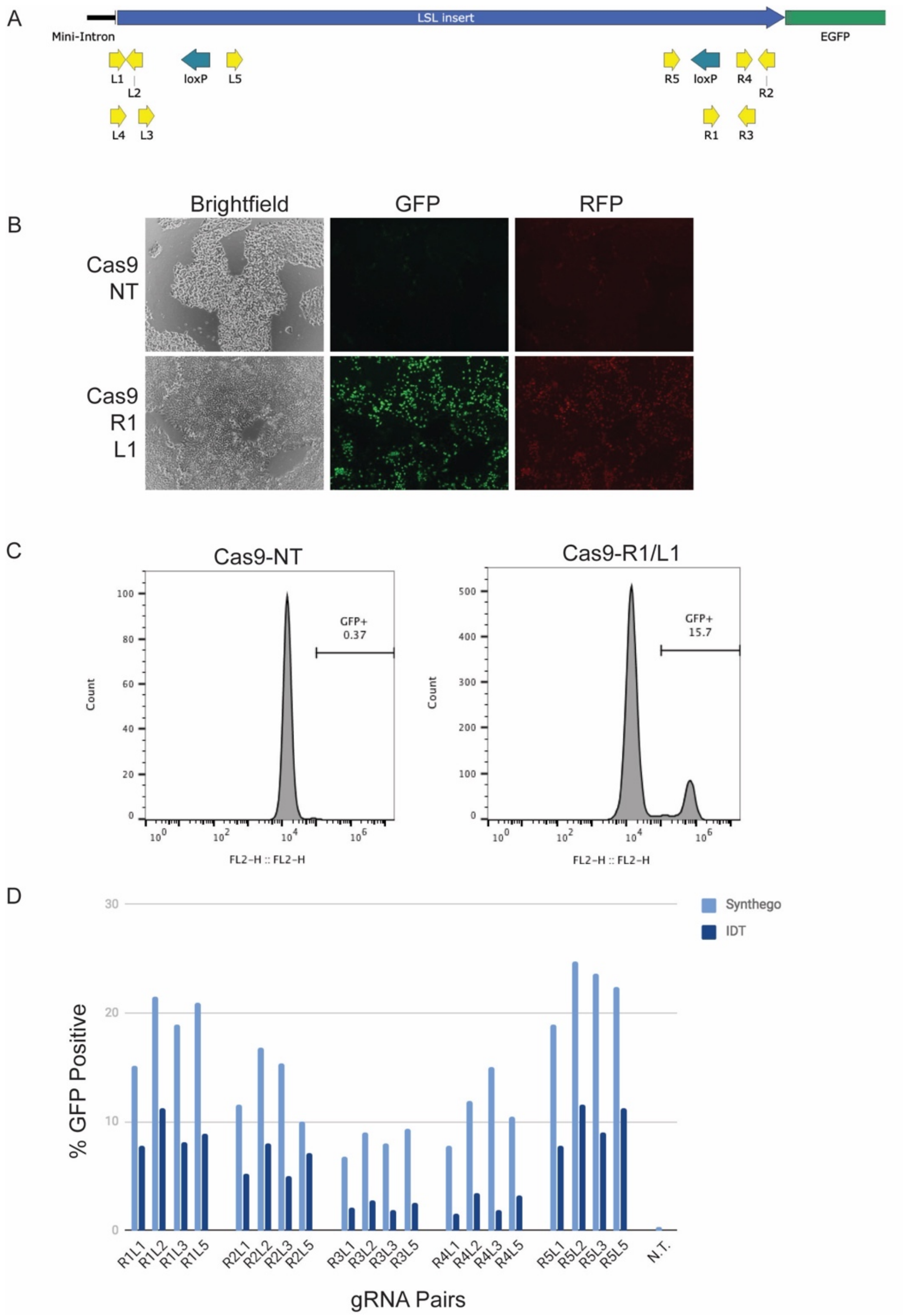

Supplemental Figure 13. Validation of XR1 reporter activation by CRISPR.

A. Diagrm of gRNA designed for excision of Lox-STOP-Lox (LSL) insert to activate expression of fluorescent reporter.
B. Images of XR1 iPSC after transfection with Cas9 ribonucleoprotein complexed with L1/R1 pair demonstrating activation of fluorescence reporter as compared to negative control with nontargeting gRNA (NT).
C. Flow cytometry demonstrating quantification of GFP-positive population shown in B.
D. Quantification of the percentage of GFP-positive cells activated by all combinations of gRNAs designed for excision-based reporter activation. Single gRNA shown in light blue (Synthego) compared to crRNA/tracrRNA complex (IDT) in dark blue

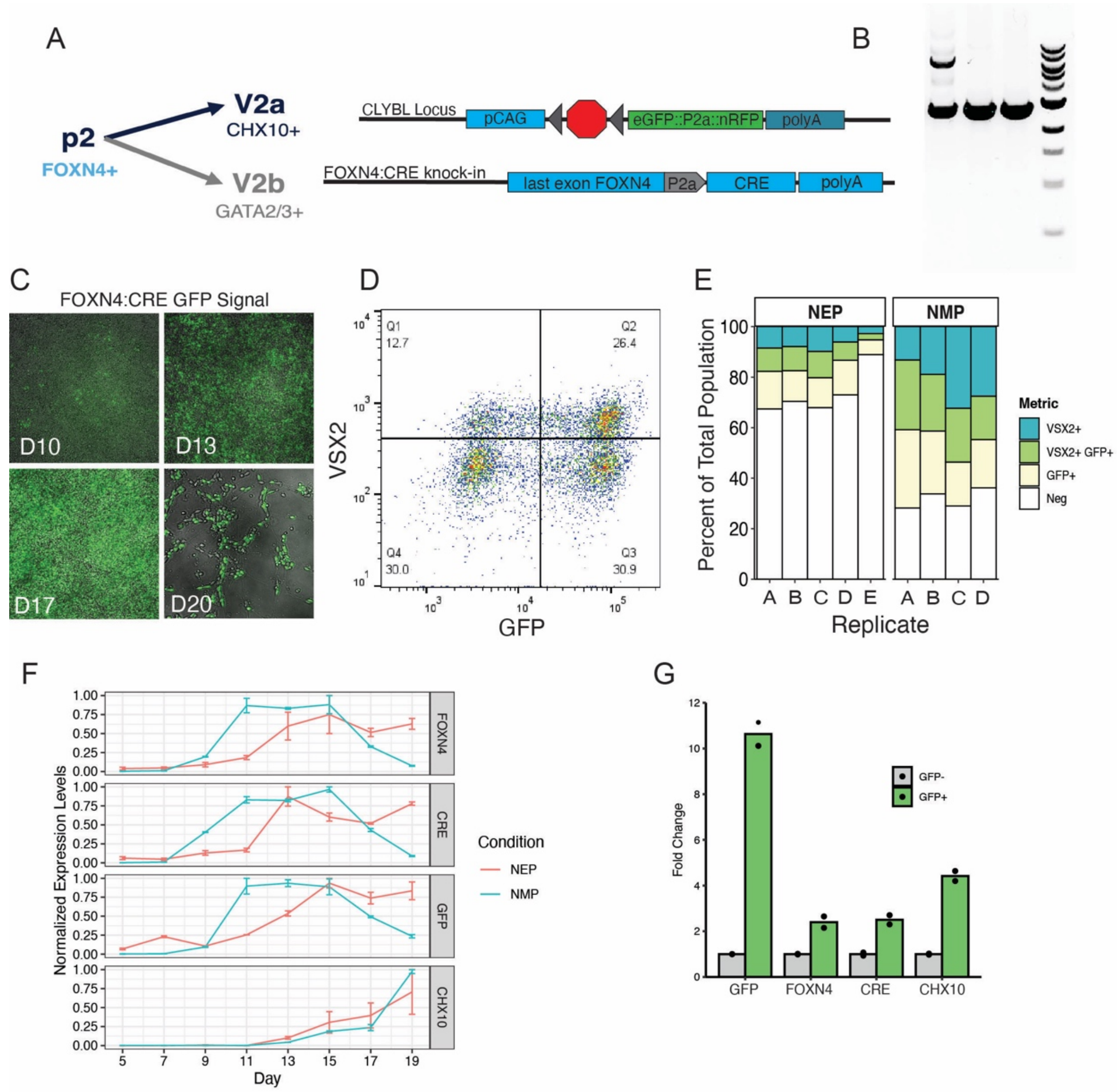

Supplemental Figure 14. Validation of a FOXN4:Cre lineage reporter for V2a differentiation.

A. (left) Diagram of the lineages marked by a FOXN4:Cre construct. (right) the final integrated constructs in the FOXN4:Cre line.
B. PCR of the endogenous FOXN4 locus using primers outside of the homology domain. Lane 1 is the heterozygous FOXN4:Cre line, Lane 2 is an unsuccessfully integrated clone. Lane 3 is the parental line.
C. Fluorescent images taken at the indicated day of differentiation of a V2a differentiation using the FOXN4:Cre line
D. Example flow cytometry of day 19 neurons showing GFP fluorescence against stained VSX2 fluorescence
E. Quantification of independent differentiations quantified by flow cytometry with gates as in D.
F. Time course qPCR of V2a and reporter lineage genes.
G. qPCR of populations of day 19 neurons sorted into GFP+ and GFP- populations

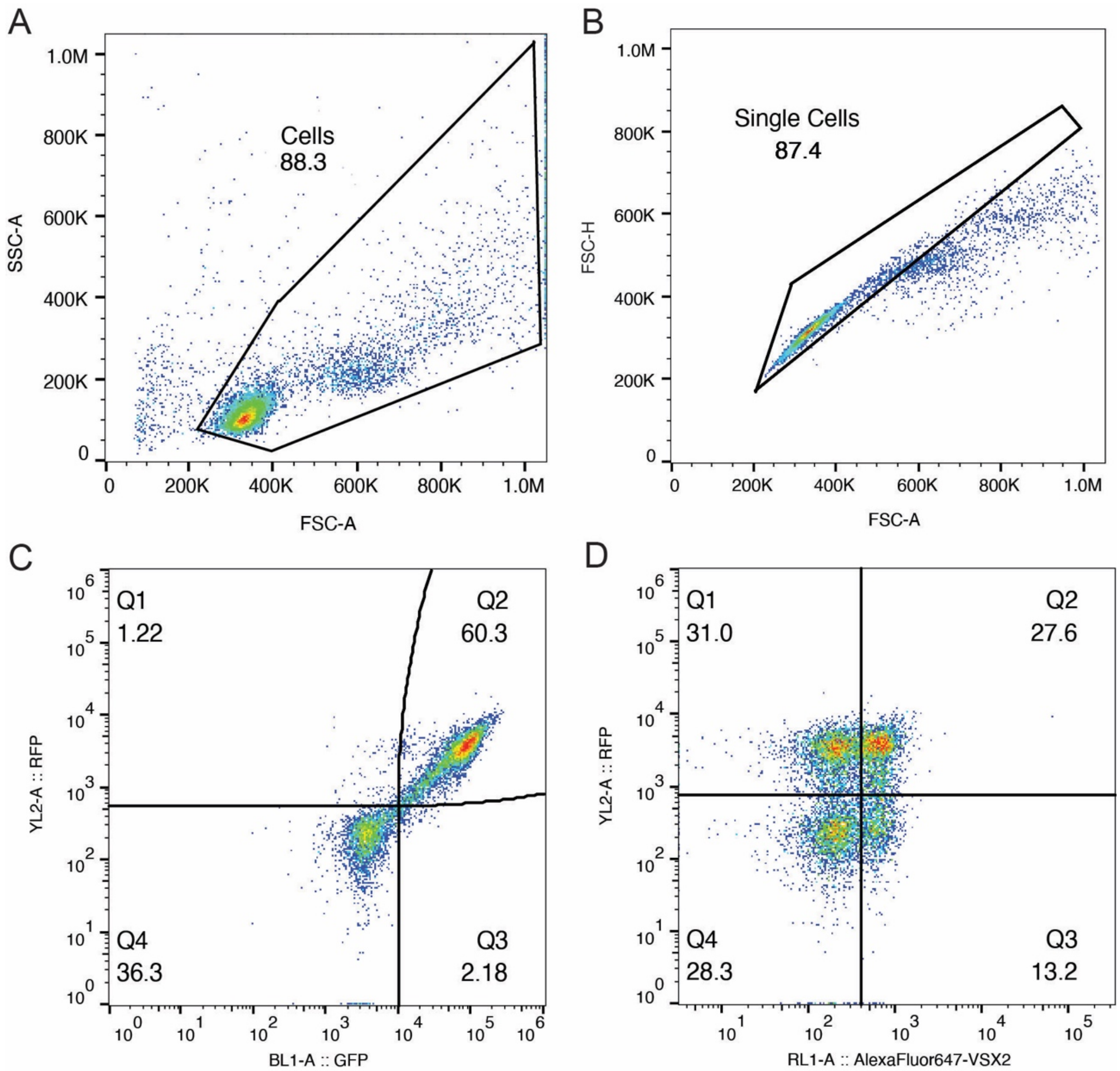

Supplemental Figure 15. Flow cytometry gating strategy for validation of the FOXN4:Cre reporter line.

A. Representative flow cytometry plot of the initial gate on cells to exclude small and very large debris.
B. Representative flow cytometry gating to isolate a single cell population.
C. Representative gating and quantification of the concordance of dual GFP and RFP expression from the Cre-reporter in differentiated neurons.
D. Representative gating and quantification of the frequency of overlap between RFP and VSX2 expression in fixed differentiated neurons.
