# Supplementary material for: Distinct differentiation trajectories leave lasting impacts on gene regulation and function of V2a neurons
Source: Nat Commun. 2026 Jul 29;17:9187. doi: 10.1038/s41467-026-76054-w (PMC13522347; doi:10.1038/s41467-026-76054-w)
Supplement: Supplementary file 3 [file 41467_2026_76054_MOESM2_ESM.pdf]

nature portfolio

Peer Review File

Distinct differentiation trajectories leave lasting impacts on gene regulation and function of V2a interneurons

Corresponding Author: Dr Faranak Fattahi

This file contains all reviewer reports in order by version, followed by all author rebuttals in order by version.

Version 0:

Reviewer comments:

Reviewer #1

(Remarks to the Author)
Elden et al., demonstrate that different developmental trajectories during the differentiation of V2a interneurons from human pluripotent stem cells lead to lasting transcriptomic, epigenetic, and functional differences. By comparing multiome V2a interneuron data generated from two different progenitor populations, neuroepithelial progenitors (NEPs) versus neuromesodermal progenitors (NMPs), the authors show that each lineage establishes unique chromatin landscapes and gene regulatory networks that persist into the later neural stage. NMP-derived V2a neurons exhibit more robust network activity and are enriched for transcription factors that confer spinal cord-like identity, whereas NEP-derived neurons display a broader hindbrain-like signature. However, to prove this critical point for the manuscript, the authors need to include additional data. The study also highlights the limitations of induced V2a neurons (via direct VSX2 overexpression), which display superficial phenotypes but lack stable chromatin modifications necessary for authentic, region-specific identity. While the work highlights how early developmental events impact functional and epigenetic properties, it offers limited novel insights. A main limitation of the study is that it doesn't thoroughly characterize the anterior-posterior identity of the V2a interneurons and motor neurons generated from the two progenitor populations. Additionally, it doesn't prove that the V2a interneurons generated from NEPs and NMPs are better suited for disease modeling or transplantation studies, which would have been the study's most original contribution.

Specific comments:

The cells co-expressing SOX2 and PAX6 in Figure 1F don't represent bona fide neuromesodermal progenitors (NMPs). Instead, these cells have transitioned to a posterior neural progenitor identity. The term "NMP" should be used carefully and consistently in the manuscript, as it can lead to confusion. If the cells have already transitioned, the authors should refer to them as "NMP-derived neural progenitors" or "NMP-derived posterior neurons," depending on their developmental stage.

The data presented in Figure 1C are based on only two biological replicates. It is unclear whether these replicates come from a single iPSC line or multiple lines. Since iPSC lines often vary in their differentiation efficiencies, please clarify whether all lines used in this study generate NMPs with similar efficiency. Such details are essential for the interpretation and reproducibility of the results.

Currently, the results in Figure 1H appear to be pooled across all iPSC lines, potentially obscuring inter-line variability. Showing individual data points for each iPSC line would make it easier to assess biological variation and emphasize the robustness of your conclusions.
Your data indicate that differentiation to V2a interneurons is more efficient when starting from NMP-derived cultures than NEP cultures. Please elaborate on possible mechanistic explanations. For instance, is there a specific signaling environment, transcription factor network, or temporal window that makes NMPs more prone to generate V2a interneurons?

p9: "Also, among the most highly differentially expressed genes were
subunits of cation channels and which could impact synaptic transmission (CACNA2D1 and RYR2 in the NEP condition; CACNA2D3 and KNCD2 in the NEP condition)." Is this correct?

Given the two distinct derivation protocols (anterior vs. posterior), it would be important to describe the anterior-posterior identity of the motor neurons present in the culture. Could the presence of specific progenitors or neurons impact the

efficiency of V2a neuron generation or their functional properties?

In Supplementary Figure 2B, the data appear to show higher expression of posterior HOX genes in the anterior-derived NEPs, which contradicts both the expected lineage specification and your own descriptions in the results section. This is very confusing. Please clarify whether there was an error in labeling, data processing, or interpretation and reconcile it with the overall narrative of more posterior identity arising from NMP-derived cultures.

To strengthen your hypothesis regarding different AP identities, it would be essential to perform immunofluorescence analysis of V2a interneurons generated from the different progenitor populations using HOX markers (e.g., HOXC5, HOXC6, HOXC9). Demonstrating co-localization of these markers in V2a interneurons would be direct evidence supporting your hypothesis of distinct AP identities.

Supplementary Figure 1 shows that replicate 1 has substantially fewer nuclei than replicate 2, and the resulting UMAP appears to lack several clusters found in the second replicate. Please clarify why there is such a discrepancy in the number of nuclei, and discuss whether this might have impacted cluster identification or interpretation of the data. Also, it is not clear why the replicates are analyzed independently, resulting in different UMAPs. To ensure consistent clustering and mitigate batch effects, you should process the data together and then show the UMAPs from each replicate.

The presence of different motor neuron populations introduces a confounding variable that could skew the results and their interpretation. You should provide comprehensive data on the composition of neuronal subtypes in each differentiation protocol, including absolute and relative numbers of motor neurons and V2a interneurons.

Differences in MN populations between NEP-derived and NMP-derived cultures could be driving the functional discrepancies observed in network assays, rather than inherent differences in V2a interneuron properties. This confounding factor complicates the interpretation of results and undermines the conclusion that lineage origin alone is responsible for the functional outcomes.

The authors should consider that the actual difference in the proportions of interneurons to motor neurons might be the reason for the difference in the bursting activity between the NEP and NMP-derived cultures. More interneurons could provide stronger and more coordinated excitatory input to a smaller pool of motor neurons, promoting robust and synchronized bursts.

Minor comments:
a doxycycline-inducible NGN2::P2A::LHX3 cassette and inserted it into stem cells? Which line was used? Please specify in the text.

Reviewer #2

(Remarks to the Author)
The paper by Elder et al., titled "Distinct differentiation trajectories leave lasting impacts on gene regulation and function of V2a interneurons," addresses the question of how a specific neuronal subtype with defined characteristics can emerge from distinct regions of the embryo and, therefore, from molecularly distinct progenitors. This is a highly interesting question, as understanding the processes of phenotypic and molecular canalization during differentiation provides deeper insights into developmental mechanisms. Moreover, it has implications for the generation of specific cell types in vitro for tissue engineering. The paper is well-conducted, and the experiments are clear. However, the disparity between the distinct figures and their conclusions does not strongly support a clear mechanistic message regarding how a neuronal subtype (here the V2a interneurons) can arise through different differentiation pathways. The experiments are conducted in vitro using human pluripotent stem cell-based differentiation and the connection to in vivo processes is lacking. Additionally, the different types of V2a interneurons produced are not compared in a consistent manner.
In this study, the authors identify a novel protocol to generate V2a interneurons from two types of neural progenitors: one displaying a posterior spinal cord progenitor signature, referred to as neuromesodermal progenitors (NMPs), which express TBXT, SOX2, and CDX2, and another population that does not express these markers and is likely more anterior (NEPs). This protocol relies, among other factors, on the activation of the Shh signaling pathway using the Smoothened agonist purmorphamine, as well as Notch pathway inhibition via DAPT. The authors characterize the transcriptional states and chromatin accessibility landscapes at the single-cell level in neurons derived from both progenitor types and demonstrate that the states of V2a interneurons are distinct. Notably, the chromatin regions specifically open in one V2a subtype contain distinct DNA recognition motifs for different transcription factors. Furthermore, genes associated specifically with NMP-derived V2a interneurons are enriched for neuronal identity specifiers, while those associated with NEP-derived V2a are linked to synaptic and axonal growth/guidance regulators. Somewhat disconnected from this finding, the authors observe that neurons derived from NMPs exhibit greater neuronal maturation and higher spontaneous activity than NEP-derived V2a interneurons. They then attempt to generate V2a interneurons through an alternative approach, using genetic engineering—specifically, overexpression of LHX3 and NGN2 in hPSC in the presence of retinoic acid (RA). The resulting neurons display some molecular characteristics of V2a interneurons but lack others, exhibiting molecular differences compared to both NMP- and NEP-derived V2a interneurons. Finally, an in silico gene regulatory network analysis leads the authors to experimentally test the role of several transcription factors in regulating the transcriptional states of V2a interneurons derived from NMP and NEP. They reveal differential dependencies on specific factors, as well as distinct interactions between transcriptional regulators.
Point that can be addressed:

i) The anteroposterior identity of NMP- and NEP-derived V2a interneurons does not align with the expected patterns based on their progenitor origins. Posterior HOX genes, typically characteristic of the spinal cord, are more highly expressed in NEP-derived V2a than in NMP-derived V2a, while the spinal progenitor marker CDX2 is present only in NMPs and absent in NEPs. Can the authors reconcile these findings?
ii) Why does the number of V2a interneurons detected in culture using immunofluorescence not match the number assessed using the single-cell RNA-seq approach? The discrepancy between Figure 1H and Figure S2A should be addressed.
iii) NMP-derived V2a and NEP-derived V2a interneurons appear distinct on UMAPs (Figure 1D). Could they be compared on a single UMAP, alongside in vivo V2a interneurons? Which of these populations most closely resembles in vivo V2a?
iv) The differentially open chromatin regions between NMP-derived and NEP-derived V2a interneurons are presented, but it would be useful to have a description of what is shared between the two, in addition to what is distinct. Could Figure 3 include a balanced representation of both common and distinct features?
v) Why not explore the role of the transcription factors that distinguish NMP-derived V2a from NEP-derived V2a in the last figure? How the last figure connect with the first one and the title message?
vi) Could the differences in transcriptomes account for the variations in neuronal maturation? How do the authors reconcile the results presented in Figure 3 and Figure 4?
vii) The authors propose that the distinct signatures of NMP-derived and NEP-derived V2a interneurons are long-lasting differentiation imprints. However, without characterizing the progenitor signatures, these differences could simply be acquired during differentiation rather than persisting from the progenitor stage. What are the underlying mechanisms?
viii) What was the rationale for exploring a third differentiation pathway?

Version 1:

Reviewer comments:

Reviewer #1

(Remarks to the Author)

The authors have satisfactorily addressed my major concerns. While some limitations remain regarding functional dissection of purified V2a populations, the revised manuscript substantially improves the developmental characterization, bioinformatic rigor, and interpretation of the findings. The remaining limitations are appropriately acknowledged.

Reviewer #2

(Remarks to the Author)
The revised version of the manuscript by Elder et al., entitled “Distinct differentiation trajectories leave lasting impacts on gene regulation and function of V2a neurons”, has improved in clarity and includes additional data and analyses that strengthen the study.

However, several points still require attention:

Page 5 “In vitro, the small molecule agonist purmorphamine (PUR) is substituted for SHH while the gamma-secretase inhibitor DAPT potently inhibits Notch signaling and biases p2 progenitors exclusively toward the V2a fate resulting in almost no V2b cells (as would be identified by GATA2, GATA3, and TAL1 expression).”
The authors should provide a reference to the data demonstrating the distinction between V2a and V2b populations. In addition, V2b neurons are not introduced before this statement. For readers who are not specialists in the field, it would be helpful to briefly describe the common developmental origin of V2a and V2b interneurons in the Introduction.

Page 8 “We also identified a set of peaks which were enriched in the V2a population of both conditions. These shared peaks were largely enriched for homeodomain motifs, especially Paired-related homeodomain factors, of which VSX2 is a member, and contained several genes involved in V2a differentiation, such as LHX4, NKX6.1 and NKX6.2 (Figure 3G; Rayon et al., 2021; Renaux et al., 2024).”

The number of differentially accessible regions (DARs) and shared ATAC peaks between NEP- and NMP-derived V2a neurons should be stated upfront, in the paragraph “To focus on the V2a population, we subset all V2a cells (2672 cells) and performed differential peak analysis. This identified 7128 regions (log2FC > 1 and FDR < 0.05) that were differentially accessible between NEP and NMP V2a populations (Figure 3B).”
The number of common peaks DAR are important because they influence the interpretation and significance of the enrichment analyses. This is particularly relevant given the statement in the Discussion referring to a “minimal overlap” between conditions. Without quantitative values, the term remains subjective.

Page 8 “These results show that the progenitor lineage of V2a neurons leads to lasting differences in the chromatin landscape even after two weeks of identical treatment. Further, these differentially accessible regions are likely to be acted upon by distinct families of transcription factors, suggesting the presence of distinct gene regulatory networks between these two conditions. ”
The conclusions drawn here appear too strong relative to the evidence presented. The analysis is based on motif enrichment, which does not directly demonstrate transcription factor binding or distinct regulatory network activity. Motifs associated with certain transcription factor families (e.g., HOX motifs) can often be found across multiple peak classes. The

authors should therefore be more cautious in their interpretation and clearly distinguish between motif enrichment and functional regulatory activity.

Page 9 “This suggests that the genomic targets resulting from distinct progenitors shift in a context dependent manner.” The term context dependent is too vague and should be clarified. “context” could refer to developmental stage, cell type... specify

Figure 4
Please maintain a consistent color scheme for NEP and NMP populations throughout the figure and across the manuscript.

Page 10 Neuronal activity analysis
The interpretation of the neuronal activity data should be more cautious. The recordings appear to have been performed on bulk neuronal cultures rather than on purified V2a neurons. Therefore, the measured activity likely reflects the combined contribution of multiple neuronal populations present in the culture and cannot be unequivocally attributed to V2a neurons alone.
The manuscript does not sufficiently emphasize the heterogeneous nature of these cultures. Given the presence of additional neuronal subtypes and potentially cells at different maturation states, differences in network activity may arise from changes in population composition rather than intrinsic functional differences within V2a neurons. This limitation should be clearly acknowledged, and the conclusions regarding V2a-specific functional properties should be tempered accordingly.
In addition, the relationship between the qRT-PCR analyses and the functional measurements remains difficult to assess, as the experiments do not appear to be performed at matching developmental time points. The authors should clarify the timing of each analysis and justify how the molecular and functional datasets can be directly compared.

Discussion
The discussion would benefit from a more logical organization, progressing from chromatin accessibility and gene regulatory mechanisms to transcriptional outcomes and, finally, neuronal functional properties.
More importantly, several statements are not adequately discussed in the context of the existing literature. For example, the observation of extensive variability in enhancer accessibility between related neuronal populations has been reported previously in CNS development and should be discussed (see for instance 10.1016/j.neuron.2021.10.014). Additional references would help place these findings in a broader developmental and epigenetic context.
Furthermore, while the authors propose that common transcription factor families may act through distinct enhancer sets, no mechanistic framework is provided to explain how this could occur. The discussion would be strengthened by considering potential mechanisms, such as differential co-factor usage, pioneer factor activity, chromatin priming, or regional chromatin landscapes.

General comment
Although the revised manuscript has improved substantially and the additional data strengthen the study, the manuscript would benefit from further editing to improve clarity and flow. In particular, transitions between sections should be strengthened to better convey the logical progression of the study and clarify how the different datasets support the main conclusions.

Open Access This Peer Review File is licensed under a Creative Commons Attribution 4.0 International License, which

permits use, sharing, adaptation, distribution and reproduction in any medium or format, as long as you give appropriate credit to the original author(s) and the source, provide a link to the Creative Commons license, and indicate if changes were made.
In cases where reviewers are anonymous, credit should be given to 'Anonymous Referee' and the source.
The images or other third party material in this Peer Review File are included in the article's Creative Commons license, unless indicated otherwise in a credit line to the material. If material is not included in the article's Creative Commons license and your intended use is not permitted by statutory regulation or exceeds the permitted use, you will need to obtain permission directly from the copyright holder.
To view a copy of this license, visit https://creativecommons.org/licenses/by/4.0/

We sincerely appreciate the time and effort that the reviewers have dedicated to evaluating our manuscript. We are grateful for their constructive feedback that has now improved the manuscript substantially. Below, we provide detailed responses to each of the reviewers' comments and outline the corresponding changes made in the revised manuscript.

We have highlighted all revisions in the manuscript using blue font to facilitate the review process.

Reviewer #1 Comments:

Elder et al., demonstrate that different developmental trajectories during the differentiation of V2a interneurons from human pluripotent stem cells lead to lasting transcriptomic, epigenetic, and functional differences. By comparing multiome V2a interneuron data generated from two different progenitor populations, neuroepithelial progenitors (NEPs) versus neuromesodermal progenitors (NMPs), the authors show that each lineage establishes unique chromatin landscapes and gene regulatory networks that persist into the later neural stage. NMP-derived V2a neurons exhibit more robust network activity and are enriched for transcription factors that confer spinal cord-like identity, whereas NEP-derived neurons display a broader hindbrain-like signature. However, to prove this critical point for the manuscript, the authors need to include additional data. The study also highlights the limitations of induced V2a neurons (via direct VSX2 overexpression), which display superficial phenotypes but lack stable chromatin modifications necessary for authentic, region-specific identity. While the work highlights how early developmental events impact functional and epigenetic properties, it offers limited novel insights. A main limitation of the study is that it doesn't thoroughly characterize the anterior-posterior identity of the V2a interneurons and motor neurons generated from the two progenitor populations. Additionally, it doesn’t prove that the V2a interneurons generated from NEPs and NMPs are better suited for disease modeling or transplantation studies, which would have been the study’s most original contribution.

Specific comments:

Comment 1:

The cells co-expressing SOX2 and PAX6 in Figure 1F don’t represent bona fide neuromesodermal progenitors (NMPs). Instead, these cells have transitioned to a posterior neural progenitor identity. The term “NMP” should be used carefully and consistently in the manuscript, as it can lead to confusion. If the cells have already transitioned, the authors should refer to them as “NMP-derived neural progenitors” or “NMP-derived posterior neurons,” depending on their developmental stage.

We appreciate this suggestion to clarify our labeling scheme and have revised our figures accordingly. Beyond Figure 1C (see page 16 of the manuscript), cells derived from the NMP lineage are referred to with more specificity throughout the manuscript, such as

NMP-NP or NMP-V2a to indicate NMP-derived neural progenitors or V2a neurons, respectively. The same nomenclature is now used for the NEP lineage.

Comment 2:

The data presented in Figure 1C are based on only two biological replicates. It is unclear whether these replicates come from a single iPSC line or multiple lines. Since iPSC lines often vary in their differentiation efficiencies, please clarify whether all lines used in this study generate NMPs with similar efficiency. Such details are essential for the interpretation and reproducibility of the results.

Thank you for pointing this out. In response to the reviewer's comment, we have included additional data points in Figures 1C and 1D (see page 16 of the manuscript) and clarified what they represent in the figure legend. Each point in these graphs represents a biological replicate from a different cell line, highlighting the robustness of NMP differentiation across cell lines.

Comment 3:

Currently, the results in Figure 1H appear to be pooled across all iPSC lines, potentially obscuring inter-line variability. Showing individual data points for each iPSC line would make it easier to assess biological variation and emphasize the robustness of your conclusions.

We appreciate the request for more clarity on cell line variability. We assume that this comment was intended to point to Figure 1I, since Figure 1H is already grouped by cell line (WTC, FC, WTB, and H9). Data points broken out for each cell line (shown in different shapes) for SOX21 and SOX14 flow cytometry experiments have been included in Figure 1I (see page 16 of the manuscript).

Comment 4:

Your data indicate that differentiation to V2a interneurons is more efficient when starting from NMP-derived cultures than NEP cultures. Please elaborate on possible mechanistic explanations. For instance, is there a specific signaling environment, transcription factor network, or temporal window that makes NMPs more prone to generate V2a interneurons?

This is a valuable observation, and we have revised the manuscript to address this intriguing biological question. We have added the following paragraph to our Discussion section on page 15.

"An interesting observation made from this study was the consistently higher proportion of V2a neurons derived from the caudal NMP progenitors. We speculate that this is due to an interaction of the effect of WNT and SHH signaling. After neural tube closure, WNT and SHH signals are known to antagonize one another to establish the dorsal and ventral

neuron domains. This is at least partially accomplished via WNT-induced expression of GLI3, a SHH repressor (Alvarez-Medina et al., 2008; Yu et al., 2008). Further, there is evidence that WNT effector beta-catenin can bind and sequester the SHH activator GLI1 (Zinke et al., 2015). These potential interactions point to several routes by which induction of WNT signaling during NMP specification may dampen the ability of the NMP-derived neural progenitors to respond to the SHH agonism during V2a differentiation. Together, these would result in a slightly more dorsal-biased population of neurons in the NMP-derived condition and a relatively ventral-biased population of neurons in the NEP-derived condition, as we observed. Importantly, we do not claim that NEP-derived progenitors are innately less able to produce V2a neurons than NMP-derived progenitors, simply that there was a bias under the uniform differentiation condition tested. This interaction of WNT and SHH signaling may also explain why the floor plate population was exclusively present in the NEP-derived population, since SHH signaling from the notochord is necessary to specify floor plate cells in vivo. Hence, the presence of floor plate cells could both exemplify and exacerbate a ventral bias in NEP-derived cultures via their production of SHH ligands."

Comment 5:

On page 9: "Also, among the most highly differentially expressed genes were subunits of cation channels, which could impact synaptic transmission (CACNA2D1 and RYR2 in the NEP condition; CACNA2D3 and KNCD2 in the NEP condition)." Is this correct?

This is indeed a typo which we thank the reviewer for identifying. The genes CACNA2D3 and KCND2 were enriched in the NMP condition, not the NEP condition. The revised text can be found on page 10, paragraph 2.

Comment 6:

Given the two distinct derivation protocols (anterior vs. posterior), it would be important to describe the anterior-posterior identity of the motor neurons present in the culture. Could the presence of specific progenitors or neurons impact the efficiency of V2a neuron generation or their functional properties?

We thank the reviewer for this thoughtful comment. The gene expression pattern in Figure 2B (page 18) and Supplemental Figure 1 (page 27) point to the majority of neurons being of a cranial motor neuron identity expressing PHOX2B and PHOX2A, with only a fraction of MN in the NMP condition expressing the somatic marker HB9. We have clarified these observations in the text on page 6, paragraph 3 and additional supporting graphs have been added as new panel in Supplemental Figure 2A (page 28).

In response to the second comment, there are a few mechanisms which could impact the efficiency of V2a neuron generation, most significantly SHH signaling and Notch signaling (as described and clarified on page 5). In the Discussion on page 15, paragraph 3, we have now noted that the NEP-derived populations contain a fraction of floor plate

cells. These cells which occupy the ventral-most domain of the spinal cord are induced by SHH secreted from the notochord and then secrete SHH themselves. The presence of this population may provide an additive source of SHH signaling in the NEP cultures, further ventralizing the population.

Notch signaling is also an important factor in the generation of V2a neurons, with balanced Notch activation/repression specifying V2b and V2a neurons, respectively. It is also one of the few contact-mediated signals known to influence lineage specification in the spinal cord. However, as noted on page 5, we include the potent Notch pathway inhibitor DAPT in our differentiation which biases all p2 progenitors toward the V2a lineage and away from the V2b lineage. For relative anterior or posterior identity, we rely largely on HOX profile and expression of CDX2 to suggest that the NMP-derived V2a and MNs populations are more caudal, though an exact location in the hindbrain or spinal cord is hard to pinpoint.

Comment 7:

In Supplementary Figure 2B, the data appear to show higher expression of posterior HOX genes in the anterior-derived NEPs, which contradicts both the expected lineage specification and your own descriptions in the results section. This is very confusing. Please clarify whether there was an error in labeling, data processing, or interpretation and reconcile it with the overall narrative of more posterior identity arising from NMP-derived cultures.

We apologies for this error and thank the reviewer for catching it. The referenced figure was mislabeled. Posterior HOX genes are upregulated in the NMP condition, not the NEP condition, in line with expected lineage specification and how the data was described in the text. The corrected figure is on page 28 (now panel 2C).

Comment 8:

To strengthen your hypothesis regarding different AP identities, it would be essential to perform immunofluorescence analysis of V2a interneurons generated from the different progenitor populations using HOX markers (e.g., HOXC5, HOXC6, HOXC9). Demonstrating co-localization of these markers in V2a interneurons would be direct evidence supporting your hypothesis of distinct AP identities.

We appreciate the reviewer's suggestion to perform HOX immunofluorescence in V2a interneurons to strengthen the evidence of distinct AP identities. We fully agree this would be a compelling experiment. However, HOX protein immunostaining is technically challenging due to the high degree of cross-reactivity of antibodies across HOX family members arising from their conserved homeodomain and we would only be able to test a small number of potential targets. As an orthogonal and complementary validation strategy which could encompass all HOX genes, we interrogated our ATAC-seq data to

look at differential chromatin accessibility at HOX cluster loci. We specifically restricted our analysis to the V2a interneuron population to minimize influence of other cell types. We found differentially enriched peaks in the NMP-derived V2a interneuron population in each HOX cluster, amounting to 35 peaks in total. This epigenomic evidence supports distinct AP identities at the level of chromatin architecture and accessibility and is consistent with our transcriptomic findings. We have included these data in the revised manuscript in the Results section on page 8 and as new Supplementary Figure 6.

Comment 9:

Supplementary Figure 1 shows that replicate 1 has substantially fewer nuclei than replicate 2, and the resulting UMAP appears to lack several clusters found in the second replicate. Please clarify why there is such a discrepancy in the number of nuclei, and discuss whether this might have impacted cluster identification or interpretation of the data. Also, it is not clear why the replicates are analyzed independently, resulting in different UMAPs. To ensure consistent clustering and mitigate batch effects, you should process the data together and then show the UMAPs from each replicate.

We would like to acknowledge the concern raised by Reviewer 1 about our approach to cell clustering. We sought to understand the cell identities in each individual sample before merging samples so that the full amount of variation within each sample could be captured. Further, since we have different input cell numbers between our biological replicates, we didn't want to obscure the clustering in the lower cell count samples. The root cause for the discrepancy in cell number between biological replicates is unknown and likely arose during the 10X loading process since each sample was normalized to the same cell number prior. We have added text to the Methods section on page 41 to address this observation.

We used the same list of well-established marker genes to identify each of the populations across all samples which allowed us to confirm the presence of similar cell identities across samples and to collapse similar clusters into single classes. We avoided using a batch correction method for this step since these methods increase the overlap between samples under the assumption that differences largely arise from non-biological variables which may have altered our data interpretation. By integrating samples after cell type identification, as in Figure 2D of the paper, we could confirm that the same identities called on each sample clustered in Harmony UMAP space proximal to one another while also allowing for samples to cluster according to biological differences.

Nonetheless, we took the Reviewer's suggestion and investigated how integration with Harmony before clustering would have impacted the assigned cell identities. For full clarity, we also included our induced-neuron sequencing runs. We first plotted Harmony based clusters in the Harmony-integrated UMAP spaces (Response Figure 1 Left) and then compared how our original cell annotations would have been overlaid on the Harmony UMAP (Response Figure 1 Right) and visualized the distribution of each sample in the Harmony UMAP (Response Figure 2). We next used our panel of marker genes to confirm that each Harmony cluster corresponded to similar cell identities used in the

original manuscript (Response Figure 3 Left-top). We then directly compared the overlap between the Harmony clusters and our original annotations. We found that the majority of cells in each cluster belonged to a single cell identity in our original annotations (Response Figure 3 Left-bottom, Right). Differential gene expression between NEP-V2a neurons and NMP-V2a neurons from cluster 0 of the Harmony integrated UMAP resulted in a very similar gene list to that generated in original analyses (data not shown), lending confidence that our original approach is functionally interchangeable with the one suggested by the reviewer.

We have added text to the methods section on page 41 to support both methods of cluster annotation.

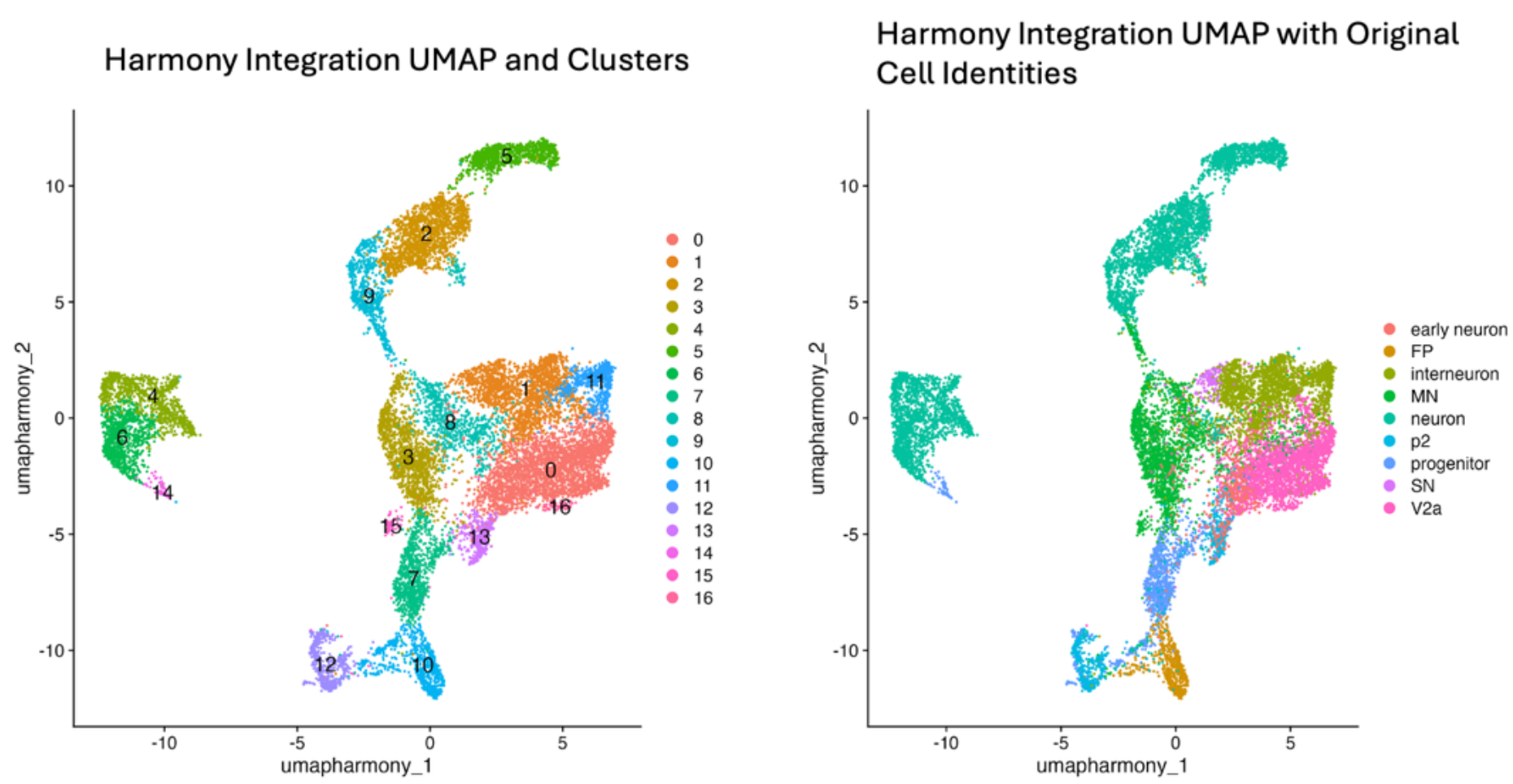

Response Figure 1. Harmony integration of all single-nucleus RNA-seq samples. Left – UMAP of Harmony integrated cells with identified cell clusters. Right – UMAP of Harmony integrated cells colored by the original cell identities used in the manuscript. Note, 'neuron' identity refers to induced neurons at day 4 and 10 (IND-day4 and IND-day10, respectively).

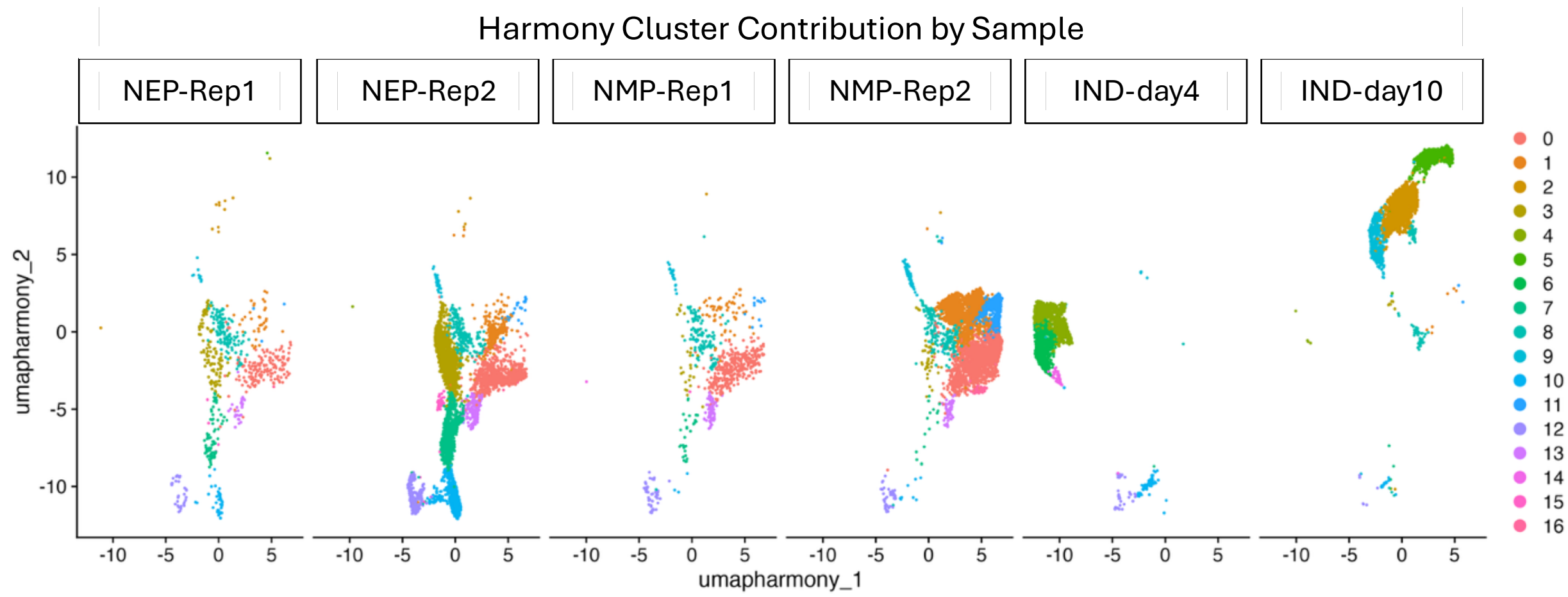

Response Figure 2. Per-sample contribution of each sample to the Harmony Integration UMAP. The same UMAP as plotted in RR Figure 1 (Left), but with each sample identified and split out. Note the overlap of the NEP and NMP replicates and relative separation of the induced samples (IND-day4 and IND-day10).

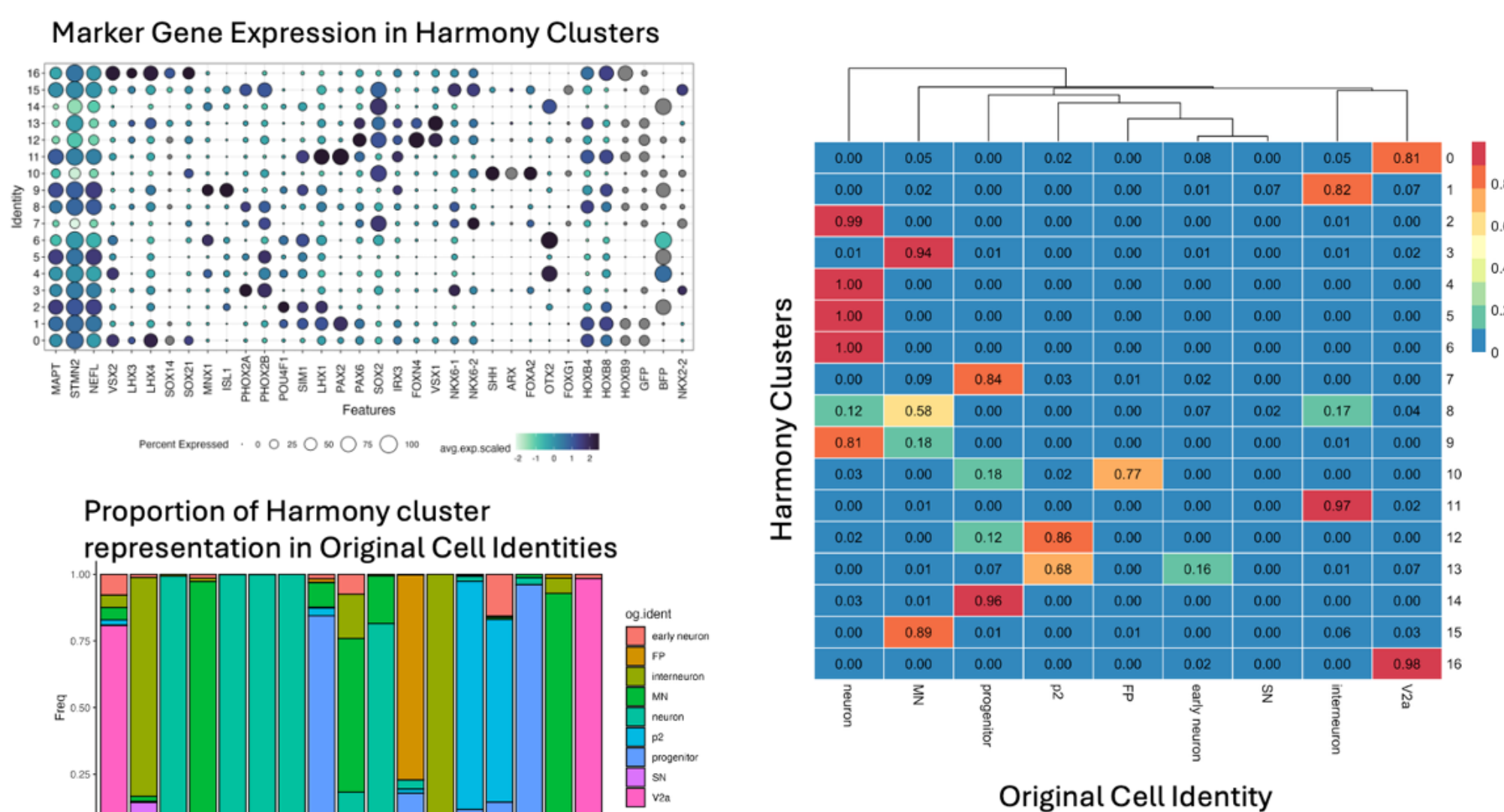

Response Figure 3. Correlation of cell identities with Harmony-based clusters. Left-top – Expression of ventral neural tube marker genes in Harmony cell clusters. Left-bottom – Bar plot representing the proportion of each original cell annotation in each Harmony cell cluster. Note that each cluster contains a major fraction of a single identity. Right – heatmap showing the proportion of each of the original identities in each Harmony clusters. The values in each row sum to 1.

Comment 10:

The presence of different motor neuron populations introduces a confounding variable that could skew the results and their interpretation. You should provide comprehensive data on the composition of neuronal subtypes in each differentiation protocol, including absolute and relative numbers of motor neurons and V2a interneurons.

Thank you for raising this important ponit. In addition to the per-sample UMAPs and marker gene expression plots in Supplemental Figure 1 and the bar graph in Supplemental Figure 2B, we have now included Table S1 outlining the absolute numbers and percentages of each cluster and cell type in all four samples.

Regarding the motor neurons specifically, we see a shift from a predominant PHOX2B/PHOX2A+ phenotype in the NEP-derived samples to one that is also mixed with HB9 (MNX1), LHX4, and HOX expression in the NMP-derived samples, though these MN phenotypes do not necessarily resolve into separate clusters within each sample at the clustering resolution that we used (see new Figure S2A on page 28).

Comment 11:

Differences in MN populations between NEP-derived and NMP-derived cultures could be driving the functional discrepancies observed in network assays, rather than inherent differences in V2a interneuron properties. This confounding factor complicates the interpretation of results and undermines the conclusion that lineage origin alone is responsible for the functional outcomes.

The authors should consider that the actual difference in the proportions of interneurons to motor neurons might be the reason for the difference in the bursting activity between the NEP and NMP-derived cultures. More interneurons could provide stronger and more coordinated excitatory input to a smaller pool of motor neurons, promoting robust and synchronized bursts.

This is an important caveat and we agree that having heterogenous populations complicates the ability to draw direct conclusions. We have revised the Discussion section on page 14, paragraph 3 to explicitly acknowledge this possibility and to frame our conclusions with appropriate caution.

That said, we would like to offer several points that we believe weigh against motor neuron composition as the root cause of the observed functional discrepancies. To your point that differences in the MN population could be driving these differences, another *in vitro* study reported findings that excitatory glutamatergic input, such as that produced by V2a neurons, much more strongly contributes to bursting activity in mixed cultures of motor neurons and interneurons (Sternfeld et al., 2017). The presence of inhibitory interneurons influenced the rate of bursts, typically leading to higher frequency of bursts, but only in the presence of excitatory cells providing the tonic drive. Pure cultures of motor neurons or inhibitory interneurons exhibited little spontaneous activity. We interpret this study to imply that the V2a neurons in our cultures are likely to be a major contributor to overall activity but cannot rule out that other populations may influence the activity. We also

highlight two other example papers (Hägglund et al., 2010 and Wallén-Mackenzie et al., 2006) which find that glutamatergic neurons are at least sufficient, if not necessary, for central pattern generator network stimulation and maintenance.

The ideal experimental design would involve purifying V2a neurons and analyzing them in isolation, without contributions from other cell types in the culture. However, this is currently not technically feasible. On four separate occasions, we performed FACS-based enrichment of V2a neurons differentiated from our FOXN4:CRE reporter line (example images below). Unfortunately, the sorted populations did not survive beyond one to two days post-sort, preventing us from experimentally addressing this question as originally intended. This limitation is consistent with a broader technical challenge in the field, as terminally differentiated neurons are highly sensitive to dissociation and sorting procedures. Accordingly, while our interpretation is supported by prior literature and the overall pattern of our data, we cannot completely exclude the possibility that differences in other neuronal populations contribute to the observed phenotypes. Given this technical constraint, we revised the Discussion to more explicitly acknowledge the potential influence of mixed neuronal populations on the observed phenotypes and to clarify the scope and interpretation of our conclusions.

Post-sort Fractions:

Unsorted GFP+ GFP-

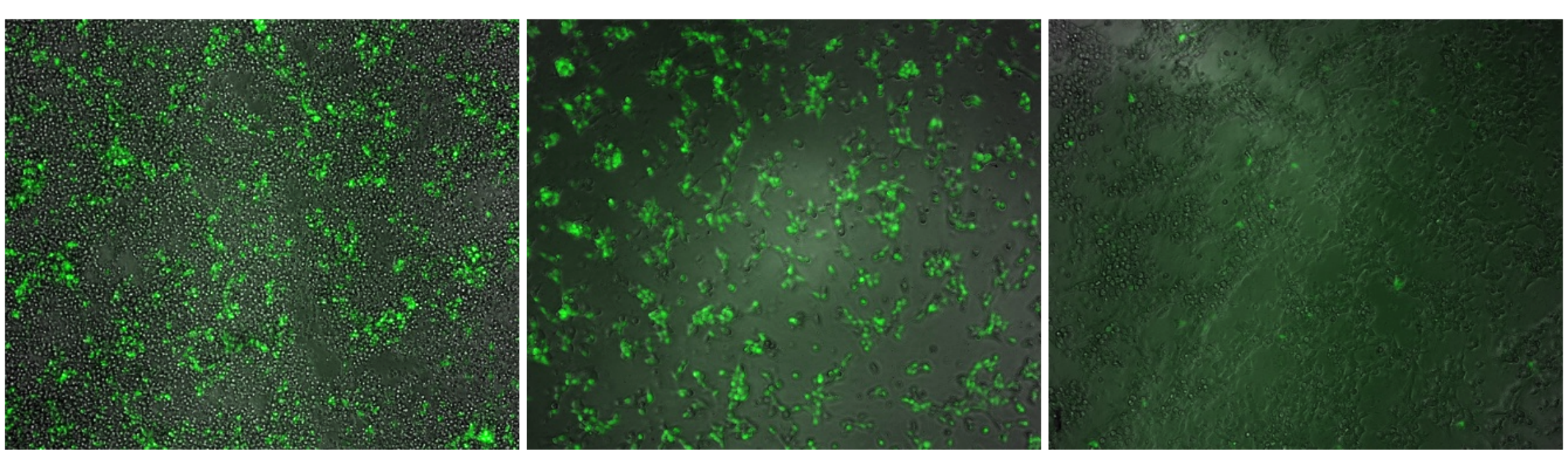

FACS Plot from NEP- (left) and NMP- (right) derived neurons. The FITC+ fraction is enriched for V2a neurons.

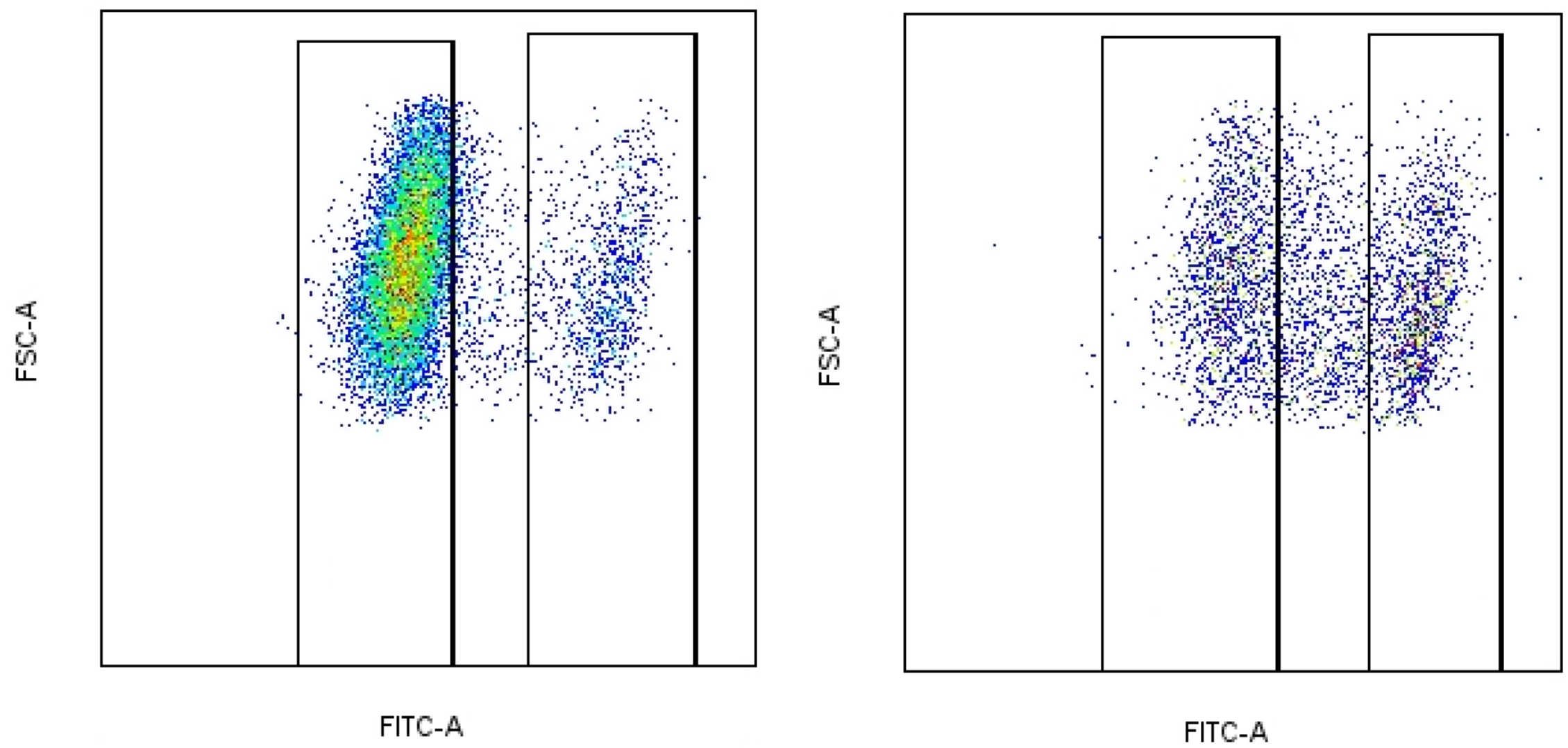

Comment 12:

A doxycycline-inducible NGN2::P2A::LHX3 cassette and inserted it into stem cells? Which line was used? Please specify in the text.

Thank you for the opportunity to provide further clarification about the cell line. The dox-inducible cassette encoding NGN2 and LHX3 in a single ORF was inserted into WTC11 stem cells using piggybac integration. This line was selected because it was included in our single nucleus sequencing experiments, as well as being the background cell line for the FOXN4:CRE reporter line before several editing and subcloning events. This has been specified in the text on page 11, paragraph 1 and in the Methods section on page 43 in the section 'Induced Neuron Cell Line Creation'.

Reviewer #2 Comments:

The paper by Elder et al., titled "Distinct differentiation trajectories leave lasting impacts on gene regulation and function of V2a interneurons," addresses the question of how a specific neuronal subtype with defined characteristics can emerge from distinct regions of the embryo and, therefore, from molecularly distinct progenitors. This is a highly interesting question, as understanding the processes of phenotypic and molecular canalization during differentiation provides deeper insights into developmental mechanisms. Moreover, it has implications for the generation of specific cell types in vitro for tissue engineering. The paper is well-conducted, and the experiments are clear. However, the disparity between the distinct figures and their conclusions does not strongly support a clear mechanistic message regarding how a neuronal subtype (here the V2a interneurons) can arise through different differentiation pathways. The experiments are conducted in vitro using human pluripotent stem cell-based

differentiation and the connection to in vivo processes is lacking. Additionally, the different types of V2a interneurons produced are not compared in a consistent manner.

In this study, the authors identify a novel protocol to generate V2a interneurons from two types of neural progenitors: one displaying a posterior spinal cord progenitor signature, referred to as neuromesodermal progenitors (NMPs), which express TBXT, SOX2, and CDX2, and another population that does not express these markers and is likely more anterior (NEPs). This protocol relies, among other factors, on the activation of the Shh signaling pathway using the Smoothened agonist purmorphamine, as well as Notch pathway inhibition via DAPT. The authors characterize the transcriptional states and chromatin accessibility landscapes at the single-cell level in neurons derived from both progenitor types and demonstrate that the states of V2a interneurons are distinct. Notably, the chromatin regions specifically open in one V2a subtype contain distinct DNA recognition motifs for different transcription factors. Furthermore, genes associated specifically with NMP-derived V2a interneurons are enriched for neuronal identity specifiers, while those associated with NEP-derived V2a are linked to synaptic and axonal growth/guidance regulators. Somewhat disconnected from this finding, the authors observe that neurons derived from NMPs exhibit greater neuronal maturation and higher spontaneous activity than NEP-derived V2a interneurons. They then attempt to generate V2a interneurons through an alternative approach, using genetic engineering—specifically, overexpression of LHX3 and NGN2 in hPSC in the presence of retinoic acid (RA). The resulting neurons display some molecular characteristics of V2a interneurons but lack others, exhibiting molecular differences compared to both NMP- and NEP-derived V2a interneurons. Finally, an in silico gene regulatory network analysis leads the authors to experimentally test the role of several transcription factors in regulating the transcriptional states of V2a interneurons derived from NMP and NEP. They reveal differential dependencies on specific factors, as well as distinct interactions between transcriptional regulators.

Point that can be addressed:

Comment 1:

The anteroposterior identity of NMP- and NEP-derived V2a interneurons does not align with the expected patterns based on their progenitor origins. Posterior HOX genes, typically characteristic of the spinal cord, are more highly expressed in NEP-derived V2a than in NMP-derived V2a, while the spinal progenitor marker CDX2 is present only in NMPs and absent in NEPs. Can the authors reconcile these findings?

The inconsistencies in the data interpretation were caused by a mislabeled figure. We apologize for the confusion and have corrected the figure on page 28. Posterior HOX genes are indeed more highly expressed in the NMP-derived V2a which also upregulated CDX2 early in differentiation, in line with how we described the conditions in the text.

Comment 2:

Why does the number of V2a interneurons detected in culture using immunofluorescence not match the number assessed using the single-cell RNA-seq approach? The discrepancy between Figure 1H and Figure S2A should be addressed.

We understand the need for clarity around the specific cell populations and proportions reported in the paper. For Figure 1H, cells were fixed immediately following dissociation. For single cell experiments, as in Figure S2A (now Figure S2B), populations were subjected to dissociation, cryopreservation, thaw, and nuclear isolation. During that process, it is not unexpected that some of the neurons may have died or been lost, resulting in lower absolute values and enrichment of progenitors. The differences in readout modality (RNA cluster-based identity vs protein expression) may also account for some differences. Lastly, VSX2 can be transiently expressed in V2a neurons, potentially resulting in some cells with detectable protein levels and diminished RNA levels. However, the relative proportional difference between NEP and NMP-derived populations remains consistent across our replicates. We have clarified these points in the manuscript on page 6, paragraph 4:

"While flow cytometry at day 19 showed ~20% VSX2+ cells in NEP and ~50% in NMP cultures (Figure 1H), single-nucleus RNA-seq revealed lower absolute proportions of V2a neurons (Figure S2B), likely reflecting neuronal fragility during cryopreservation and nuclear isolation, as well as differences between protein and RNA detection. Critically, the relative proportion of V2a neurons in NMP- versus NEP-derived cultures was consistent across both methods."

Comment 3:

NMP-derived V2a and NEP-derived V2a interneurons appear distinct on UMAPs (Figure 1D). Could they be compared on a single UMAP, alongside in vivo V2a interneurons? Which of these populations most closely resembles in vivo V2a?

This comment follows much of our initial thinking in seeking to compare *in vivo* and *in vitro* V2a populations and we are glad for the opportunity to address this with more detail. We have added text on page 7 and included this analysis as Figure S3 on page 29 showing our comparison of in vivo human fetal V2a neurons from Rayon et al., 2021 with our in vitro derived V2a neurons.

Briefly, after subsetting our data to focus solely on V2a populations from each replicate, as well as the in vivo neurons, we examined the UMAP space of the populations both with and without sample integration. As one might expect, the in vivo sample (Rayon_V2a; pink color) separated distinctly from the in vitro samples and appeared to largely be driven by the first principal component which accounted for over 12% of the total variation. This prompted us to explore individual PCs with more detail and we found that PC3 produced significant overlap of in vitro and in vivo populations, with in vivo V2a neurons appearing to overlap most with the NMP-V2a neurons. To validate this observation, we took the genes which were positiviely or negatively correlated with PC3

and calculated module scores for each sample. With both of these gene sets, we observed the in vivo V2a neurons scored most similarly to the NMP-V2a neurons.

Comment 4.

The differentially open chromatin regions between NMP-derived and NEP-derived V2a interneurons are presented, but it would be useful to have a description of what is shared between the two, in addition to what is distinct. Could Figure 3 include a balanced representation of both common and distinct features?

We thank the reviewer for their comment. In order to provide a more balanced view of the V2a chromatin landscape, we identified peaks that were significantly enriched in V2a populations relative to all other cells in the same condition (FDR-adjusted p-value < 0.05). We then found the overlapping peak set that was enriched in both NEP-V2a neurons and NMP-V2a neurons and found 3046 peaks enriched in both conditions. This data has been included in Figure 3G and expanded in the text on page 8.

By reprocessing this data, we also identified some instances where differential accessibility testing had occurred for ATAC peaks using the Wilcox method as opposed to the more robust LR method. We repeated all analyses to include the LR method and recalculated motif enrichment for these peak sets. While this changed some of the specifics of the output, the generally observed trends still hold. Lastly, in all figures representing motif enrichment, we changed the order to follow fold change instead of p-value (Figures 3E-F, for example). We felt that fold change captured biological relevance more accurately than magnitude of p-value significance. While this resulted in a different order of motifs presented, it did not change our findings. The text of the Results section on pages 8-9 has been updated to reflect the new graphs, and changes have been made to Figures 3, 4, S4, S5, and S7.

Comment 5:

Why not explore the role of the transcription factors that distinguish NMP-derived V2a from NEP-derived V2a in the last figure? How the last figure connect with the first one and the title message?

We thank the reviewer for seeking more clarity on our experimental design, and specifically the rationale surrounding Figure 6. Instead of characterizing the differences at the point of terminal differentiation, we wanted to know what upstream factors could lead to the end point differences we found in the differentiated V2a neurons. Put another way, if a TF was differentially expressed, we wanted to know why that TF was differentially expressed, not just what its effect was in mature V2a neurons. We also didn't want to assume that this was due to single factors, but potentially also TF complexes or the presence of co-factors. Further, what we observed with the ATAC data was that some families are enriched, but it was difficult to pinpoint which specific member might be actively expressed and producing an effect, if any. We therefore used Cell Oracle to leverage both our RNA and ATAC-seq data not only to account for transcriptional

differences, but also for the relative accessibility of the transcription factor's motif and potential regulatory targets. We knocked down expression of each target gene starting at the stem cell state because we hypothesized that the lineage differences were encoded within the first 5 days of differentiation when the NEP and NMP lineages are specified. This led us to identify CREB5 and TCF7L2 as significantly impacting the NMP-lineage gene signature which may be directly downstream of WNT signaling in the NMP state. In this way, we feel that Figure 6 ties in with the rest of the paper by identifying regulators of the differences between NEP and NMP-derived V2a neurons. We have added text in our Results section (page 12) and Discussion section (page 13 and 14) to further clarify our rationale and findings.

Comment 6:

Could the differences in transcriptomes account for the variations in neuronal maturation? How do the authors reconcile the results presented in Figure 3 and Figure 4?

We thank the reviewer for their feedback and have included data which highlights matching maturation time frames of NEP and NMP-derived interneurons. As stated in the text, because the neural cultures undergo differentiation for the same amount of time, we presume that they are at comparable levels of maturation. To support this, we have added Figure S9, a time course of qPCR marker data showing the acquisition and loss of marker genes between days 5 and 19 of differentiation in both the NEP and NMP-derived V2a protocols. While we cannot be certain that they continue to mature at the same rate, this data shows that they progress similarly up to this point. To further support this, we took a list of marker genes of neuronal function, which includes ion channels, neurotransmitter receptors, and synaptic machinery, published in Ciceri et al. 2024 (their Figure 1O), where they reported the expression of these genes increase as neuron cultures mature. We then applied this gene list as a module score to the conserved cell types between NEP and NMP-derived cultures: p2 progenitors, V2a neurons, motor neurons, and interneurons. Module scoring of this list of 67 genes showed no significant difference between NEP and NMP derived V2a neurons and motor neurons and a small but significant difference in interneurons, with NEP interneurons scoring more highly. Importantly, all three of the neuron classes scored much higher in the maturation module than p2 progenitors, which supports the assumption that this gene list is sufficient to identify differences in maturation. These data have also been included in the paper as Figure 4D and are referenced in the text on page 10. Together, we take the data from Figures 3 and 4 to indicate that there are likely differences in the specific proteins that are used in the synaptic machinery which influence the observed patterns of activity, but that both populations, regardless of progenitor, hit developmental milestones and subsequent maturation stages at roughly equivalent time points.

Comment 7:

The authors propose that the distinct signatures of NMP-derived and NEP-derived V2a interneurons are long-lasting differentiation imprints. However, without characterizing the

progenitor signatures, these differences could simply be acquired during differentiation rather than persisting from the progenitor stage. What are the underlying mechanisms?

Because cells from both conditions were treated with identical media from day 5 onward, we presume that the progenitor stage represents the largest source of variability between the two populations. While we note the presence of a floor plate population in one condition that could shift the relative balance of cell types, we find little evidence in the literature that this would produce cell-intrinsic differences in downstream transcriptional regulation. Importantly, we are not arguing that all specific peaks detected as differentially accessible at the V2a stage are also differentially accessible at the progenitor stage. Supplementary data presented in Figures S7C-D argue against this interpretation, as there is limited overlap between accessible chromatin regions at the p2 and V2a stages within the same condition, suggesting that specific regulatory loci are not simply carried forward unchanged. Instead, what we observe is an enrichment of binding motifs belonging to similar families of transcription factors across both the p2 and V2a stages, implying that the identity of the regulatory family is preserved even as the specific genomic targets shift in a context-dependent manner. Whether a single gene within a given TF family is continuously expressed or whether successive family members hand off regulatory responsibility at each developmental transition remains an open question that would require time-series ATAC-seq data to resolve. Additionally, we cannot rule out a role for early-acting pioneer factors, such as CDX2 or HOX family members, in establishing accessible chromatin regions that subsequently permit stage-appropriate TF binding throughout differentiation. The reviewer correctly notes that we cannot form definitive conclusions without data from earlier time points and we have clarified our language and included this important caveat in our Results (page 9, paragraph 2) and Discussion (page 14, paragraph 2) sections, respectively.

Comment 8:

What was the rationale for exploring a third differentiation pathway?

We appreciate the opportunity to provide more clarity on our rationale for testing induced V2a neurons. An induced V2a neuron would have experienced very minimal developmental patterning being induced from the stem-cell state. Therefore, if we found it to be transcriptionally or epigenetically similar to the NEP-V2a or NMP-V2a neurons, we could have inferred that it resembled a 'default' state, or that the patterning process was secondary to the final gene expression network. We have added language outlining this rationale to page 10 of the manuscript.

We find that induction of NGN2 and LHX3, and even the resulting downstream expression of VSX2 does not recapitulate a V2a identity across several means of characterization (other V2a markers, PCA clustering, gene expression, chromatin profile). However, we think that this serves as a viable model with which to better understand V2a lineage commitment and that closing the gap between a developmentally specified V2a neuron and an induced V2a neuron will allow us to better understand V2a biology. The minimal genetic circuit that we tested could be expanded to include other transcription factors or

developmental signals to improve the fidelity of the V2a network and allow them to become a robust tool in functional or disease studies. More details describing these conclusions has been incorporated into the Discussion on page 15, paragraph 2.

We greatly appreciate the detailed feedback provided by the reviewers and editors. Below, we provide detailed responses to each of Reviewer 2’s comments and outline the corresponding changes made in the revised manuscript. New changes have been highlighted in blue.

REVIEWERS' COMMENTS

Reviewer #2 Remarks to the Author:

The revised version of the manuscript by Elder et al., entitled “Distinct differentiation trajectories leave lasting impacts on gene regulation and function of V2a neurons”, has improved in clarity and includes additional data and analyses that strengthen the study. However, several points still require attention:

Comment 1:
Page 5 “In vitro, the small molecule agonist purmorphamine (PUR) is substituted for SHH while the gamma-secretase inhibitor DAPT potently inhibits Notch signaling and biases p2 progenitors exclusively toward the V2a fate resulting in almost no V2b cells (as would be identified by GATA2, GATA3, and TAL1 expression).”

The authors should provide a reference to the data demonstrating the distinction between V2a and V2b populations. In addition, V2b neurons are not introduced before this statement. For readers who are not specialists in the field, it would be helpful to briefly describe the common developmental origin of V2a and V2b interneurons in the Introduction.

We have introduced V2b neurons on page 3 (paragraph 4) in the introduction as the reviewer suggested, as well as adding an appropriate citation on page 5 (paragraph 2).

Comment 2:
Page 8 “We also identified a set of peaks which were enriched in the V2a population of both conditions. These shared peaks were largely enriched for homeodomain motifs, especially Paired-related homeodomain factors, of which VSX2 is a member, and contained several genes involved in V2a differentiation, such as LHX4, NKX6.1 and NKX6.2 (Figure 3G; Rayon et al., 2021; Renaux et al., 2024).”

The number of differentially accessible regions (DARs) and shared ATAC peaks between NEP- and NMP-derived V2a neurons should be stated upfront, in the paragraph “To focus on the V2a population, we subset all V2a cells (2672 cells) and performed differential peak analysis. This identified 7128 regions (log2FC > 1 and FDR < 0.05) that were differentially accessible between NEP and NMP V2a populations (Figure 3B).”
The number of common peaks DAR are important because they influence the interpretation and significance of the enrichment analyses. This is particularly relevant given the *statement in the Discussion referring to a “minimal overlap” between conditions. Without quantitative values, the term remains subjective.*

We have added specific values into our results section on pages 7-9 (page 7 paragraph 3; page 8 paragraph 1; page 9 paragraph 1) identifying the specific numbers of differentially accessible regions, as well as how many peaks were included in each comparison. We also removed subjective language from the discussion section and included specific values to strengthen our point (page 14, paragraph 2).

Comment 3:
Page 8 "These results show that the progenitor lineage of V2a neurons leads to lasting differences in the chromatin landscape even after two weeks of identical treatment. Further, these differentially accessible regions are likely to be acted upon by distinct families of transcription factors, suggesting the presence of distinct gene regulatory networks between these two conditions. "
The conclusions drawn here *appear too strong relative to the evidence presented*. The analysis is based on motif enrichment, which does not directly demonstrate transcription factor binding or distinct regulatory network activity. Motifs associated with certain transcription factor families (e.g., HOX motifs) can often be found across multiple peak classes. The authors should therefore be more cautious in their interpretation and clearly distinguish between motif enrichment and functional regulatory activity.

We removed 'largely' in the preceding paragraph as an unnecessary distinction of degree.
We also revised last sentence to read: "Further, the motifs enriched in differentially accessible regions correspond to TF families with known roles during V2a development and suggests that they may contribute to distinct gene regulation in each condition." This softens the language and does not conclude any specific binding activity (page 8, paragraph 1).

Comment 4:
Page 9 "This suggests that the genomic targets resulting from distinct progenitors shift in a context dependent manner."
The term context dependent is too vague and should be clarified. "context" could refer to developmental stage, cell type... specify

This sentence was reworded to say: "This suggests that the genomic targets unique to a distinct lineage can change over the course of development." Page 9 paragraph 1.

Comment 5:
Figure 4 - Please maintain a consistent color scheme for NEP and NMP populations throughout the figure and across the manuscript.

Figure 4D has been changed to keep consistency in the color scheme of the NEP and NMP populations.

Comment 6:

Page 10 Neuronal activity analysis - *The interpretation of the neuronal activity data should be more cautious.* The recordings appear to have been performed on bulk neuronal cultures rather than on purified V2a neurons. Therefore, the measured activity likely reflects the combined contribution of multiple neuronal populations present in the culture and cannot be unequivocally attributed to V2a neurons alone.
The manuscript does not sufficiently emphasize the heterogeneous nature of these cultures. Given the presence of additional neuronal subtypes and potentially cells at different maturation states, differences in network activity may arise from changes in population composition rather than intrinsic functional differences within V2a neurons. *This limitation should be clearly acknowledged, and the conclusions regarding V2a-specific functional properties should be tempered accordingly.*
In addition, the relationship between the qRT-PCR analyses and the functional measurements remains difficult to assess, as the experiments do not appear to be performed at matching developmental time points. *The authors should clarify the timing of each analysis and justify how the molecular and functional datasets can be directly compared.*

We add a sentence to acknowledge the limitation of a heterogenous population at the head of the section, as well as in the final paragraph. We also re-organized the structure of the section to more logically introduce the question of maturity in our qPCR and scRNA-seq module scoring data. This required us to re-order the figures as they appear in the section. Lastly, and in line with other of the reviewer's comments, we added language to improve transition and clarity. (pages 9-10)

Comment 7:
Discussion
The discussion would benefit from a more logical organization, progressing from chromatin accessibility and gene regulatory mechanisms to transcriptional outcomes and, finally, neuronal functional properties.

We re-ordered several paragraphs in the discussion (pages 13-16) and added new language to improve transitions.

Comment 8:
More importantly, several statements are not adequately discussed in the context of the existing literature. For example, the observation of extensive variability in enhancer accessibility between related neuronal populations has been reported previously in CNS development and should be discussed (see for instance **10.1016/j.neuron.2021.10.014).** Additional references would help place these findings in a broader developmental and epigenetic context.

We added the following sentence and citations to the Discussion paragraph on ATAC-seq data on page 14, paragraph 2.
"Differences in accessible chromatin over the course of differentiation or between closely related populations has been described in various other CNS models (Velasco et al., 2017; Ziffra et al., 2021; Closser et al., 2022)."

Comment 9: Discussion on mechanism – add language and references
Furthermore, while the authors propose that common transcription factor families may act through distinct enhancer sets, no mechanistic framework is provided to explain how this could occur. The discussion would be strengthened *by considering potential mechanisms, such as differential co-factor usage, pioneer factor activity, chromatin priming, or regional chromatin landscapes.*

We have added this paragraph to the Discussion section (page 14, paragraph 3) to build on potential mechanisms for the different enhancers observed between NEP and NMP populations.

"Identification of the precise mechanism by which changes in progenitors are passed forward to mature neurons requires future time-resolved datasets of ATAC-seq and ChIP-seq data. However, several mechanisms could explain how shared transcription factor families can act through distinct regions of open chromatin between the two V2a populations. First, pioneer transcription factors, such as CDX2 and ASCL1, have been shown to remove repressive histone modifications and directly bind new regions of chromatin (Mazzoni et al., 2013; Păun et al., 2023). Expression of a pioneer factor in a single lineage could open unique regions of chromatin. Further, transcription factor activity has been well described to be modulated by the presence of cofactors which allow for tissue-specific activation, as in the case of PBX and MEIS for HOX genes and ISL1 in the context of LHX3 (Moens & Selleri, 2006; Thaler et al., 2002). Lastly, looking at the genome only through the lens of ATAC-seq limited our analysis to open and actively accessible chromatin. Specific chromatin modifications, like H3K4me1, can label poised enhancers which would be invisible to ATAC-seq and can be written at different enhancer subsets during differentiation (Kubo et al., 2024)."

Comment 10: Improve transitions
General comment
Although the revised manuscript has improved substantially and the additional data strengthen the study, the manuscript would benefit from further editing to improve clarity and flow. *In particular, transitions between sections should be strengthened to better convey the logical progression of the study and clarify how the different datasets support the main conclusions*

In the course of addressing the reviewer's comments above, especially in the section on functional data and the order of the Discussion section, we feel that we have considerably addressed the reviewer's desire to improve clarity and thematic transitions.
