# Supplementary material for: Distinct differentiation trajectories leave lasting impacts on gene regulation and function of V2a neurons
Source: Nat Commun. 2026 Jul 29;17:9187. doi: 10.1038/s41467-026-76054-w (PMC13522347; doi:10.1038/s41467-026-76054-w)
Supplement: Supplementary file 4 [file 41467_2026_76054_MOESM3_ESM.pdf]

Description of Additional Supplementary Files

File: Supplementary Data 1
Description: A list of primers used to construct and verify the XR1 construct integrated into WTC11 human stem cells.

File: Supplementary Data 2
Description: A list of primers used to construct and genotype the FOXN4:Cre reporter construct.

File: Supplementary Data 3
Description: Consolidated information regarding the cluster size and composition in the NEP and NMP Mulitome snRNA-seq and snATAC-seq samples.

File: Supplementary Data 4
Description: A list of primary antibodies used in the study, their supplier, part number and dilution.

File: Supplementary Data 5
Description: A list of oligonucleotides used in the study to generate plasmids that expressed shRNA against the indicated gene.

File: Supplementary Movie 1.
Description: Movie of spontaneous calcium activity reported by Fluo4-AM in day 23 NEP cultures. Video has been pseudo-colored to enhance visibility of fluorescent signal. n=6 independent replicates.

File: Supplementary Movie 2
Description: Movie of spontaneous calcium activity reported by Fluo4-AM in day 23 NMP cultures. Video has been pseudo-colored to enhance visibility of fluorescent signal. n=5 independent replicates.

File: Supplementary Movie 3
Description: Movie of spontaneous calcium activity reported by Fluo4-AM in day 43 NEP cultures. Video has been pseudo-colored to enhance visibility of fluorescent signal. n=6 independent replicates.

File: Supplementary Movie 4

Description: Movie of spontaneous calcium activity reported by Fluo4-AM in day 43 NMP cultures. Video has been pseudo-colored to enhance visibility of fluorescent signal. n=5 independent replicates.
